# A green approach to 2-arylquinolines *via* palladium-catalysed C–H activation

**DOI:** 10.1039/d5ra05924k

**Published:** 2025-11-04

**Authors:** Nuno Viduedo, Leonardo Pirvu, Ângelo Alves, Fábio Santos, M. Manuel B. Marques

**Affiliations:** a LAQV@REQUIMTE, Department of Chemistry, NOVA School of Science and Technology, Universidade Nova de Lisboa 2829-516 Caparica Portugal; b Centro Research Institute for Medicines (iMed.ULisboa), Faculty of Pharmacy, Universidade de Lisboa 1649-003 Lisboa Portugal

## Abstract

A mild, palladium-catalyzed C–H activation enables direct, sustainable synthesis of 2-arylquinolines from simple allyl amines, with up to 64% yield; green chemistry metrics highlight its superior atom economy and reduced environmental impact compared to conventional methods.

## Abstract 2

A mild, palladium-catalyzed C–H activation enables direct, sustainable synthesis of 2-arylquinolines from simple allyl amines, with up to 64% yield; green chemistry metrics highlight its superior atom economy and reduced environmental impact.

## Graphical abstract

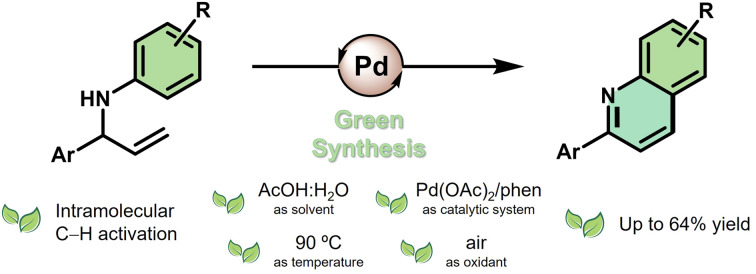

## Introduction

Quinolines are a major class of heterocycles in organic chemistry, found widely in natural products, pharmaceuticals, and functional materials.^1^ Their scaffold appears in biologically active compounds like quinine and camptothecin, showing antimalarial, antitumor, anti-inflammatory, and analgesic effects.^2–4^ Synthetic quinoline derivatives are also important in drug discovery and industrial uses such as agrochemicals, antiseptics, and corrosion inhibitors.^5,6^

**Fig. 1:**
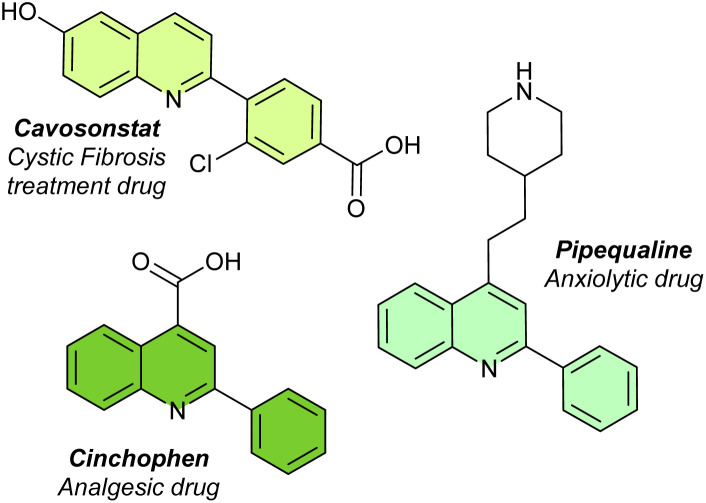
Examples of bioactive 2-arylquinoline derivatives.

The 2-arylquinoline subclass is especially significant for its pharmacological and industrial roles (Fig. 1). Compounds with this structure exhibit activities like P-selectin antagonism, DNA intercalation, and enzyme inhibition, with linsitinib being notable in cancer therapy.^7–10^ They also serve essential functions in catalysis and materials science as ligands and precursors to photoactive materials.^11–13^

The structural and functional diversity of quinolines has spurred significant research into developing efficient methods for their synthesis. Historically, classical strategies such as the Skraup, Friedländer, and Doebner–Miller reactions, as well as the Povarov reaction followed by oxidation, have been widely employed.^14–17^ These methods typically involve multi-step processes, harsh reaction conditions, or stoichiometric oxidants, often resulting in low atom economy and the generation of toxic byproducts.

**Scheme 1:**
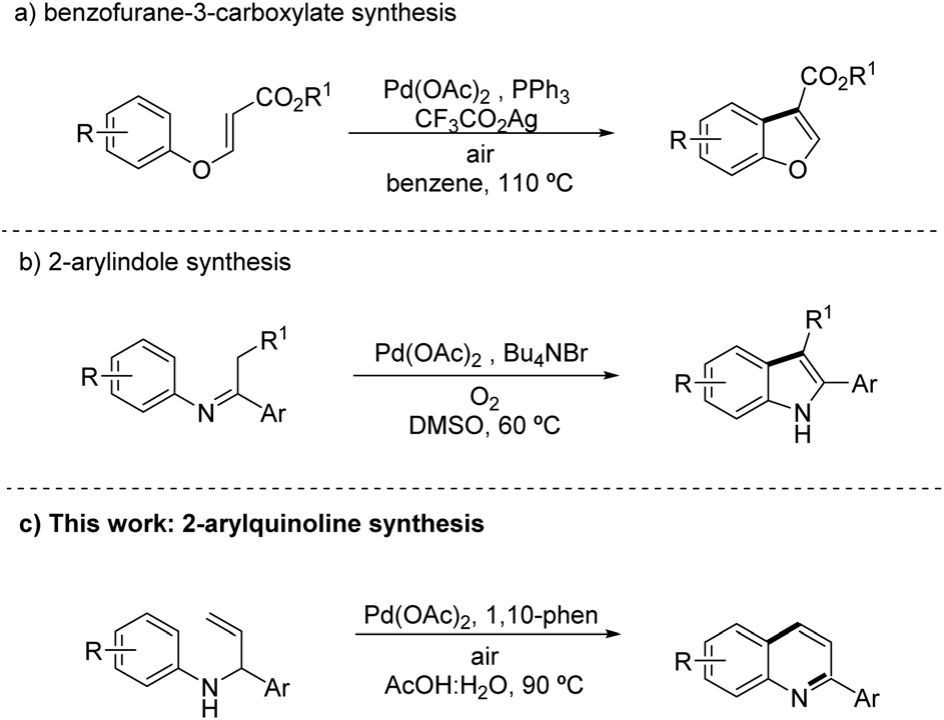
*N*-Heterocycles synthesis *via* Pd-catalysed intramolecular C–H activation.

In recent years, the pursuit of more sustainable and efficient synthetic methodologies has prompted the development of various photo- and metal-catalysed strategies.^18^ Among these, palladium-catalysed C–H functionalization has emerged as a particularly versatile approach, enabling the direct construction of heterocyclic frameworks from readily available substrates, without the need for pre-functionalization or stoichiometric reagents. In this context, in 2011, Li and Wang demonstrated the intramolecular palladium-catalysed oxidative C–H activation of 3-phenoxyacrylates to access benzofuran-3-carboxylates (Scheme 1a), illustrating the potential of this strategy for oxygen-containing heterocycles.^19^ The following year, Yoshikai and co-workers applied a related approach for the synthesis of 2-arylindoles from imine/enamine substrates (Scheme 1b) under mild conditions, further highlighting the utility of intramolecular C–H activation for heterocycle construction.^20^ Inspired by these advances, we develop a new palladium-catalysed intramolecular C–H activation protocol for the synthesis of 2-arylquinolines (Scheme 1c), an important scaffold in medicinal chemistry, under mild and environmentally friendly conditions.

## Results and discussion

### Optimization studies

The investigation was initiated by using the reaction of 1a as a model system to screen the optimal conditions. As shown in Table 1, the absence of catalyst resulted in no reaction (entry 1), while the use of catalyst without ligand led to only trace amounts of quinoline 2a (entry 2). Performing the reaction at 130 °C in acetic acid with Pd(OAc)_2_ and 1,10-phen as the catalyst and ligand, respectively, under an air atmosphere, resulted in a limited yield of 26% (entry 3). Solvent screening revealed that AcOH was the most effective solvent, as DMSO, HFIP, and PivOH all provided significantly lower yields (entries 4–7). The use of oxidants, such as Cu(OAc)_2_ and Ag_2_CO_3_, resulted in yields of 22% and 21%, respectively (entries 8 and 9), showing no advantage over the reaction conditions using air. Fine-tuning the reaction temperature was pivotal. At 130 °C, the reaction was hindered by the competing formation of a side product *via* an aza-Cope rearrangement. Reducing the temperature to 90 °C minimized this undesired pathway, enhancing the selectivity and resulting in a 49% yield for the desired product (entry 11). Deviations from this temperature led to less efficient results (entries 10 and 12). Replacing air with oxygen as the oxidant did not improve the reaction outcome (entry 13). Exploring different palladium catalysts revealed that Pd(acac)_2_ performed compa-rably to Pd(OAc)_2_ (entry 14). Ligand screening revealed that nitrogen-based ligands, such as 1,10-phen and Bphen, exhibited comparable and significantly higher efficiencies compared to the phosphine ligand PPh_3_, which drastically reduced the reaction yield (entries 15 and 16). A reduced ligand-to-catalyst ratio showed no impact, maintaining the yield at 49% (entry 17). In contrast, doubling the palladium loading from 10 mol% to 20 mol% resulted in an improved yield of 57% (entry 18). Further dilution of the reaction medium to 0.025 M enhanced the outcome, achieving a yield of 60% (entry 19). Replacing AcOH with a mixture of AcOH : H_2_O (9 : 1) provided the highest yield of 64% (entry 20), demonstrating the beneficial effect of water on the reaction. Additional optimization experiments were conducted, and the full details can be found in the SI, Pages 7–10.

**Table 1:** Optimization table^a^

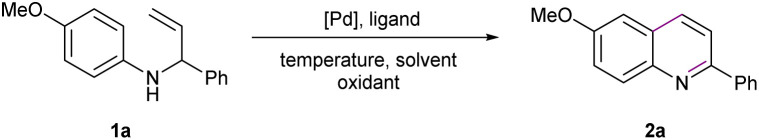					
Entry	Catalyst | ligand	Temperature	Solvent	Oxidant	Yield^b^
1	—| 1,10-phen	130 °C	AcOH	Air	—
2	Pd(OAc)_2_ | -	130 °C	AcOH	Air	Trace
3	Pd(OAc)_2_ | 1,10-phen	130 °C	AcOH	Air	26%
4	Pd(OAc)_2_ | 1,10-phen	130 °C	DMSO	Air	—
5	Pd(OAc)_2_ | 1,10-phen	130 °C	DMSO : AcOH (4 : 1)	Air	7%
6	Pd(OAc)_2_ | 1,10-phen	130 °C	HFIP	Air	Trace
7	Pd(OAc)_2_ | 1,10-phen	130 °C	PivOH	Air	11%
8	Pd(OAc)_2_ | 1,10-phen	130 °C	AcOH	Cu(OAc)_2_	22%
9	Pd(OAc)_2_ | 1,10-phen	130 °C	AcOH	Ag_2_CO_3_	21%
10	Pd(OAc)_2_ | 1,10-phen	110 °C	AcOH	Air	45%
11	Pd(OAc)_2_ | 1,10-phen	90 °C	AcOH	Air	49%
12	Pd(OAc)_2_ | 1,10-phen	70 °C	AcOH	Air	41%
13	Pd(OAc)_2_ | 1,10-phen	90 °C	AcOH	O_2_	47%
14	Pd(acac)_2_ | 1,10-phen	90 °C	AcOH	Air	48%
15	Pd(OAc)_2_ | Bphen	90 °C	AcOH	Air	46%
16	Pd(OAc)_2_ | PPh_3_	90 °C	AcOH	Air	10%
17^c^	Pd(OAc)_2_ | 1,10-phen	90 °C	AcOH	Air	49%
18^d^	Pd(OAc)_2_ | 1,10-phen	90 °C	AcOH	Air	57%
19^e^	Pd(OAc)_2_ | 1,10-phen	90 °C	AcOH	Air	60%
20^e^	Pd(OAc)_2_ | 1,10-phen	90 °C	AcOH : H_2_O (9 : 1)	Air	64%

a Unless otherwise noted, all reactions were performed with 1a (0.10 mmol), Pd catalyst (10 mol%), ligand (20 mol%), oxidant (1 atm or 1 equiv.) in the indicated solvent (0.1 M) for 24 h.

b Determined by ^1^H NMR, using 1,3,5-trimethoxybenzene as the internal standard.

c [ligand]/[Pd] = 1.

d [Pd] loading = 20 mol%.

e [solvent] = 0.025 M.

### Reaction scope evaluation

With the optimal reaction conditions established, the substrate scope and limitations of the methodology were evaluated (Table 2). Starting from the model substrate leading to 2a (bearing a methoxy substituent on the aniline ring), the unsubstituted 2-arylquinoline derivative 2b (R = H) was obtained in a lower yield of 46%. A more pronounced decrease in efficiency was observed for substrates bearing electron-withdrawing groups, such as 2c (R = Cl) and 2d (R = CF_3_), which afforded the corresponding products in 38% and 11% yield, respectively. These results suggest that decreased nucleophilicity of the aniline ring negatively affects the reaction outcome, potentially due to a less favourable initial coordination to the palladium catalyst. Conversely, substrates bearing strongly electron-donating groups, such as trimethoxyphenyl (2e) and methylthio (2f), performed significantly better, affording the desired quinolines in 60% and 55% yield, respectively. Similar yields were observed for 2g (R = phenyl) and 2h (R = methylenedioxy), which provided the corresponding products in 59% yield each. These findings further support the hypothesis that electron-rich substituents enhance the reactivity under the developed conditions. The scope was further extended to fused aromatic systems and quinoline nitrogenated analogues. The benzo[*f*]quinoline 2i was obtained in 35% yield, while the 1,5-naphthyridine derivative 2j was isolated in 36% yield, demonstrating the applicability of the methodology beyond simple quinoline scaffolds.

**Table 2:** Scope of the reaction^a^^,^^b^

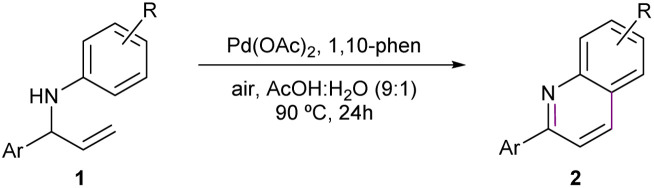
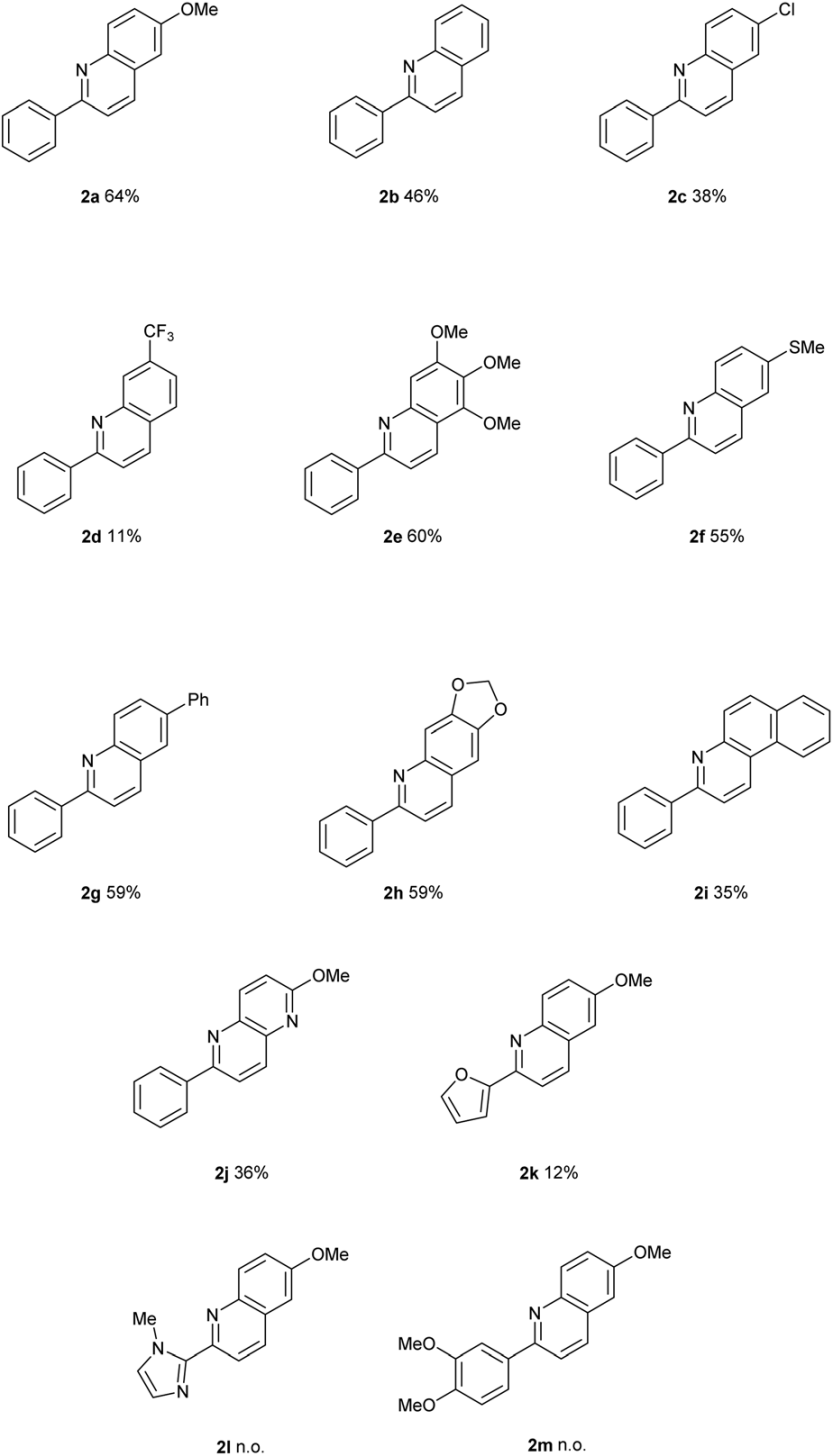

a Standard conditions: 1 (0.25 mmol), Pd(OAc)_2_ (20 mol%), 1,10-phen (20 mol%), AcOH : H_2_O (9 : 1, 0.025 M), 90 °C, under air, 24 h.

b Isolated yields are reported.

Throughout the entire substrate set, the reaction exhibited excellent regioselectivity. In most cases, only one regioisomer was detected. Minor amounts (<10%) of the alternative regioisomer were observed only in the cases of 2d and 2h (SI, structures 2d′ and 2h′).

Finally, variations on the 2-aryl moiety were explored. Replacement of the phenyl group by a furanyl group resulted in a dramatic drop in efficiency, affording quinoline 2k in only 12% yield. The use of 1-methyl-1*H*-imidazole-5-yl as a new aryl group failed to provide the desired product 2l under the optimized conditions. The 3,4-dimethoxyphenyl moiety also proved unreactive under the reaction conditions, and no quinoline 2m was detected.

The synthesized quinolines constitute attractive scaffolds for further functionalization, as demonstrated in the literature for similar systems, particularly through metal-catalysed cross-coupling reactions.^21^ In this context, the 6-chloro-2-phenylquinoline (2c) is of special interest, since the chloro substituent provides an additional reactive site that can broaden the range of possible transformations.

### 1 mmol-scale synthesis of quinoline 2a

**Scheme 2:**
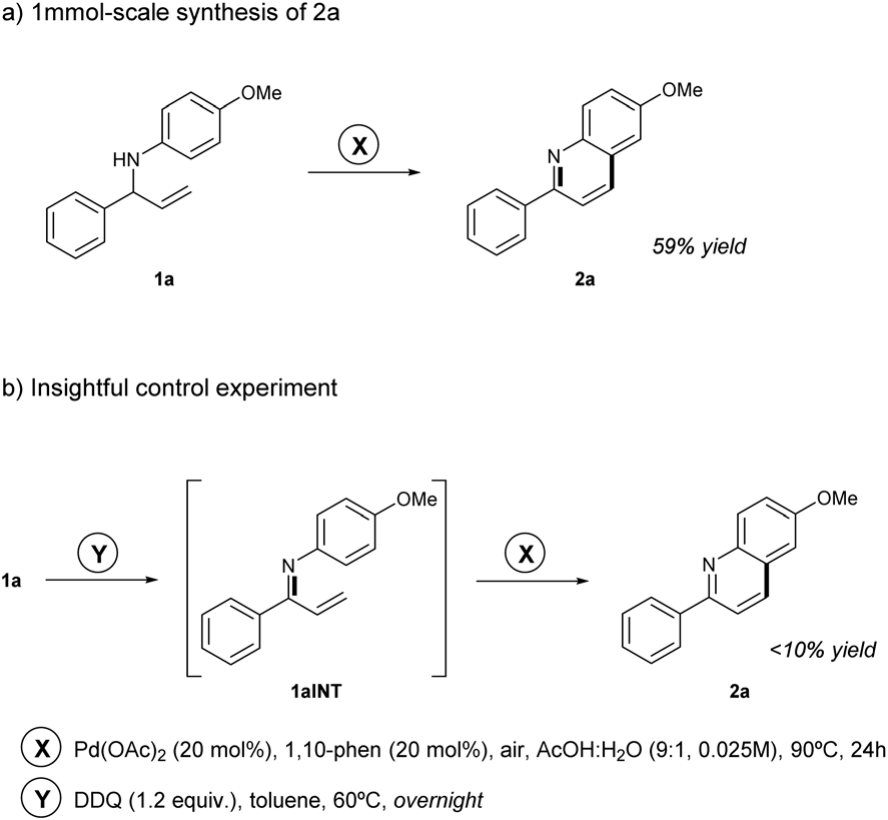
(a) Scalability and (b) mechanistic insight experiments under optimized conditions.

A 1 mmol scale experiment using amine 1a was conducted to confirm the robustness and scalability of the methodology, yielding 2-arylquinoline 2a in 59% (Scheme 2a), demonstrating consistent performance with the previously observed yield on smaller scale.

### Insightful control experiment

To gain further insight into the reaction mechanism, a control experiment was performed in which the allylic amine 1a was pre-oxidized to the corresponding imine using DDQ (Scheme 2b), with the consumption of the starting material and the formation of the putative imine intermediate confirmed by TLC analysis. The resulting imine was then subjected to the standard reaction conditions developed in this work. A marked decrease in efficiency was observed under these conditions, affording the 2-arylquinoline 2a in less than 10% yield. This result implies that the presence of the C–N single bond, or the adjacent benzylic C–H, are essential for reactivity, highlighting their involvement in the mechanism.

### Proposed reaction mechanism

**Scheme 3:**
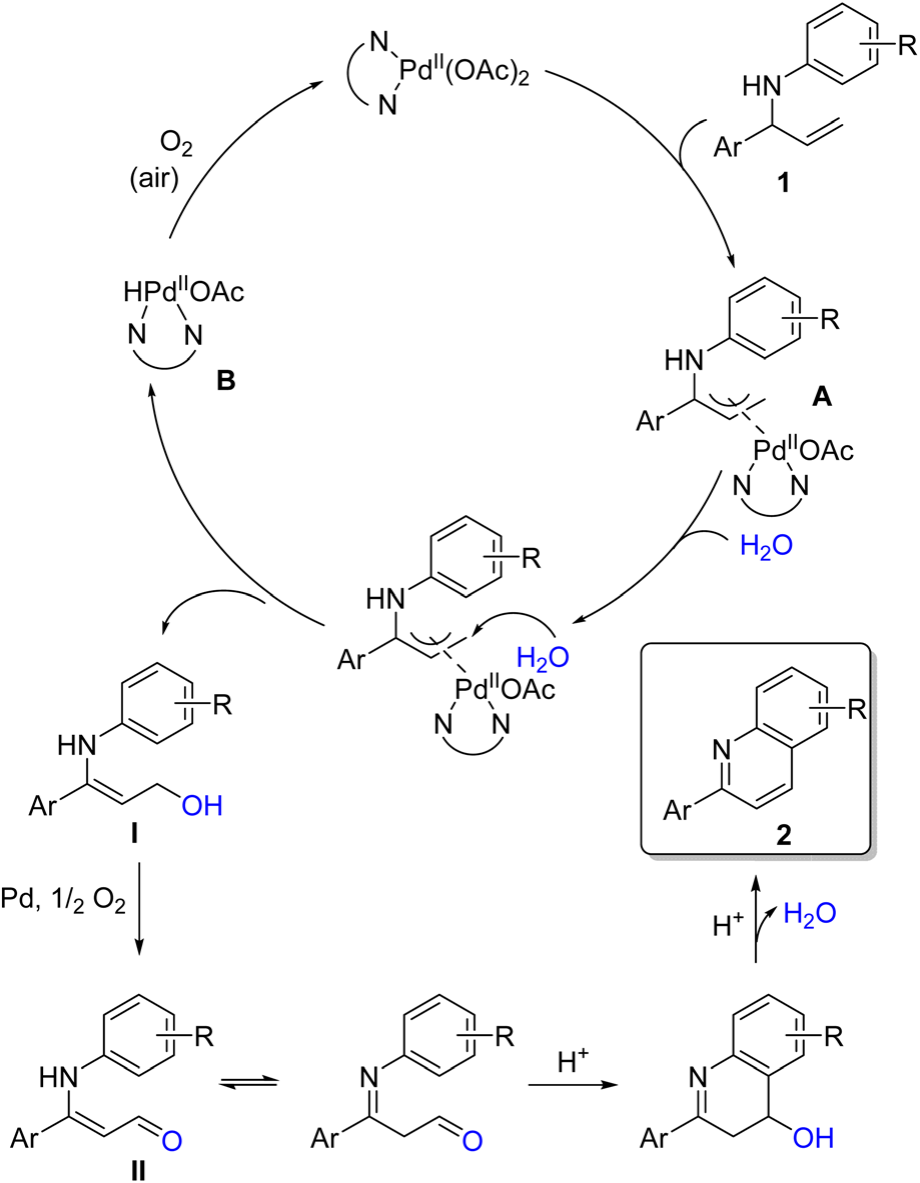
Proposed reaction mechanism.

The proposed mechanism (Scheme 3) initiates with the coordination of the Pd(OAc)_2_/1,10-phenanthroline complex to the double bond of the allylic amine substrate, forming a π-allylpalladium complex A. This coordination is essential and is not possible when the substrate is pre-oxidized to the imine, consistent with the observed inhibition under such conditions (see Scheme 2b). Subsequent nucleophilic attack by water at the terminal carbon of the π-allyl moiety leads to the displacement of the palladium species, yielding the corresponding allylic alcohol I while generating the HPd(OAc)(phen) complex B.^22^ The allylic alcohol I then undergoes palladium- and oxygen-mediated oxidation under the reaction conditions to furnish the corresponding aldehyde II (evidence of the aldehydic proton observed by crude NMR). Electrophilic attack of the aryl ring on the aldehyde, facilitated by the acidic medium and promoted by electron-donating substituents (as noted by the higher yields observed in those cases), is followed by elimination and rearomatization to yield the final 2-arylquinoline product 2.

### Green metrics evaluation

Table 3 provides a side-by-side comparison of selected green chemistry indicators, enabling an objective evaluation of the present method (d) against established protocols (a–c).^22–24^ The reported isolated yields vary from 44% (entry b) to 80% (entry a), with the current methodology achieving a moderate yield of 64%. Atom economy values are generally high across all methods, ranging from 79% (entry b) to 98% in the present work. Reaction mass efficiency (RME), which combines atom economy with yield, displays more variation: entry a shows the highest RME (73%), while entry b performs poorly (25%). The RME of the current method is 63%, reflecting efficient incorporation of materials into the final product. The PMI_RRC_ is also included as a metric that reflects the material input required relative to the molecular complexity of the transformation. Entry b presents the highest PMI_RRC_ value (8.0 g g^−1^), indicating a higher material demand relative to product output and complexity, while entry a exhibits the most favourable value (1.5 g g^−1^). The current methodology shows a PMI_RRC_ of 2.1 g g^−1^, lower than entries b and c, indicating a relatively efficient process in terms of reagent and catalyst usage.

**Table 3:** Comparison of Green Chemistry Metrics in the synthesis of 6-methoxy-2-phenylquinoline for literature methods and the present work

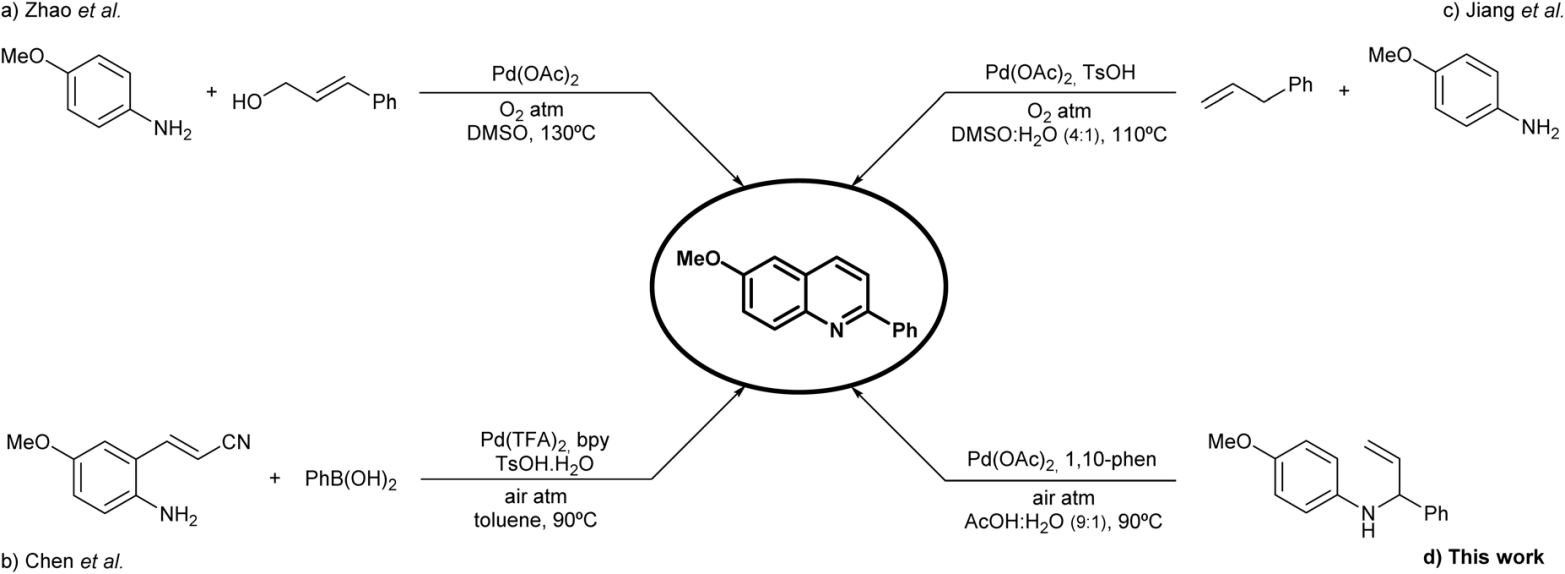				
Green metrics	Synthetical approach			
	(a)	(b)	(c)	(d) This work
Yield (%)	80	44	73	64
Atom economy (%)	91	79	97	98
RME (%)	73	25	48	63
PMI_RRC_ (g g-1)	1.5	8.0	2.4	2.1
Solvent	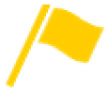	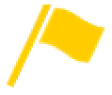	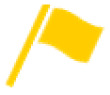	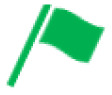
Energy	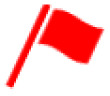	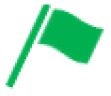	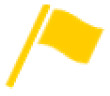	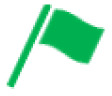
Oxidant	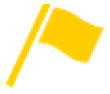	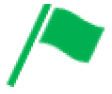	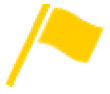	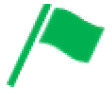

In addition to quantitative parameters, qualitative aspects such as solvent choice, energy input, and oxidant selection are assessed using a three-level colour code: green (favourable), yellow (intermediate), and red (unfavourable). Regarding solvent choice, all literature methods (a–c) are flagged yellow. Entry a employs DMSO, and entry b uses toluene—both commonly used but with environmental or safety concerns. Entry c uses a DMSO : H_2_O mixture, slightly mitigating but not eliminating concerns associated with DMSO. In contrast, the current method (entry d) uses an AcOH : H_2_O system, considered more environmentally benign and therefore classified as green.

For energy input, entry a is marked red, as the reaction proceeds at 130 °C, indicating high energy demand. Entry c operates at 110 °C and is thus rated yellow. Both entry b and the present method (d) operate at 90 °C and are considered energetically favourable, earning green classifications.

Finally, oxidant selection is also considered. Entry a and entry c use molecular oxygen as the terminal oxidant, leading to an intermediate (yellow) rating due to potential handling considerations. Entry b and the current method (d) employ ambient air, which is the most benign and sustainable oxidant source, earning both entries a green classification.

## Conclusions

A palladium-catalyzed C–H activation approach for the synthesis of 2-arylquinolines has been developed enabling direct heterocycle construction from readily available substrates without pre-functionalization or stoichiometric reagents. The method proceeds under mild, environmentally friendly conditions and affords up to 64% yield across 12 examples. Offering high atom economy and reduced ecological impact, it presents a sustainable alternative to conventional routes. Green chemistry metrics were applied to evaluate its environmental performance and benchmark it against literature methods, underscoring the need to assess greenness early in the discovery process using appropriate parameters and guidelines.

## Conflicts of interest

There are no conflicts to declare.

## Supplementary Material

RA-015-D5RA05924K-s001

## Data Availability

All data supporting the findings of this study, including experimental procedures, characterization data, and green chemistry metrics, are available within the article and its supplementary information (SI) file. Additional raw data can be made available from the corresponding author upon reasonable request. See DOI: https://doi.org/10.1039/d5ra05924k.
