# Supplementary material for: A green approach to 2-arylquinolines via palladium-catalysed C–H activation
Source: RSC Adv. 2025 Nov 4;15(50):42588–93. doi: 10.1039/d5ra05924k (PMC12584958; doi:10.1039/d5ra05924k)
Supplement: RA-015-D5RA05924K-s001 [file RA-015-D5RA05924K-s001.pdf]

Supplementary Information (SI) for RSC Advances.
This journal is © The Royal Society of Chemistry 2025

Supplementary Information

A Green Approach to 2-Arylquinolines via Palladium-Catalysed C–H Activation

Nuno Viduedo,[a] Leonardo Pirvu,[a] Ângelo Alves,[a] Fábio Santos,[b] and M. Manuel B. Marques*[a]

Contents

1. General Information…………………………………………………………………….3
2. Green Metrics…………………………………………………………………………….4
    2.1. Formulas………………………………………………………………………4
    2.2. Calculations……………………………………………………………………5
3. Optimization Studies………………………………………………………………………..7
4. Synthesis of Allylic Amines (Starting Materials)…………………………………….11
    4.1. General Procedure…………………………………………………………..11
    4.2. Characterization………………………………………………………..12
5. Synthesis of 2-Arylquinolines………………………………………………………21
    5.1. General Procedure…………………………………………………………..21
    5.2. Characterization………………………………………………………..21
6. NMR Spectra…………………………………………………………………………….26

General Information

Various commercially available purchased reagents were employed and used without further purification, except when indicated. Solvents, when referenced, were pre-dried using conventional methods. 3Å molecular sieves were activated by heating at 300 °C in a muffle furnace for 3 h. Analytical TLC was performed on Macherey-NaGel-0.20 mm silica gel 60 with fluorescent indicator UV254 supported on aluminium. Normal phase silica gel flash chromatography was carried out using Carlo Erba silica gel 60 Å and the described eluent system for each case. IR spectra were acquired using a Perkin-Elmer Spectrum Two FT-IR spectrometer equipped with a UATR module. Transmittance of the samples were acquired between 4000 and 400 cm^{-1}, with resulting IR bands reported in cm^{-1} and categorized as weak (w), medium (m) or strong (s). NMR spectra were measured with a Bruker ARX 400 spectrometer. ^{1}H NMR, ^{13}C NMR and ^{19}F NMR were acquired at 400, 101 and 376 MHz, respectively. Samples were prepared in 0.5 mm NMR tubes using $CDCl_3$ as solvent. NMR data were characterized in terms of chemical shift in ppm (with the corresponding trace $CHCl_3$ used as reference signal), multiplicity, coupling constants (J) in Hz, and integration. Signal multiplicity was denoted as singlet (s), broad singlet (br. s), doublet (d), dd (doublet of doublets), dt (doublet of triplets), ddd (doublet of doublets of doublets), t (triplet), td (triplet of doublets), q (quartet), p (quintuple) and multiple (m). High resolution mass spectra analyses (HRMS ESI) were performed using an Orbitrap Elite spectrometer (Thermo Scientific, UK), using electrospray ionization (ESI) in positive and/or negative mode.

Green Metrics

Formulas

Atom Economy (%):

$$AE\ (\%) = \frac{Molar\ Mass\ of\ the\ Product}{Molar\ Mass\ of\ All\ Reactants} \times 100$$

Reaction Mass Efficiency (%):

$$RME\ (\%) = \frac{Mass\ of\ the\ Product}{Total\ Mass\ of\ Reactants} \times 100$$

Process Mass Intensity (reactants, reagents and catalysts):

$$PMI_{RRC} = \frac{mass_{reactants} + mass_{reagents} + mass_{catalysts}}{mass_{product}}$$

Calculations

a) Zhao *et al.*

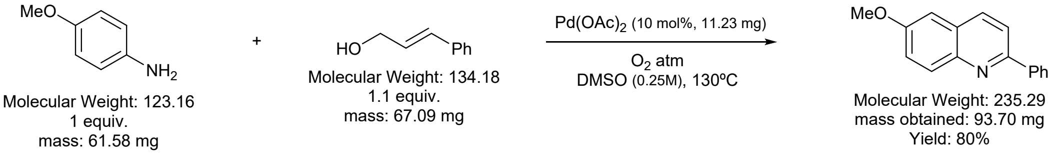

$$AE\ (\%) = \frac{235.29}{123.16 + 134.18} \times 100 = 91\%$$

$$RME\ (\%) = \frac{93.70}{61.58 + 67.09} \times 100 = 73\%$$

$$PMI_{RRC} = \frac{61.58 + 67.09 + 11.23}{93.70} = 1.5$$

b) Chen *et al.*

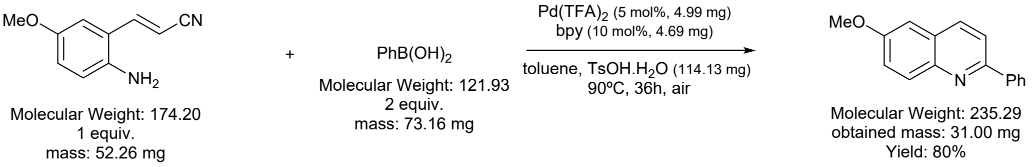

$$AE\ (\%) = \frac{235.29}{174.20 + 121.93} \times 100 = 79\%$$

$$RME\ (\%) = \frac{31.00}{52.26 + 73.16} \times 100 = 25\%$$

$$PMI_{RRC} = \frac{52.26 + 73.16 + 114.13 + 4.99 + 4.69}{31.00} = 8.0$$

c) Jiang *et al.*

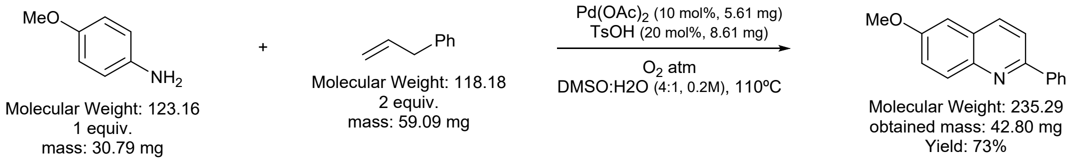

$$AE\ (\%) = \frac{235.29}{123.16 + 118.18} \times 100 = 97\%$$

$$RME\ (\%) = \frac{42.80}{30.79 + 59.09} \times 100 = 48\%$$

$$PMI_{RRC} = \frac{30.79 + 59.09 + 5.61 + 8.61}{42.80} = 2.4$$

d) Our work*:*

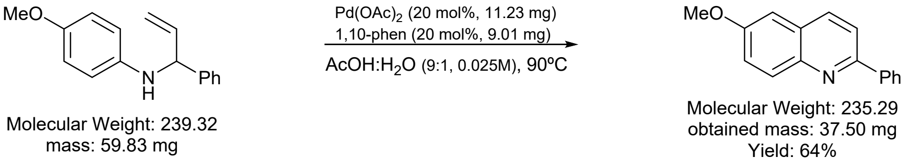

$$AE\ (\%) = \frac{235.29}{239.32} \times 100 = 98\%$$

$$RME\ (\%) = \frac{37.50}{57.83} \times 100 = 63\%$$

$$PMI_{RRC} = \frac{57.83 + 11.23 + 9.01}{37.50} = 2.1$$

Optimization Studies

Temperature

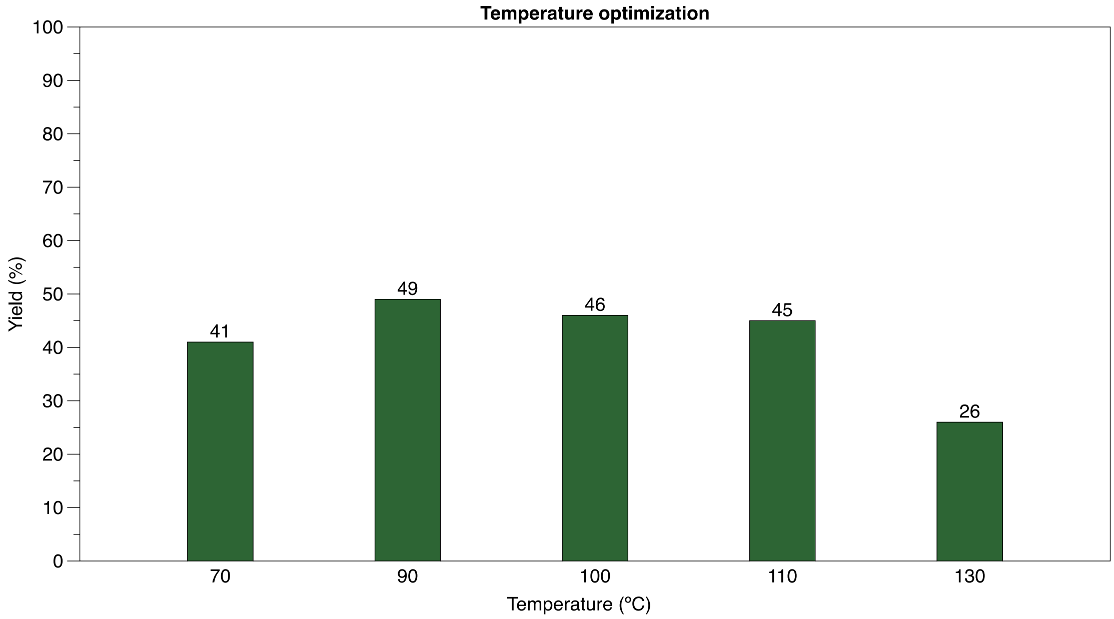

Reaction conditions: 0.1 mmol of starting material, $Pd(OAc)_2$ (10 mol%), 1,10-phen (20 mol%), AcOH (0.1 M), air (1 atm), 24h

Solvent

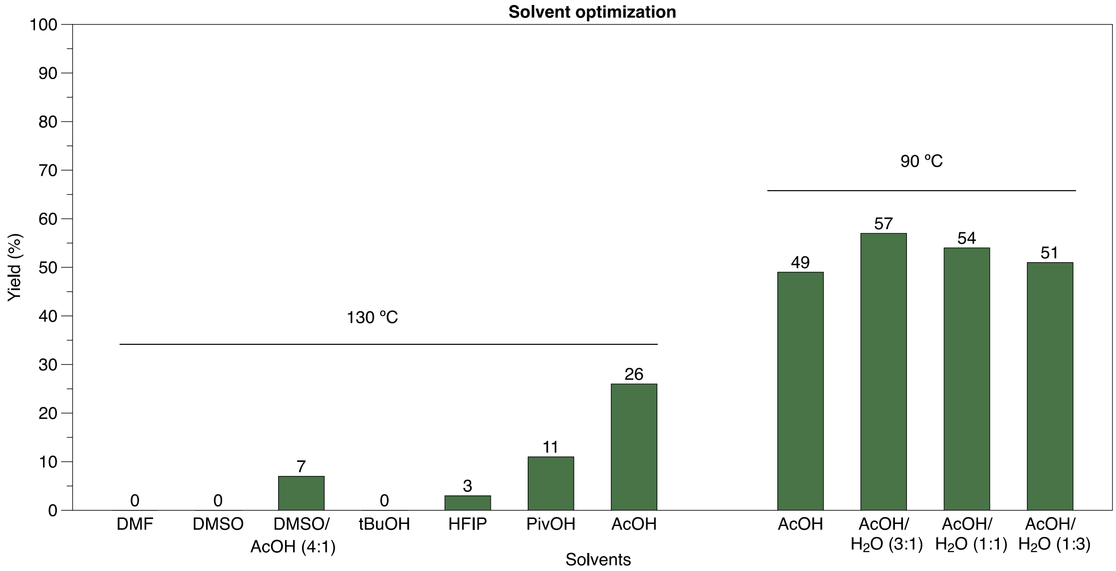

Reaction conditions: 0.1 mmol of starting material, $Pd(OAc)_2$ (10 mol%), 1,10-phen (20 mol%), Solvent (0.1 M), air (1 atm), 24h

Oxidant

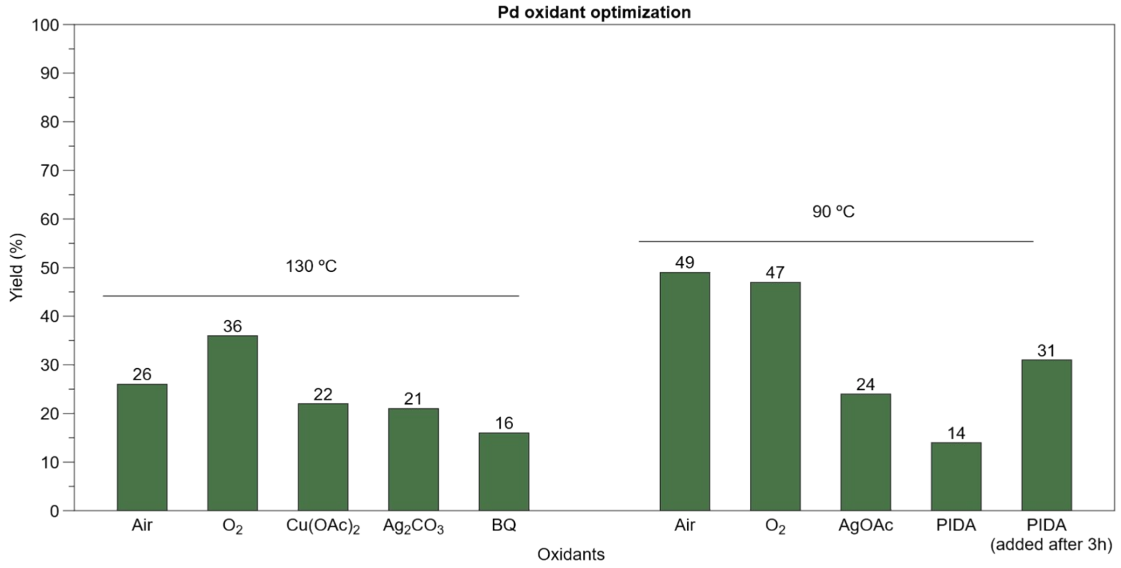

Reaction conditions: 0.1 mmol of starting material, $Pd(OAc)_2$ (10 mol%), 1,10-phen (20 mol%), Solvent (0.1 M), oxidant (1 equiv. or 1 atm), 24h

Time

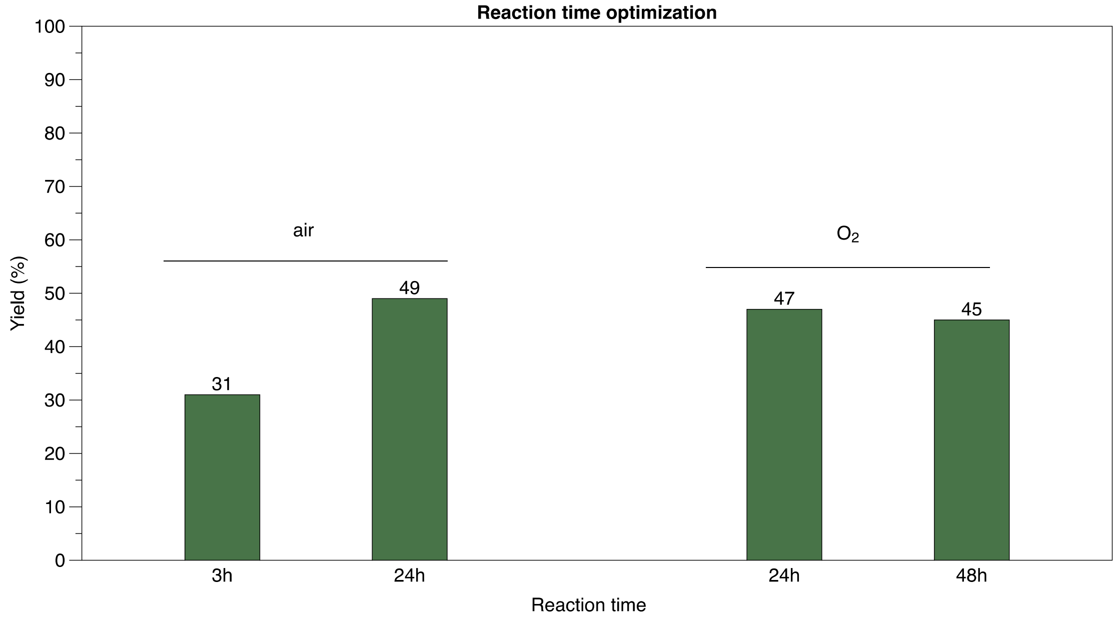

Reaction conditions: 0.1 mmol of starting material, $Pd(OAc)_2$ (10 mol%), 1,10-phen (20 mol%), AcOH (0.1 M), oxidant (1 atm), 90 °C

Palladium Catalyst

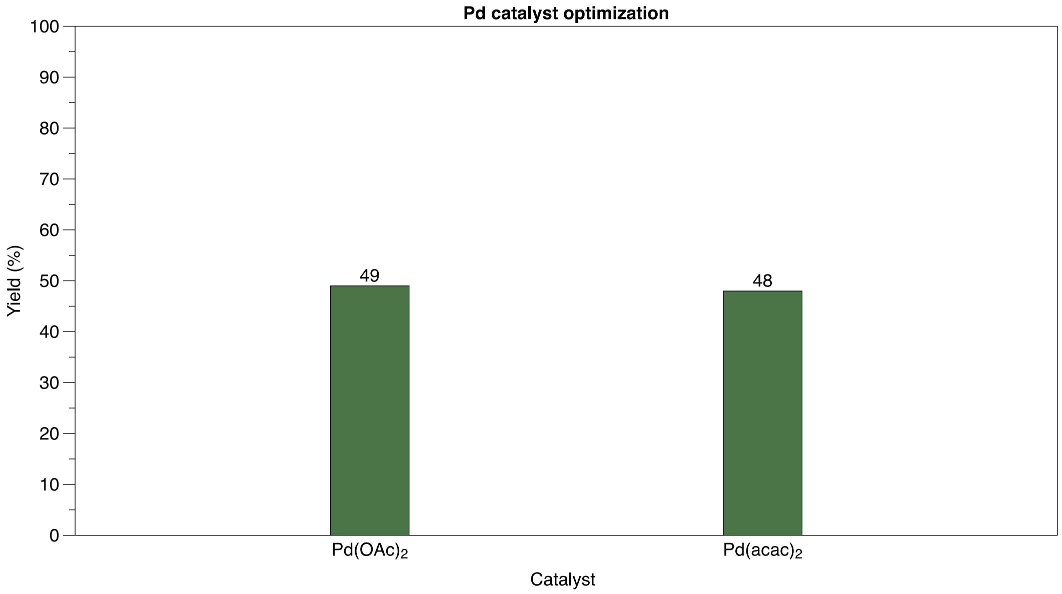

Reaction conditions: 0.1 mmol of starting material, Pd cat (10 mol%), ligand (20 mol%), AcOH (0.1 M), air (1 atm), 90 ºC, 24h

Ligand

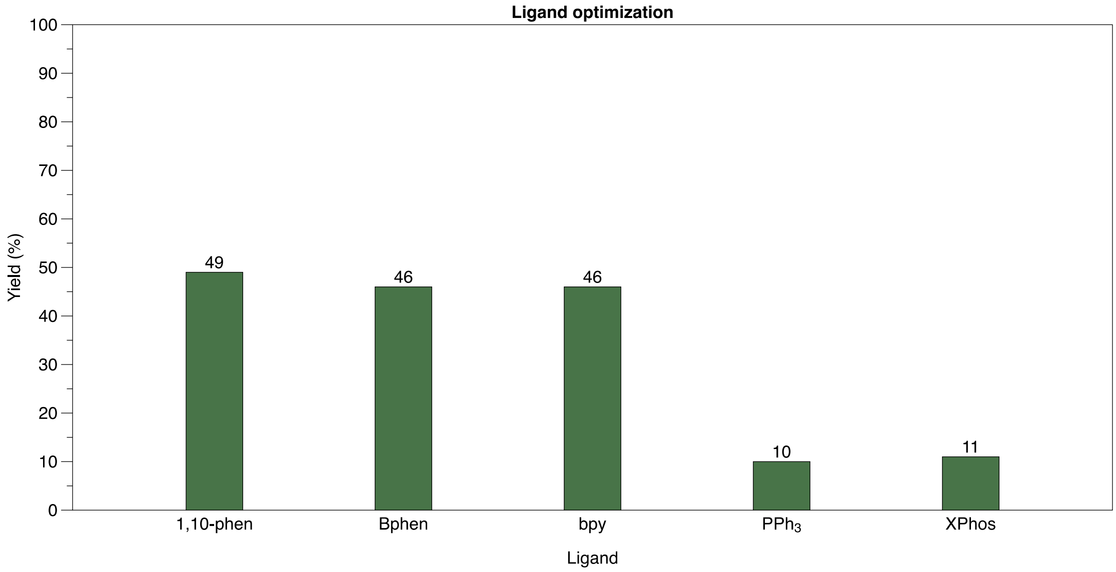

Reaction conditions: 0.1 mmol of starting material, $Pd(OAc)_2$ (10 mol%), ligand (20 mol%), AcOH (0.1 M), air (1 atm), 90 ºC, 24h

Pd/Ligand Loading and Ratio

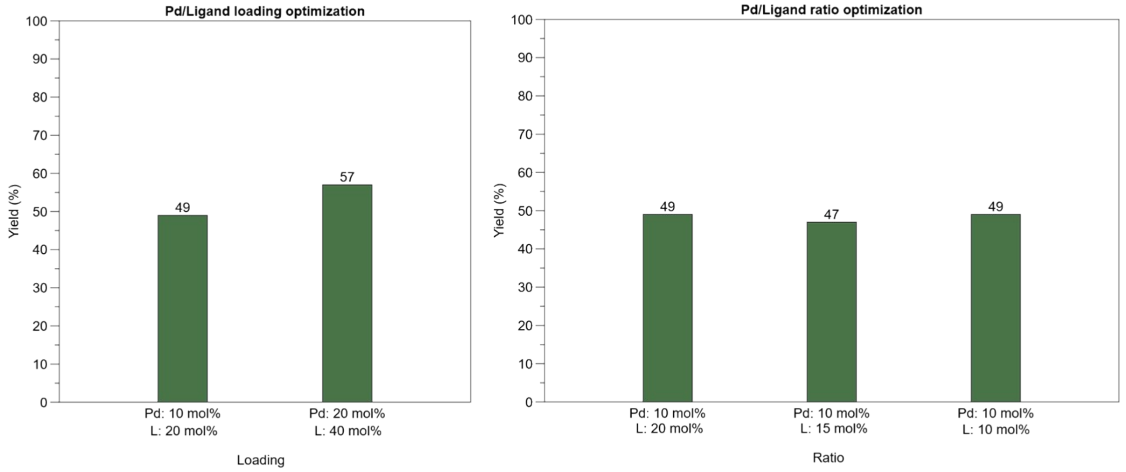

Reaction conditions: 0.1 mmol of starting material, $Pd(OAc)_2$, 1,10-phen, AcOH (0.1 M), air (1 atm), 90 ºC, 24h

Concentration

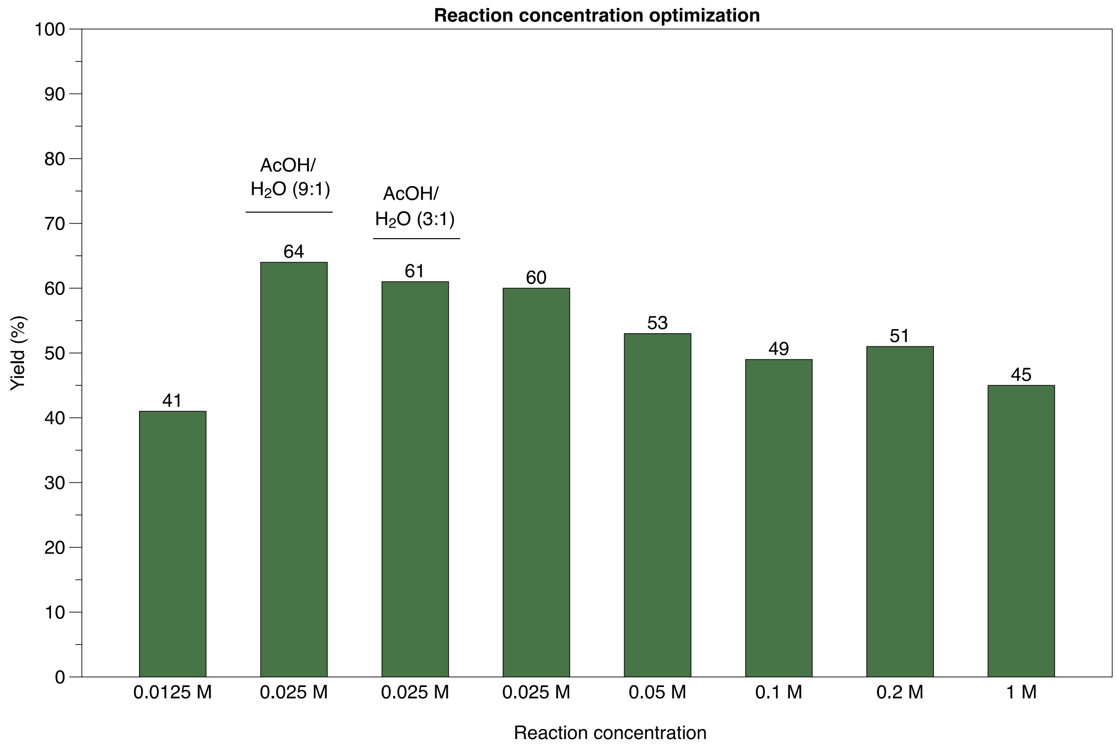

Reaction conditions: 0.1 mmol of starting material, $Pd(OAc)_2$ (10 mol%), 1,10-phen (20 mol%), AcOH, air (1 atm), 90 ºC, 24h

Synthesis of Allylic Amines (Starting Materials)

General Procedure

In a dry round-bottom flask equipped with a magnetic stirring bar and activated 3Å molecular sieves, amine (1 equiv.) and aldehyde (1 equiv.) were dissolved in dry toluene (or dichloromethane). The reaction mixture was stirred overnight at room temperature or under heating. Reaction progress was monitored by thin-layer chromatography (TLC). Upon completion, the reaction mixture was filtered through a Hirsch funnel packed with a thin pad of celite and washed with ethyl acetate. The filtrate was concentrated under reduced pressure to afford the crude imine, which was used directly in the subsequent step without further purification.

In an oven-dried Schlenk tube, equipped with a magnetic stirring bar and 3Å molecular sieves, zinc chloride (0.2-0.3 equiv.) was added. The tube was evacuated under vacuum and gently heated to ensure complete dryness. After cooling under a nitrogen atmosphere, dry tetrahydrofuran (THF) was added and the mixture stirred until complete dissolution of $ZnCl_2$. Separately, in a second dry Schlenk tube equipped with a magnetic stirring bar and 3 Å molecular sieves, vinylmagnesium bromide (or chloride) (2.0 equiv., 0.715–1.5 M in THF) was added under a nitrogen atmosphere. The zinc chloride solution was then transferred dropwise to this mixture and stirred 30 minutes at room temperature. After this time, the previously prepared and dried imine was added to the organozinc solution in dry THF under an inert atmosphere. The reaction mixture was stirred at room temperature overnight. The reaction was then quenched by slow addition of a saturated aqueous solution of NH_4Cl in an ice bath. The mixture was extracted with EtOAc (3x), and the combined organic layers were washed with saturated brine (NaCl), dried over anhydrous sodium sulfate (Na_2SO_4), filtered, and concentrated under reduced pressure. The crude was purified by flash column chromatography on silica gel, yielding the pure allylic amines.

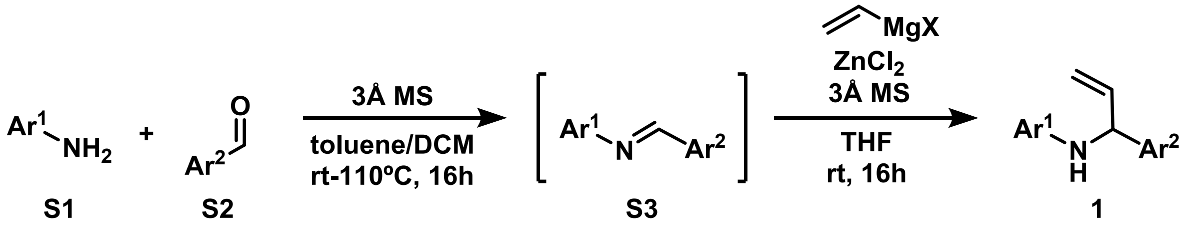

4-methoxy-*N*-(1-phenylallyl)aniline (1a)

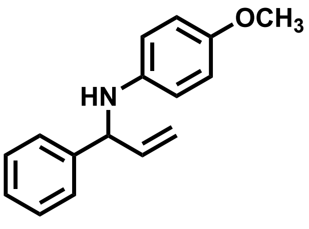

Following the general procedure, *p*-anisidine (S1a) (1.24 g, 10.0 mmol), benzaldehyde (S2a) (1.06 g, 10.0 mmol) and 12 mL of toluene were stirred at room temperature overnight. The resulting imine (S3a) was reacted with $ZnCl_2$ (273.50 mg, 2.0 mmol, 0.2 equiv.) and vinylmagnesium bromide (28.07 mL, 20.0 mmol, 0.715 M in THF) under nitrogen atmosphere at room temperature overnight. After aqueous workup and purification by flash column chromatography (hexane/EtOAc gradient), amine 1a was obtained as an orange oil (1.76 g, 8.3 mmol, 83% yield).

^{1}H NMR (400 MHz, $CDCl_3$): δ 7.45 – 7.33 (m, 4H), 7.33 – 7.27 (m, 1H), 6.82 – 6.72 (m, 2H), 6.64 – 6.55 (m, 2H), 6.06 (ddd, *J* = 17.1, 10.2, 6.0 Hz, 1H), 5.36 – 5.20 (m, 2H), 4.89 (d, *J* = 6.0, 1H), 3.82 (br s, 1H), 3.74 (s, 3H). **^{13}C NMR** (101 MHz, $CDCl_3$): δ 152.3, 142.3, 141.6, 139.6, 128.8, 127.5, 127.2, 116.0, 115.0, 114.8, 61.9, 55.8. **IR (ATR):** 3396, 3063, 3028, 3001, 2936, 2908, 2831, 1639, 1616, 1604, 1507, 1453, 1407, 1228, 1178, 1035, 923, 818, 764, 698. **HRMS (ESI$^+$ / [M+H]$^+$):** calculated m/z ($[C_{16}H_{18}NO]^+$): 240.1383, found 240.1373.

***N*-(1-phenylallyl)aniline (1b)**

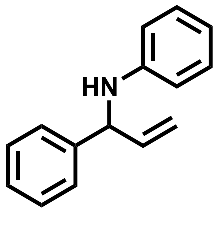

Following the general procedure, aniline (S1b) (150 mg, 1.61 mmol), benzaldehyde (S2a) (170.93 mg, 1.61 mmol) and 1.8 mL of toluene were stirred at 40ºC overnight. The resulting imine (S3b) was dissolved in dry THF (4 mL) and reacted with $ZnCl_2$ (65.85 mg, 0.48 mmol, 0.3 equiv.) and vinylmagnesium bromide (4.51 mL, 3.22 mmol, 0.715 M in THF) under nitrogen atmosphere at room temperature overnight. After aqueous workup and purification by flash column chromatography (hexane/EtOAc gradient), amine 1b was obtained as a light-yellow oil (124.0 mg, 0.59 mmol, 37% yield).

^{1}H NMR (400 MHz, $CDCl_3$): δ 7.43 – 7.33 (m, 4H), 7.32 – 7.25 (m, 1H), 7.20 – 7.11 (m, 2H), 6.71 (tt, *J* = 7.3, 1.1 Hz, 1H), 6.61(d, *J* = 7.6 Hz, 2H), 6.05 (ddd, *J* = 17.1, 10.3, 5.9 Hz, 1H), 5.34 – 5.20 (m, 2H), 4.95 (d, *J* = 5.9 Hz, 1H), 4.05 (s, 1H). **^{13}C NMR** (101 MHz, $CDCl_3$): δ 147.3, 142.0, 139.2, 129.2, 128.9, 127.6, 127.3, 117.7, 116.2, 113.7, 61.0. **IR (ATR):** 3408, 3083, 3052, 3025, 2978, 2924, 2850, 1639, 1600, 1499, 1453, 1430, 1314,

1267, 1244, 992, 926, 748, 690. **HRMS (ESI$^+$ / [M+H]$^+$):** calculated m/z ([$C_{15}H_{16}N$]$^+$): 210.1278, found 210.1274.

4-chloro-*N*-(1-phenylallyl)aniline (1c)

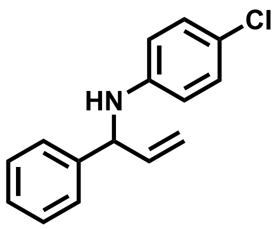

Following the general procedure, 4-chloroaniline (S1c) (150 mg, 1.18 mmol), benzaldehyde (S2a) (124.78 mg, 1.18 mmol) and 1.8 mL of toluene were stirred at 40ºC overnight. The resulting imine (S3c) was dissolved in dry THF (4 mL) and reacted with $ZnCl_2$ (48.08 mg, 0.35 mmol, 0.3 equiv.) and vinylmagnesium bromide (3.29 mL, 2.35 mmol, 0.715 M in THF) under nitrogen atmosphere at room temperature overnight. After aqueous workup and purification by flash column chromatography (hexane/toluene gradient), amine 1c was obtained as a light-yellow oil (163.1 mg, 0.67 mmol, 57% yield).

^{1}H NMR (400 MHz, $CDCl_3$): δ 7.40 – 7.33 (m, 4H), 7.32 – 7.26 (m, 1H), 7.12 – 7.04 (m, 2H), 6.55 – 6.48 (m, 2H), 6.02 (ddd, *J* = 17.1, 10.2, 5.8 Hz, 1H), 5.31 – 5.21 (m, 2H), 4.90 (dt, *J* = 5.9, 1.4 Hz, 1H), 4.06 (br s, 1H). **^{13}C NMR** (101 MHz, $CDCl_3$): δ 145.8, 141.5, 138.8, 129.1, 129.0, 127.8, 127.2, 122.3, 116.4, 114.8, 61.0. **IR (ATR):** 3416, 3087, 3059, 3028, 2978, 2923, 2854, 1643, 1596, 1492, 1453, 1399, 1314, 1294, 1267, 1240, 1178, 1089, 923, 810, 698. **HRMS (ESI$^+$ / [M+H]$^+$):** calculated m/z ([$C_{15}H_{15}ClN$]$^+$): 244.0888, found 244.0883.

***N*-(1-phenylallyl)-3-(trifluoromethyl)aniline (1d)**

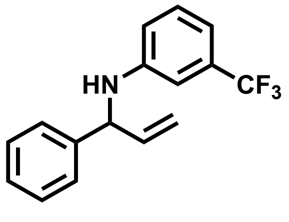

Following the general procedure, 3-(trifluoromethyl)aniline (S1d) (150 mg, 0.93 mmol), benzaldehyde (S2a) (98.8 mg, 0.93 mmol) and 1.8 mL of toluene were stirred at 60ºC overnight. The resulting imine (S3d) was dissolved in dry THF (4 mL) and reacted with $ZnCl_2$ (38.1 mg, 0.28 mmol, 0.3 equiv.) and vinylmagnesium bromide (2.60 mL, 1.86 mmol, 0.715 M in THF) under nitrogen atmosphere at room temperature overnight. After aqueous workup and purification by flash column chromatography (hexane/EtOAc gradient), amine 1d was obtained as a light-yellow oil (142.3 mg, 0.51 mmol, 55% yield).

^{1}H NMR (400 MHz, $CDCl_3$): δ 7.42-7.34 (m, 4H), 7.33-7.27 (m, 1H), 7.21 (t, *J* = 8.0 Hz, 1H), 6.93 (dt, *J* = 7.7, 0.8 Hz, 1H), 6.83 (t, J = 2.2 Hz, 1H), 6.72 (dd, *J* = 8.2, 2.6 Hz, 1H), 6.04 (ddd, *J* = 17.1, 10.2, 5.7 Hz, 1H), 5.34-5.23 (m, 2H), 4.96 (d, *J* = 5.7 Hz, 1H), 4.24 (br s, 1H). **^{13}C NMR** (101 MHz, $CDCl_3$): δ 147.4, 141.2, 138.4, 131.5 (q, *J* = 31.7 Hz), 129.7, 129.0, 127.9, 127.3, 124.5 (q, *J* = 272.7 Hz), 116.6, 116.4, 114.2 (q, *J* = 4.0 Hz), 110.1 (q, *J* = 4.0 Hz), 60.8. **IR (ATR):** 3419, 3090, 3063, 3032, 2986, 2928, 2854, 1616, 1596, 1511, 1492, 1437, 1341, 1283, 1163, 1120, 1070, 992, 783, 698. **HRMS (ESI$^+$ / [M+H]$^+$):** calculated m/z ($[C_{16}H_{15}F_3N]^+$): 278.1152, found 278.1144.

3,4,5-trimethoxy-*N*-(1-phenylallyl)aniline (1e)

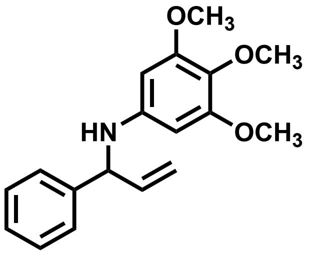

Following the general procedure, 3,4,5-trimethoxyaniline (S1e) (300 mg, 1.64 mmol), benzaldehyde (S2a) (173.78 mg, 1.64 mmol) and 1.8 mL of toluene were stirred at 90ºC overnight. The resulting imine (S3e) was dissolved in dry THF (6 mL) and reacted with $ZnCl_2$ (55.75 mg, 0.41 mmol, 0.25 equiv.) and vinylmagnesium chloride (2.18 mL, 3.27 mmol, 1.5 M in THF) under nitrogen atmosphere at room temperature overnight. After aqueous workup and purification by flash column chromatography (hexane/EtOAc gradient), amine 1e was obtained as a light-brown oil (317.7 mg, 1.07 mmol, 65% yield).

^{1}H NMR (400 MHz, $CDCl_3$): δ 7.43 – 7.32 (m, 4H), 7.31 – 7.25 (m, 1H), 6.05 (ddd, *J* = 17.1, 10.3, 6.1 Hz, 1H), 5.85 (s, 2H), 5.35 – 5.20 (m, 2H), 4.88 (d, *J* = 6.1, 1H), 3.98 (br s, 1H), 3.77-3.71 (m, 9H). **^{13}C NMR** (101 MHz, $CDCl_3$): δ 153.8, 144.1, 142.1, 139.4, 130.3, 128.9, 127.6, 127.2, 116.2, 91.4, 61.7, 61.2, 55.9. **IR (ATR):** 3376, 3084, 3060, 3028, 3000, 2959, 2936, 2908, 2840, 2827, 1608, 1508, 1451, 1409, 1233, 1206, 1186, 1124, 1006, 923, 733, 700. **HRMS (ESI$^+$ / [M+H]$^+$):** calculated m/z ($[C_{18}H_{22}NO_3]^+$): 300.1595, found 300.1588.

4-(methylthio)-*N*-(1-phenylallyl)aniline (1f)

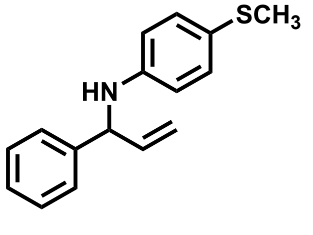

Following the general procedure, 4-(methylthio)aniline (S1f) (300 mg, 2.15 mmol), benzaldehyde (S2a) (228.69 mg, 2.15 mmol) and 1.8 mL of toluene were stirred at 90ºC

overnight. The resulting imine (S3f) was dissolved in dry THF (6 mL) and reacted with $ZnCl_2$ (88.10 mg, 0.65 mmol, 0.3 equiv.) and vinylmagnesium chloride (3.59 mL, 4.31 mmol, 1.2 M in THF) under nitrogen atmosphere at room temperature overnight. After aqueous workup and purification by flash column chromatography (hexane/EtOAc gradient), amine 1f was obtained as a yellow oil (201.0 mg, 0.79 mmol, 37% yield).

^{1}H NMR (400 MHz, $CDCl_3$): δ 7.40 – 7.33 (m, 4H), 7.32-7.26 (m, 1H), 7.21 – 7.15 (m, 2H), 6.59 – 6.52 (m, 2H), 6.04 (ddd, *J* = 17.1, 10.3, 5.9 Hz, 1H), 5.33 – 5.20 (m, 2H), 4.92 (d, *J* = 5.9 Hz, 1H), 4.08 (s, 1H), 2.40 (s, 3H). **^{13}C NMR** (101 MHz, $CDCl_3$): δ 146.1, 141.7, 138.9, 131.3, 128.9, 127.7, 127.2, 124.7, 116.3, 114.3, 61.0, 19.1. **IR (ATR):** 3404, 3082, 3060, 3026, 2980, 2917, 2852, 2831, 1596, 1496, 1451, 1311, 1288, 1239, 1182, 924, 813, 757, 699, 513. **HRMS (ESI$^+$ / [M+H]$^+$):** calculated m/z ($[C_{16}H_{18}NS]^+$): 256.1155, found 256.1144.

N-(1-phenylallyl)-[1,1'-biphenyl]-4-amine (1g)

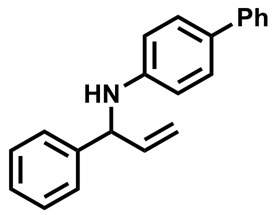

Following the general procedure, [1,1'-biphenyl]-4-amine (S1g) (425 mg, 2.51 mmol), benzaldehyde (S2a) (266.50 mg, 2.51 mmol) and 1.8 mL of toluene were stirred at 90ºC overnight. The resulting imine (S3g) was dissolved in dry THF (6 mL) and reacted with $ZnCl_2$ (102.68 mg, 0.75 mmol, 0.3 equiv.) and vinylmagnesium chloride (4.19 mL, 5.03 mmol, 1.2 M in THF) under nitrogen atmosphere at room temperature overnight. After aqueous workup and purification by flash column chromatography (hexane/EtOAc gradient), amine 1g was obtained as a yellow oil (335.3 mg, 1.18 mmol, 47% yield).

^{1}H NMR (400 MHz, $CDCl_3$): δ 7.57 – 7.51 (m, 2H), 7.45 – 7.36 (m, 8H), 7.34 – 7.23 (m, 2H), 6.73 – 6.66 (m, 2H), 6.09 (ddd, *J* = 17.2, 10.2, 5.9 Hz, 1H), 5.37 – 5.25 (m, 2H), 5.01 (d, *J* = 5.8 Hz, 1H), 4.16 (s, 1H). **IR (ATR):** 3409, 3080, 3058, 3027, 3004, 2982, 2960, 2937, 2904, 2837, 1610, 1599, 1523, 1488, 1452, 1443, 1320, 1297, 1272, 1265, 1204, 1150, 1067, 925, 823, 761, 736, 697. **HRMS (ESI$^+$ / [M+H]$^+$):** calculated m/z ($[C_{21}H_{20}N]^+$): 286.1591, found 286.1575.

N-(1-phenylallyl)benzo[*d*][1,3]dioxol-5-amine (1h)

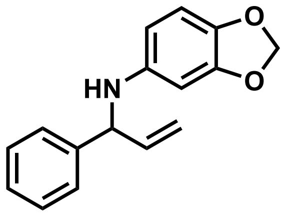

Following the general procedure, benzo[*d*][1,3]dioxol-5-amine (S1i) (150 mg, 1.09 mmol), benzaldehyde (S2a) (116.08 mg, 1.09 mmol) and 1.8 mL of toluene were stirred at 50ºC overnight. The resulting imine (S3h) was dissolved in dry THF (6 mL) and reacted with $ZnCl_2$ (44.72 mg, 0.33 mmol, 0.3 equiv.) and vinylmagnesium bromide (3.06 mL, 2.19 mmol, 0.715 M in THF) under nitrogen atmosphere at room temperature overnight. After aqueous workup and purification by flash column chromatography (hexane/EtOAc gradient), amine 1h was obtained as a dark brown powder (151.2 mg, 0.60 mmol, 55% yield).

^{1}H NMR (400 MHz, $CDCl_3$): δ 7.41 – 7.32 (m, 4H), 7.31 – 7.26 (m, 1H), 6.61 (d, J = 8.3 Hz, 1H), 6.24 (d, J = 2.4 Hz, 1H), 6.09 – 5.96 (m, 2H), 5.88-5-79 (m, 2H), 5.32 – 5.18 (m, 2H), 4.84 (d, J = 6.0 Hz, 1H), 3.85 (br s, 1H). **^{13}C NMR** (101 MHz, $CDCl_3$): δ 148.3, 143.1, 142.0, 139.8, 139.4, 128.9, 127.6, 127.2, 116.1, 108.6, 105.5, 100.7, 96.8, 61.9. **IR (ATR):** 3404, 3063, 3028, 3005, 2970, 2877, 2773, 1635, 1612, 1503, 1488, 1453, 1298, 1232, 1201, 1039, 926, 702. **HRMS (ESI$^+$ / [M+H]$^+$):** calculated m/z ([$C_{16}H_{16}NO_2$]$^+$): 254.1176, found 254.1168.

***N*-(1-phenylallyl)naphthalen-2-amine (1i)**

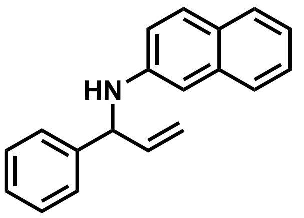

Following the general procedure, naphthalen-2-amine (S1j) (200 mg, 1.40 mmol), benzaldehyde (S2a) (148.57 mg, 1.40 mmol) and 3 mL of toluene were stirred at 60ºC overnight. The resulting imine (S3i) was dissolved in dry THF (6 mL) and reacted with $ZnCl_2$ (57.24 mg, 0.42 mmol, 0.3 equiv.) and vinylmagnesium chloride (3.42 mL, 2.80 mmol, 0.818 M in THF) under nitrogen atmosphere at room temperature overnight. After aqueous workup and purification by flash column chromatography (hexane/EtOAc gradient), amine 1i was obtained as an orange oil (188.3 mg, 0.73 mmol, 52% yield).

^{1}H NMR (400 MHz, $CDCl_3$): δ 7.92 – 7.85 (m, 1H), 7.83 – 7.76 (m, 1H), 7.52 – 7.42 (m, 4H), 7.41 – 7.33 (m, 2H), 7.32 – 7.27 (m, 1H), 7.26 – 7.21 (m, 2H), 6.54 (dd, *J* = 6.7, 2.0 Hz, 1H), 6.16 (ddd, *J* = 17.1, 10.2, 5.9 Hz, 1H), 5.41 – 5.25 (m, 2H), 5.13 (d, *J* = 6.0 Hz, 1H), 4.74 (br s, 1H). **^{13}C NMR** (101 MHz, $CDCl_3$): δ 142.2, 141.8, 139.2, 134.4, 129.0, 128.9, 127.7, 127.3, 126.6, 125.8, 124.9, 123.6, 120.0, 117.8, 116.5, 106.4, 61.1. **IR (ATR):** 3439, 3396, 3059, 3028, 2982, 2850, 1623, 1581, 1523, 1472, 1407, 1345, 1283, 973, 926, 783, 768, 752, 698. **HRMS (ESI$^+$ / [M+H]$^+$):** calculated m/z ($[C_{19}H_{18}N]^+$): 260.1434, found 260.1428.

6-methoxy-*N*-(1-phenylallyl)pyridin-3-amine (1j)

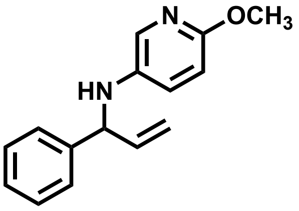

Following the general procedure, 5-amino-2-methoxypyridine (S1h) (300 mg, 2.42 mmol), benzaldehyde (S2a) (256.81 mg, 2.42 mmol) and 3.5 mL of toluene were stirred at 60ºC overnight. The resulting imine (S3j) was dissolved in dry THF (6 mL) and reacted with $ZnCl_2$ (65.85 mg, 0.48 mmol, 0.3 equiv.) and vinylmagnesium bromide (6.80 mL, 4.83 mmol, 0.715 M in THF) under nitrogen atmosphere at room temperature overnight. After aqueous workup and purification by flash column chromatography (hexane/EtOAc gradient), amine 1j was obtained as a greenish-yellow oil (121.4 mg, 0.51 mmol, 21% yield).

^{1}H NMR (400 MHz, $CDCl_3$): δ 7.54 (d, *J* = 2.8, 1H), 7.39 – 7.32 (m, 4H), 7.30 – 7.26 (m, 1H), 6.96 (dd, *J* = 8.8, 2.9 Hz, 1H), 6.57 (d, *J* = 8.8 Hz, 1H), 6.02 (ddd, *J* = 17.1, 10.2, 6.1 Hz, 1H), 5.31 – 5.19 (m, 2H), 4.83 (d, *J* = 6.0 Hz, 1H), 3.83 (s, 3H). *The labile N–H proton signal seems to overlap with the OCH_3 protons resonance, making the precise determination of its chemical shift not possible.* **^{13}C NMR** (101 MHz, $CDCl_3$): δ 152.3, 149.3, 148.4, 141.7, 139.6, 134.9, 119.3, 115.8, 115.0, 114.8, 111.3, 110.4, 61.7, 56.0, 56.0, 55.9. **IR (ATR):** 3381, 3059, 3025, 3009, 2974, 2943, 2901, 2843, 1581, 1492, 1372, 1259, 1236, 1031, 923, 822, 698. **HRMS (ESI$^+$ / [M+H]$^+$):** calculated m/z ($[C_{15}H_{17}N_2O]^+$): 241.1336, found 241.1331.

***N*-(1-(furan-2-yl)allyl)-4-methoxyaniline (1k)**

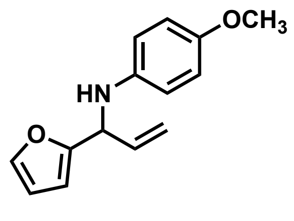

Following the general procedure, *p*-anisidine (S1a) (150 mg, 1.22 mmol), furfural (S2b) (117.03 mg, 1.22 mmol) and 1.8 mL of toluene were stirred at 50ºC overnight. The resulting imine (S3k) was dissolved in dry THF (6 mL) and reacted with $ZnCl_2$ (49.80 mg, 0.37 mmol, 0.3 equiv.) and vinylmagnesium bromide (3.41 mL, 2.44 mmol, 0.715 M in THF) under nitrogen atmosphere at room temperature overnight. After aqueous workup and purification by flash column chromatography (hexane/EtOAc gradient), amine 1k was obtained as a orange oil (204.7 mg, 0.89 mmol, 73% yield).

^{1}H NMR (500 MHz, $CDCl_3$) δ 7.38 (dd, *J* = 1.8, 0.9 Hz, 1H), 6.80 – 6.72 (m, 2H), 6.66 – 6.59 (m, 2H), 6.32 (dd, *J* = 3.2, 1.8 Hz, 1H), 6.21 (dt, *J* = 3.2, 0.8 Hz, 1H), 6.04 (ddd, *J* = 17.2, 10.2, 5.8 Hz, 1H), 5.36 – 5.23 (m, 2H), 4.99 (d, *J* = 5.7 Hz, 1H), 3.74 (s, 3H). *The labile N–H proton signal seems to overlap with the OCH_3 protons resonance, making the precise determination of its chemical shift not possible.* **^{13}C NMR** (125 MHz, $CDCl_3$): δ 154.7, 152.7, 142.2, 141.0, 136.8, 117.0, 115.4, 114.8, 110.4, 106.8, 55.8, 55.7. **IR (ATR):** 3392, 9117, 2997, 2951, 2936, 2908, 2835, 1643, 1619, 1592, 1511, 1465, 1441, 1407, 1232, 1178, 1147, 1039, 1012, 930, 818, 733. **HRMS (ESI$^+$ / [M+H]$^+$):** calculated m/z ($[C_{14}H_{16}NO_2]^+$): 230.1176, found 230.1168.

4-methoxy-*N*-(1-(1-methyl-1*H*-imidazol-2-yl)allyl)aniline (1l)

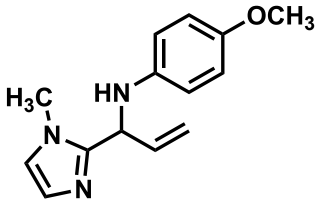

Following the general procedure, *p*-anisidine (S1a) (150 mg, 1.22 mmol), 1-methyl-1*H*-imidazole-2-carbaldehyde (S2c) (134.12 mg, 1.22 mmol) and 1.8 mL of toluene were stirred at 50ºC overnight. The resulting imine (S3l) was dissolved in dry THF (6 mL) and reacted with $ZnCl_2$ (49.80 mg, 0.37 mmol, 0.3 equiv.) and vinylmagnesium bromide (2.98 mL, 2.44 mmol, 0.818 M in THF) under nitrogen atmosphere at room temperature overnight. After aqueous workup and purification by flash column chromatography (hexane/EtOAc gradient), amine 1l was obtained as a pale brown powder (184.8 mg, 0.76 mmol, 62% yield).

^{1}H NMR (400 MHz, $CDCl_3$): δ 6.98 (d, *J* = 1.2 Hz, 1H), 6.82 (d, *J* = 1.1 Hz, 1H), 6.79 – 6.73 (m, 2H), 6.69 – 6.62 (m, 2H), 6.00 (ddd, *J* = 16.9, 10.3, 6.5 Hz, 1H), 5.31 – 5.21 (m, 2H), 5.03 (d, *J* = 6.5 Hz, 1H), 4.33 (br s, 1H), 3.73 (s, 3H), 3.64 (s, 3H). **^{13}C NMR** (101 MHz, $CDCl_3$): δ 152.6, 147.0, 140.7, 136.4, 127.5, 121.6, 117.1, 115.4, 114.9, 55.8, 54.7, 32.9. **IR (ATR):** 3373, 3106, 2997, 2951, 2835, 1643, 1616, 1511, 1465, 1407, 1279,

1232, 1178, 1035, 822, 748. **HRMS (ESI$^+$ / [M+H]$^+$):** calculated m/z ([$C_{14}H_{18}N_3O$]$^+$): 244.1445, found 244.144.

***N*-(1-(3,4-dimethoxyphenyl)allyl)-4-methoxyaniline (1m)**

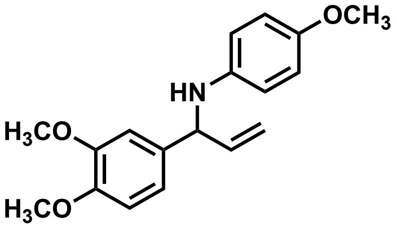

Following the general procedure, *p*-anisidine (S1a) (150 mg, 1.22 mmol), 3,4-dimethoxybenzaldehyde (S2d) (202.40 mg, 1.22 mmol) and 1.8 mL of toluene were stirred at 55ºC overnight. The resulting imine (S3m) was dissolved in dry THF (6 mL) and reacted with $ZnCl_2$ (49.80 mg, 0.37 mmol, 0.3 equiv.) and vinylmagnesium bromide (3.41 mL, 2.44 mmol, 0.715 M in THF) under nitrogen atmosphere at room temperature overnight. After aqueous workup and purification by flash column chromatography (hexane/EtOAc gradient), amine 1m was obtained as an orange oil (176.3 mg, 0.59 mmol, 48% yield).

^{1}H NMR (500 MHz, $CDCl_3$): δ 6.94-6.89 (m, 2H), 6.83 (d, *J* = 8.4 Hz, 1H), 6.76 – 6.71 (m, 2H), 6.63 – 6.57 (m, 2H), 6.04 (ddd, *J* = 17.2, 10.2, 6.1 Hz, 1H), 5.35 – 5.14 (m, 2H), 4.78 (d, *J* = 6.0 Hz, 1H), 4.33 (br s, 1H), 3.87 (s, 6H), 3.72 (s, 3H). *The labile N–H proton signal seems to overlap with the OCH_3 protons resonance (3.87 ppm), making the precise determination of its chemical shift not possible.* **^{13}C NMR** (101 MHz, $CDCl_3$): δ 152.6, 147.0, 140.7, 136.4, 127.5, 121.6, 117.1, 115.4, 114.9, 55.8, 54.7, 32.9. **IR (ATR):** 3388, 3067, 3001, 2955, 2936, 2905, 2835, 1639, 1592, 1507, 1461, 1441, 1418, 1228, 1139, 1027, 923, 814, 760.

Synthesis of Quinolines

General Procedure

A sealed tube or screw-cap Schlenk tube, equipped with a magnetic stirring bar, was charged with $Pd(OAc)_2$ (20 mol%) and 1,10-phenanthroline (20 mol%). Then, allylic amine (0.25 mmol) was dissolved in acetic acid:water (10 ml, 9:1) and added to the reaction vessel (washed several times). The reaction mixture was stirring at 90ºC, for 24h, under an air atmosphere. After cooling to room temperature, the mixture was neutralized with a saturated sodium bicarbonate aqueous solution and extracted with ethyl acetate (3-5 times). The combined organic layers were dried over Na_2SO_4, filtered and concentrated. The crude was purified by silica gel flash column chromatography (hexane/EtOAc) to afford the desired quinoline derivative.

6-methoxy-2-phenylquinoline (2a)

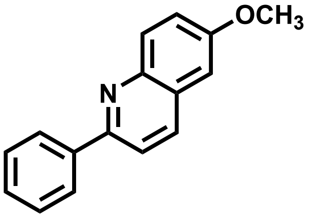

Yield: 64% (37.4 mg, 0.16 mmol). **Appearance:** Off-white solid. **^{1}H NMR** (400 MHz, $CDCl_3$): δ 8.19-8.05 (m, 4H), 7.83 (d, *J* = 8.5 Hz, 1H), 7.52 (t, *J* = 7.4 Hz, 2H), 7.44 (t, *J* = 7.3 Hz, 1H), 7.39 (dd, *J* = 9.2, 2.8 Hz, 1H), 7.09 (d, *J* = 2.8 Hz, 1H), 3.94 (s, 3H). **^{13}C NMR** (101 MHz, $CDCl_3$): δ 157.8, 155.2, 144.5, 139.9, 135.6, 131.3, 129.1, 128.9, 128.3, 127.4, 122.5, 119.4, 105.1, 55.7. **IR (ATR):** 3060, 3005, 1619, 1596, 1556, 1490, 1454, 1377, 1339, 1322, 1242, 1226, 1162, 1020, 831, 762, 700, 470. **HRMS (ESI$^+$ / [M+H]$^+$):** calculated m/z ($[C_{16}H_{14}NO]^+$): 236.107, found 236.1064.

2-phenylquinoline (2b)

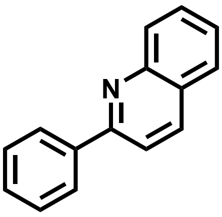

Yield: 46% (23.7 mg, 0.12 mmol). **Appearance:** Off-white solid. **^{1}H NMR** (400 MHz, $CDCl_3$): δ 8.33 – 8.11 (m, 4H), 7.88 (d, *J* = 8.6 Hz, 1H), 7.83 (d, *J* = 8.2 Hz, 1H), 7.74 (ddd, *J* = 8.4, 6.8, 1.5 Hz, 1H), 7.63 – 7.41 (m, 4H). **^{13}C NMR** (101 MHz, $CDCl_3$): δ

157.5, 148.4, 139.8, 136.9, 129.9, 129.8, 129.4, 129.0, 127.7, 127.6, 127.3, 126.4, 119.1. **IR (ATR, neat):** 3057, 2958, 2924, 2853, 1732, 1616, 1596, 1553, 1508, 1490, 1446, 1423, 1318, 1283, 1270, 1242, 1127, 1025, 829, 770, 690, 676. **HRMS (ESI$^+$ / [M+H]$^+$):** calculated m/z ([$C_{15}H_{12}N$]$^+$): 206.0965, found 206.0962.

6-chloro-2-phenylquinoline (2c)

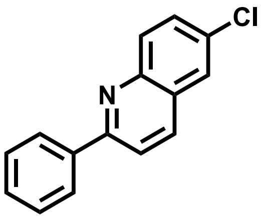

Yield: 38% (22.9 mg, 0.10 mmol). **Appearance:** Light-yellow solid. **^{1}H NMR** (400 MHz, $CDCl_3$): δ 8.25 – 8.05 (m, 4H), 7.89 (d, *J* = 8.7 Hz, 1H), 7.80 (d, *J* = 2.4 Hz, 1H), 7.66 (dd, *J* = 9.0, 2.4 Hz, 1H), 7.60 – 7.43 (m, 3H). **^{13}C NMR** (101 MHz, $CDCl_3$): δ 157.7, 146.8, 139.3, 136.0, 132.0, 131.5, 130.7, 129.7, 129.0, 127.8, 127.6, 126.3, 119.9. **IR (ATR):** 3066, 3059, 3034, 2963, 2926, 2855, 1596, 1582, 1549, 1485, 1446, 1320, 1193, 1075, 886, 876, 832, 783, 755, 695. **HRMS (ESI$^+$ / [M+H]$^+$):** calculated m/z ([$C_{15}H_{11}ClN$]$^+$): 240.0575, found 240.0571.

2-phenyl-7-(trifluoromethyl)quinoline (2d)

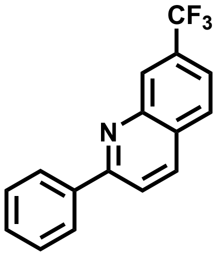

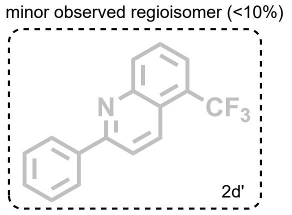

Yield: 11% (7.7 mg, 0.03 mmol). **Appearance:** white solid. **^{1}H NMR** (400 MHz, $CDCl_3$): δ 8.49 (s, 1H), 8.27 (d, *J* = 8.7 Hz, 1H), 8.22 – 8.15 (m, 2H), 8.00 (d, *J* = 8.7 Hz, 1H), 7.94 (d, *J* = 8.6 Hz, 1H), 7.70 (dd, *J* = 8.6, 2.0 Hz, 1H), 7.60 – 7.47 (m, 3H). **^{13}C NMR** (101 MHz, $CDCl_3$): δ 158.8, 147.5, 139.0, 136.8, 131.6 (q, $^{2}J_{C\text{-}F}$ = 32.7), 130.0, 129.1, 128.8, 128.7 (q, $^{4}J_{C\text{-}F}$ = 1.1 Hz), 127.7, 127.7 (q, $^{3}J_{C\text{-}F}$ = 4.6 Hz), 124.2 (q, $^{1}J_{C\text{-}F}$ = 272.7 Hz), 122.0 (q, $^{3}J_{C\text{-}F}$ = 3.1 Hz), 120.9. **^{19}F NMR** (376 MHz, CDCl3): δ -62.6. **IR (ATR):** 3059, 3027, 2955, 2924, 2854, 1731, 1600, 1457, 1314, 1259, 1167, 1128, 1058, 737, 687. **HRMS (ESI$^+$ / [M+H]$^+$):** calculated m/z ([$C_{16}H_{11}F_3N$]$^+$): 274.0839, found 274.0834.

5,6,7-trimethoxy-2-phenylquinoline (2e)

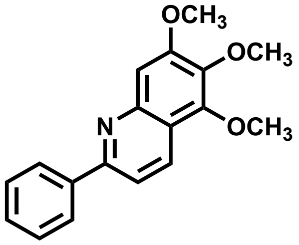

Yield: 60% (44.3 mg, 0.15 mmol). **Appearance:** White solid. **^{1}H NMR** (400 MHz, $CDCl_3$): δ 8.40 (dd, *J* = 8.6, 0.7 Hz, 1H), 8.15 – 8.07 (m, 2H), 7.72 (d, *J* = 8.7 Hz, 1H), 7.54 – 7.47 (m, 2H), 7.47 – 7.40 (m, 1H), 7.33 (m, 1H), 4.07 (s, 3H), 4.02 (s, 3H), 3.99 (s, 3H). **^{13}C NMR** (101 MHz, $CDCl_3$): δ 157.0, 156.2, 147.0, 146.1, 140.8, 139.9, 131.2, 129.2, 128.9, 127.5, 118.2, 116.8, 104.6, 61.7, 61.3, 56.2. **IR (ATR):** 3060, 2999, 2966, 2938, 2849, 2830, 1685, 1616, 1592, 1477, 1397, 1374, 1320, 1240, 1214, 1130, 1101, 1034, 995, 832, 762, 693, 650. **HRMS (ESI$^+$ / [M+H]$^+$):** calculated m/z ($[C_{18}H_{18}NO_3]^+$): 296.1282, found 296.1276.

6-(methylthio)-2-phenylquinoline (2f)

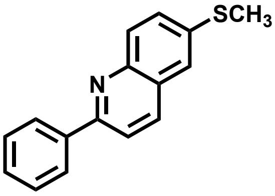

Yield: 55% (34.8 mg, 0.14 mmol). **Appearance:** Whitish-yellow solid. **^{1}H NMR** (400 MHz, $CDCl_3$): δ 8.18 – 8.12 (m, 2H), 8.06 (d, *J* = 8.8 Hz, 2H), 7.83 (d, J = 8.6 Hz, 1H), 7.60 (dd, J = 8.9, 2.2 Hz, 1H), 7.56 – 7.49 (m, 3H), 7.49 – 7.43 (m, 1H), 2.59 (s, 3H). **^{13}C NMR** (101 MHz, $CDCl_3$): δ 156.5, 146.6, 139.6, 137.1, 135.5, 130.0, 129.3, 129.2, 128.9, 127.7, 127.5, 122.3, 119.6, 15.8. **IR (ATR):** 3050, 3024, 2958, 2922, 2853, 1735, 1610, 1589, 1578, 1552, 1480, 1446, 1392, 1319, 1192, 1132, 1073, 1021, 951, 869, 829, 787, 757, 693, 572. **HRMS (ESI$^+$ / [M+H]$^+$):** calculated m/z ($[C_{16}H_{14}NS]^+$): 252.0842, found 252.0836.

2,6-diphenylquinoline (2g)

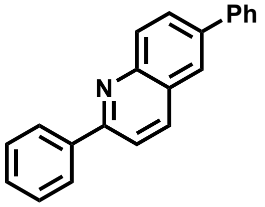

Yield: 59% (41.6 mg, 0.15 mmol). **Appearance:** Yellow solid. **^{1}H NMR** (400 MHz, $CDCl_3$): δ 8.26 (t, *J* = 8.8 Hz, 2H), 8.19 (d, *J* = 7.1 Hz, 2H), 8.06 – 7.98 (m, 2H), 7.91 (d, *J* = 8.5 Hz, 1H), 7.75 (d, *J* = 7.2 Hz, 2H), 7.61 – 7.45 (m, 5H), 7.42 (t, *J* = 7.3 Hz, 1H).

^{13}C NMR (101 MHz, $CDCl_3$): δ 157.5, 147.9, 140.6, 139.8, 139.1, 137.1, 130.3, 129.5, 129.5, 129.1, 129.0, 127.8, 127.7, 127.6, 127.5, 125.3, 119.5. **IR (ATR):** 3052, 3034, 2959, 2921, 2851, 1737, 1594, 1575, 1556, 1485, 1441, 1258, 1075, 1024, 1017, 889, 836, 826, 792, 761, 692. **HRMS (ESI^+ / $[M+H]^+$):** calculated m/z ($[C_{21}H_{16}N]^+$): 282.1278, found 282.1271

6-phenyl-[1,3]dioxolo[4,5-*g*]quinoline (2h)

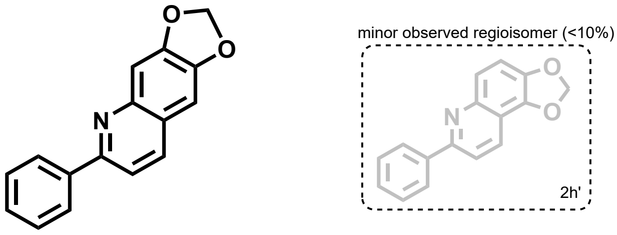

Yield: 59% (36.9 mg, 0.15 mmol). **Appearance:** White solid. **^{1}H NMR** (400 MHz, $CDCl_3$): δ 8.11 (d, *J* = 6.8 Hz, 2H), 8.01 (d, *J* = 8.4 Hz, 1H), 7.71 (d, *J* = 8.5 Hz, 1H), 7.56 – 7.48 (m, 2H), 7.48 – 7.39 (m, 2H), 7.06 (s, 1H), 6.10 (s, 2H). **^{13}C NMR** (101 MHz, $CDCl_3$): δ 155.5, 150.9, 147.8, 146.7, 139.9, 135.6, 129.0, 128.9, 127.4, 124.2, 117.4, 106.3, 102.7, 101.8. **IR (ATR):** 3056, 3042, 3002, 2960, 2901, 2853, 2778, 1616, 1494, 1487, 1466, 1455, 1418, 1398, 1387, 1266, 1230, 1170, 1041, 1024, 953, 920, 862, 855, 756, 693. **HRMS (ESI^+ / $[M+H]^+$):** calculated m/z ($[C_{16}H_{12}NO_2]^+$): 250.0863, found 250.0857.

3-phenylbenzo[*f*]quinoline (2i)

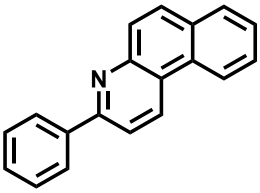

Yield: 35% (22.3 mg, 0.09 mmol). **Appearance:** White solid. **^{1}H NMR** (400 MHz, $CDCl_3$): 9.52 (d, *J* = 8.1 Hz, 1H), 8.41 – 8.32 (m, 2H), 8.22 (d, *J* = 8.3 Hz, 1H), 8.01 (d, *J* = 8.3 Hz, 1H), 7.95 – 7.89 (m, 1H), 7.83 – 7.67 (m, 4H), 7.62 – 7.55 (m, 2H), 7.53 – 7.46 (m, 1H). **^{13}C NMR** (101 MHz, $CDCl_3$): 155.6, 146.4, 139.9, 136.6, 134.0, 132.0, 129.3, 129.0, 128.3, 127.9, 127.6, 127.0, 125.3, 125.2, 124.9, 119.0. **IR (ATR):** 3056, 3032, 2961, 2925, 2852, 1730, 1592, 1558, 1489, 1446, 1412, 1389, 1268, 1262, 1235, 1156, 1103, 1078, 1025, 1015, 843, 801, 747, 726, 688. **HRMS (ESI^+ / $[M+H]^+$):** calculated m/z ($[C_{19}H_{14}N]^+$): 256.1121, found 256.1115.

2-methoxy-6-phenyl-1,5-naphthyridine (2j)

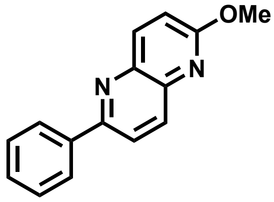

Yield: 36% (18.6 mg, 0.09 mmol). **Appearance:** Whitish-yellow solid. **^{1}H NMR** (400 MHz, $CDCl_3$): δ 8.26 (d, *J* = 9.0, 1H), 8.19 (d, *J* = 8.8, 1H), 8.16 – 8.08 (m, 2H), 8.00 (d, *J* = 8.7 Hz, 1H), 7.57 – 7.48 (m, 2H), 7.48 – 7.42 (m, 1H), 7.14 (d, *J* = 9.0 Hz, 1H), 4.10 (s, 3H). **^{13}C NMR** (101 MHz, $CDCl_3$): δ 162.5, 155.2, 141.9, 141.3, 140.3, 139.4, 135.7, 129.3, 129.0, 127.4, 122.1, 116.9, 53.9. **IR (ATR):** 3056, 3034, 3003, 2984, 2944, 2925, 2854, 1739, 1613, 1594, 1580, 1510, 1481, 1462, 1447, 1391, 1320, 1256, 1117, 1058, 1028, 847, 830, 800, 765, 709, 697, 633. **HRMS (ESI^+ / $[M+H]^+$):** calculated m/z ($[C_{15}H_{13}N_2O]^+$): 237.1023, found 237.1017.

2-(furan-2-yl)-6-methoxyquinoline (2k)

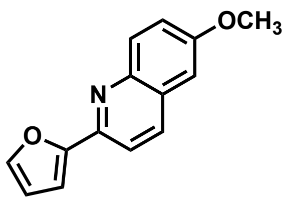

Yield: 12% (6.8 mg, 0.03 mmol). **Appearance:** Brown oil. **^{1}H NMR** (400 MHz, $CDCl_3$): δ 8.11-7.99 (m, 2H), 7.77 (d, *J* = 8.5 Hz, 1H), 7.60 (s, 1H), 7.36 (dd, *J* = 9.3, 2.8 Hz, 1H), 7.14 (d, *J* = 3.4 Hz, 1H), 7.05 (d, *J* = 2.8 Hz, 1H), 6.57 (dd, *J* = 3.5, 1.8 Hz, 1H), 3.93 (s, 3H). **IR (ATR):** 3114, 3060, 3002, 2957, 2925, 2850, 1725, 1624, 1601, 1502, 1493, 1471, 1378, 1260, 1250, 1228, 1163, 1085, 1028, 1010, 855, 832, 741. **HRMS (ESI^+ / $[M+H]^+$):** calculated m/z ($[C_{14}H_{12}NO_2]^+$): 226.0863, found 226.0858.

NMR Spectra

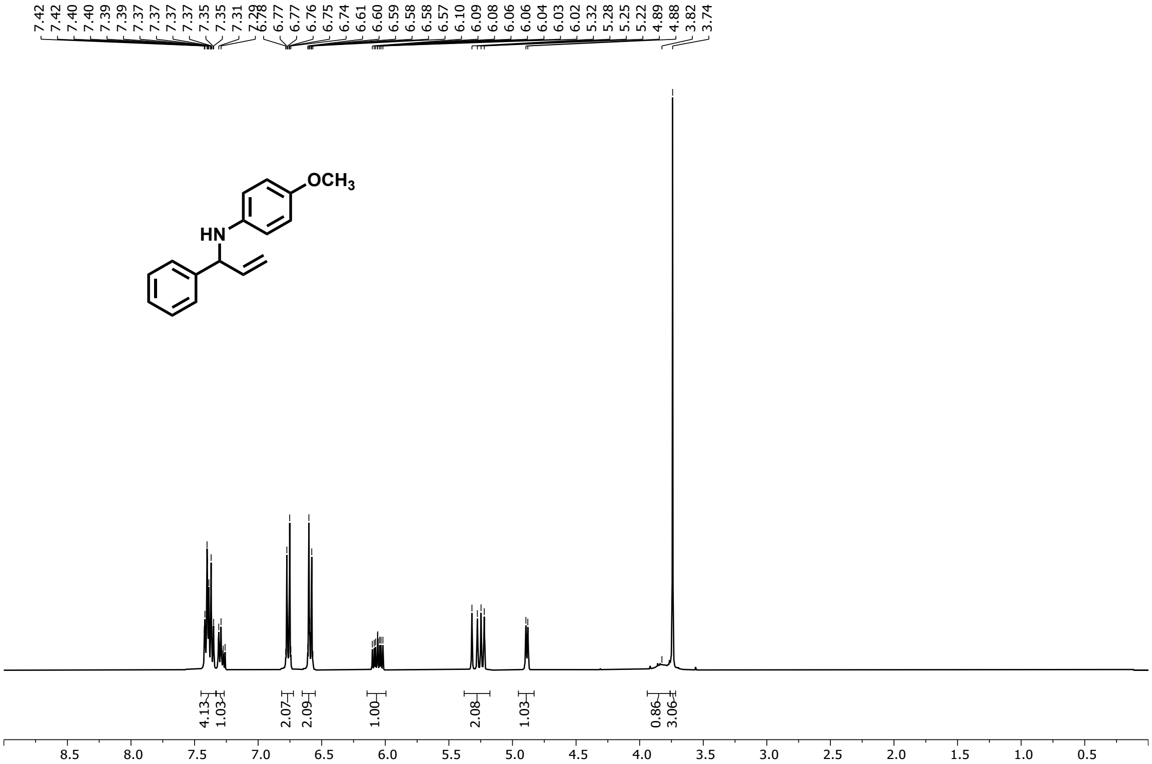

Spectrum 1 - ^{1}H NMR (400 MHz, $CDCl_3$) of compound ***1a***

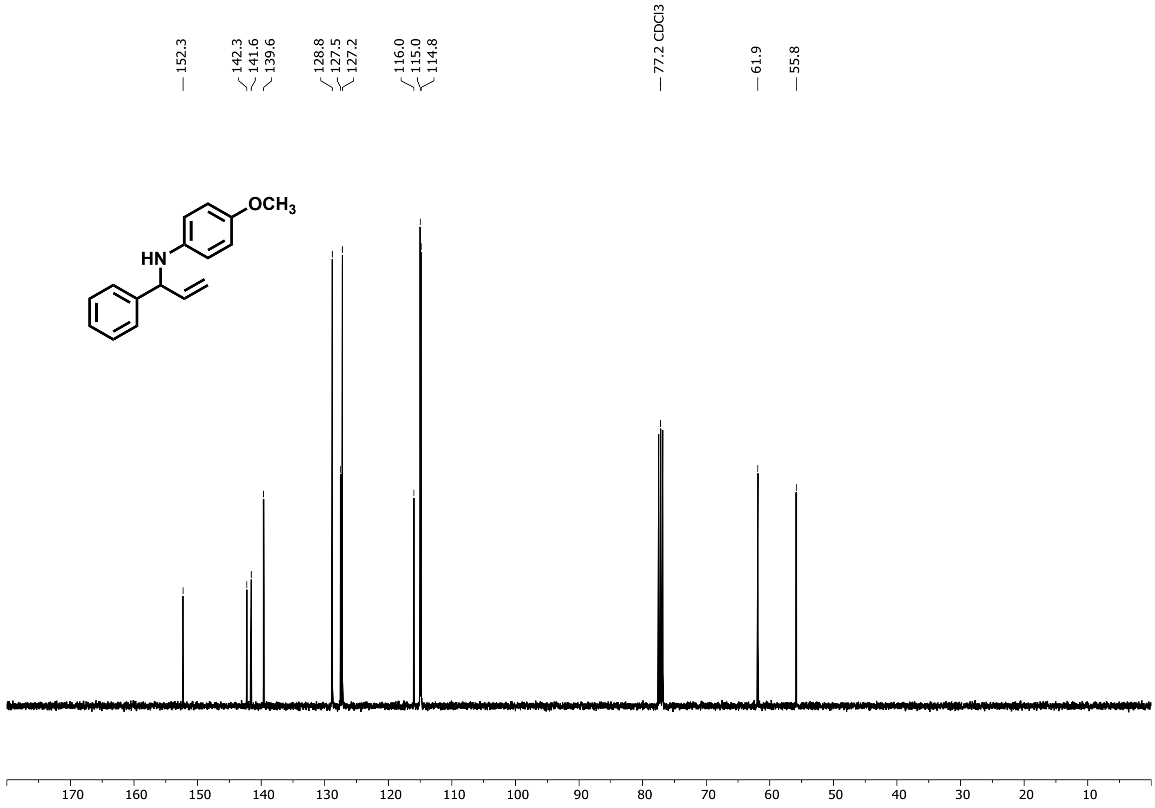

Spectrum 2 - ^{13}C NMR (101 MHz, $CDCl_3$) of compound ***1a***

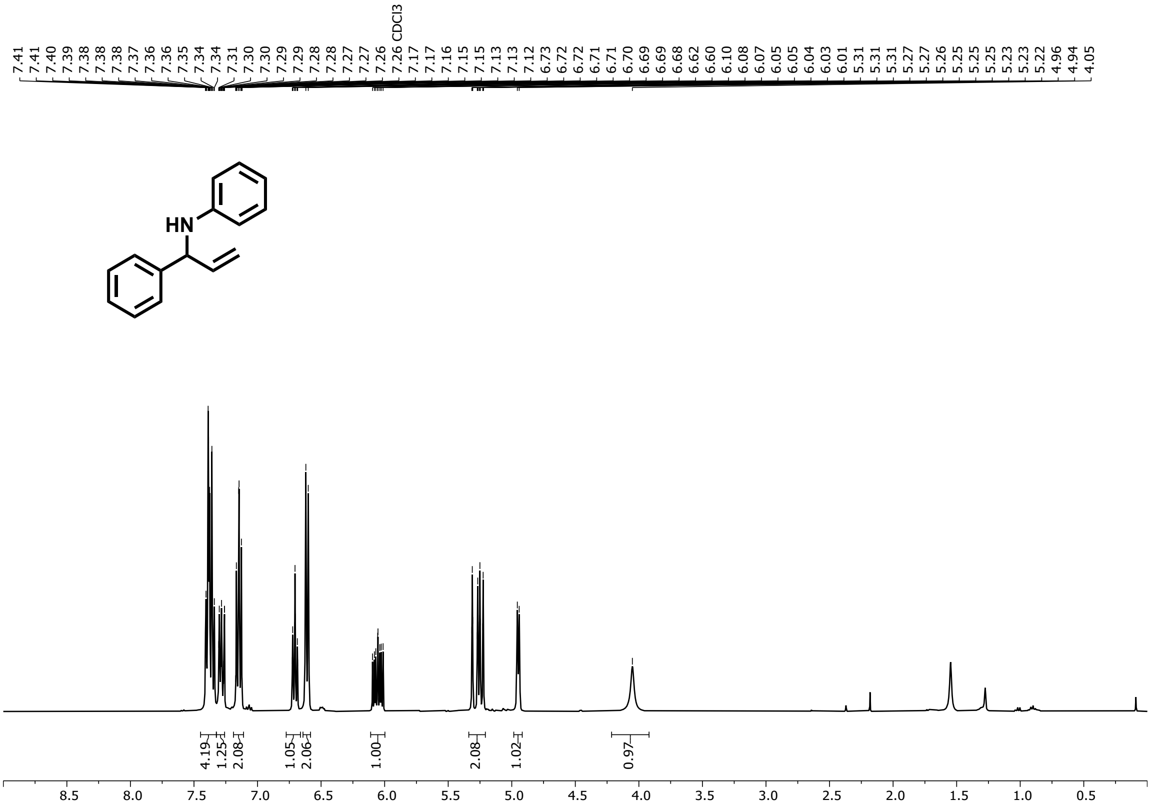

Spectrum 3 - ^{1}H NMR (400 MHz, $CDCl_3$) of compound ***1b***

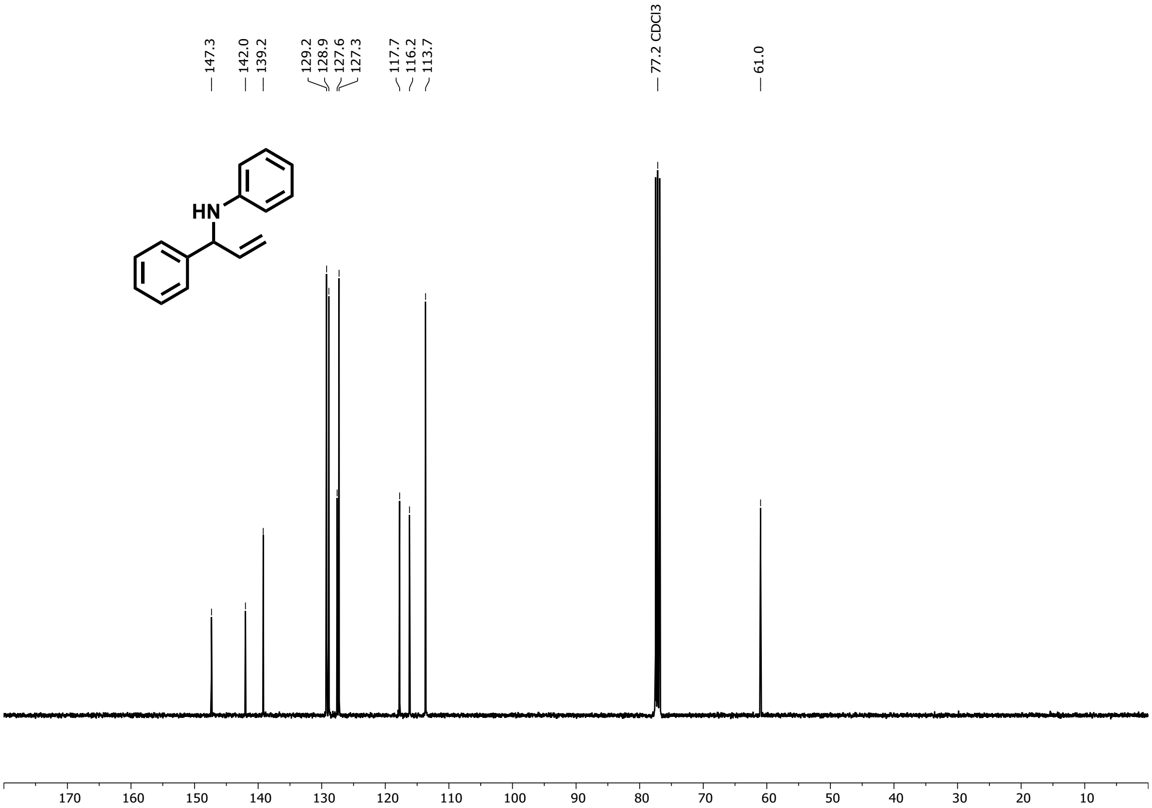

*Spectrum 4 - ^{13}C NMR (101 MHz, $CDCl_3$) of compound **1b***

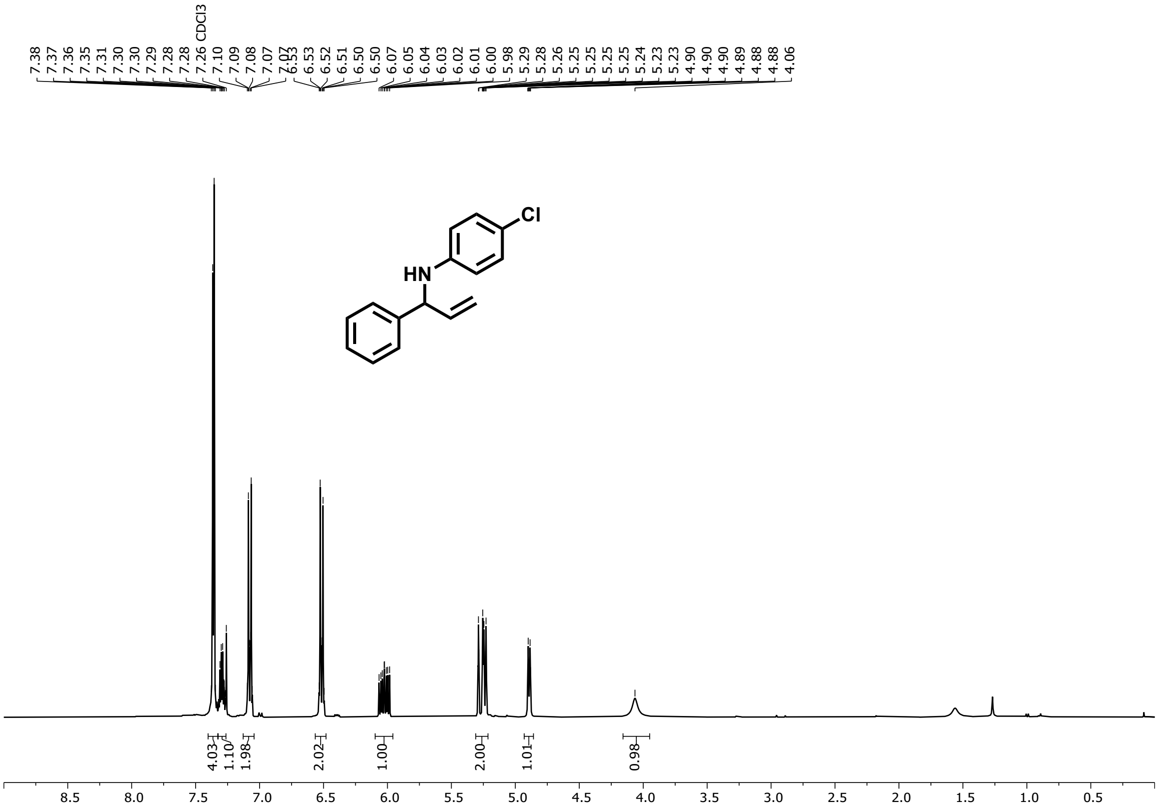

*Spectrum 5 - ^{1}H NMR (400 MHz, $CDCl_3$) of compound **1c***

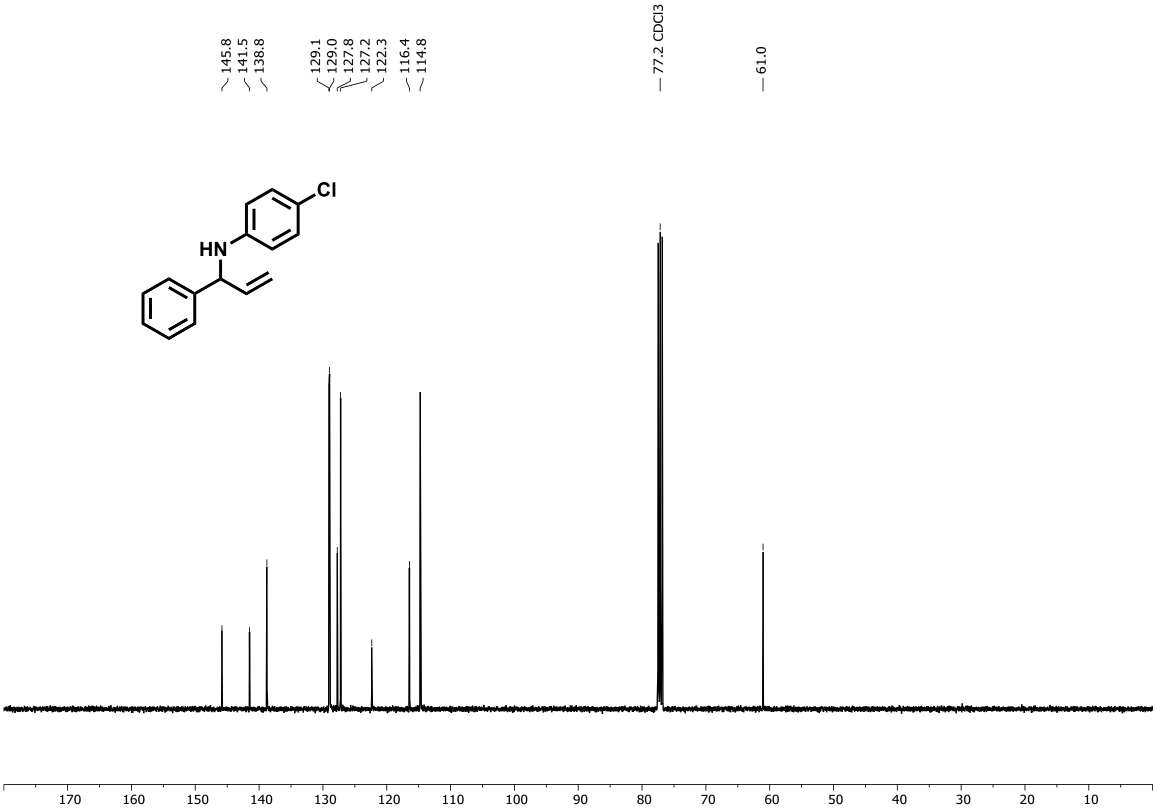

*Spectrum 6 - ^{13}C NMR (101 MHz, $CDCl_3$) of compound **1c***

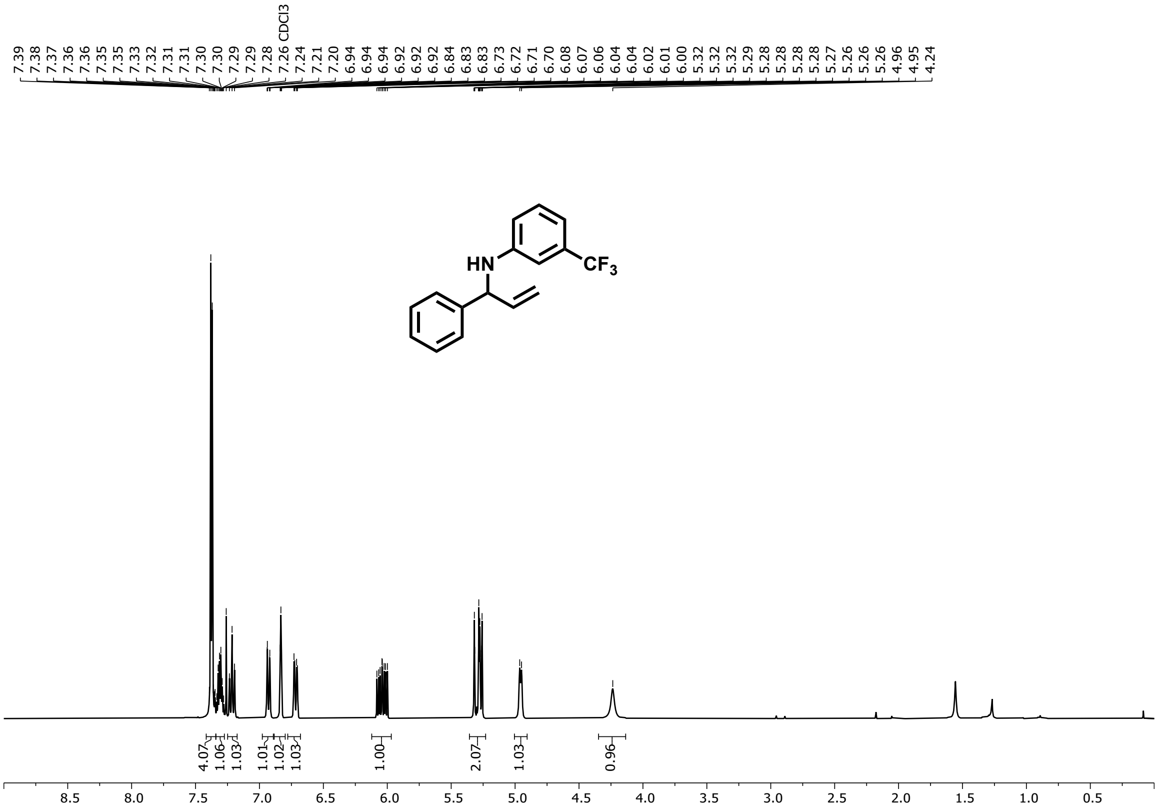

*Spectrum 7 - ^{1}H NMR (400 MHz, $CDCl_3$) of compound **1d***

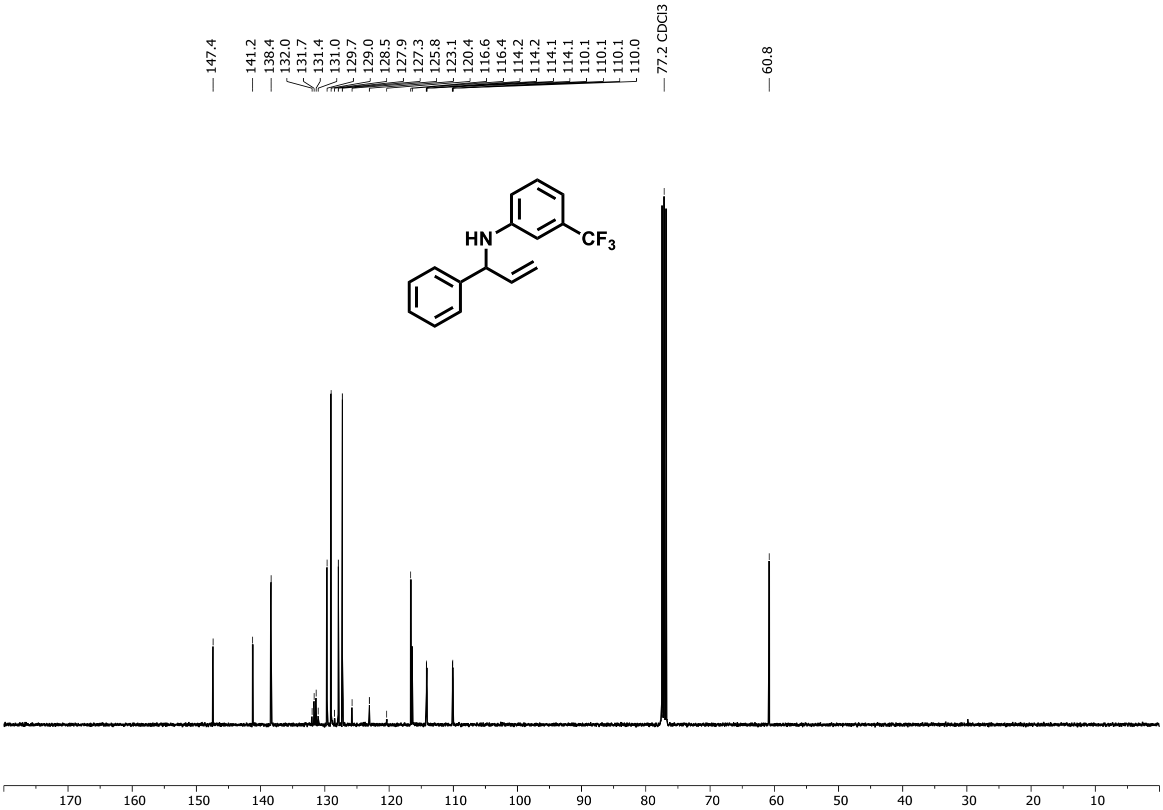

*Spectrum 8 - ^{13}C NMR (101 MHz, $CDCl_3$) of compound **1d***

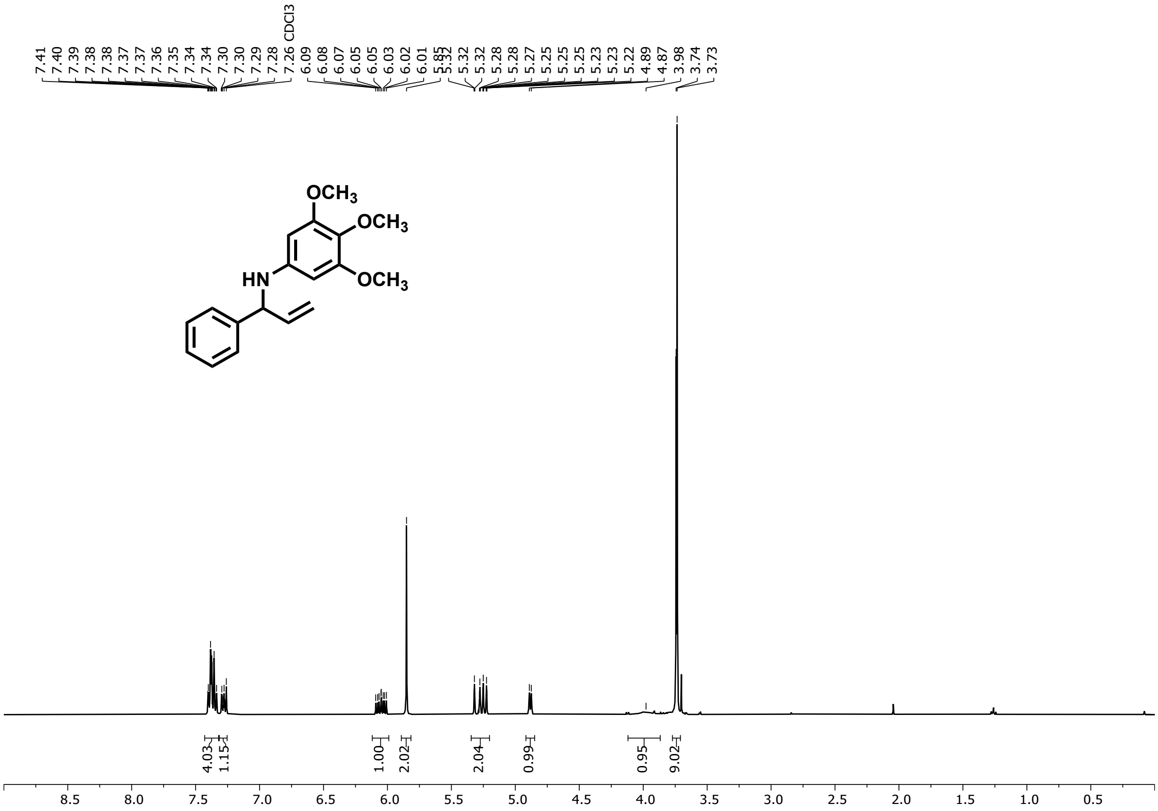

*Spectrum 9 - ^{1}H NMR (400 MHz, CDCl$_3$) of compound **1e***

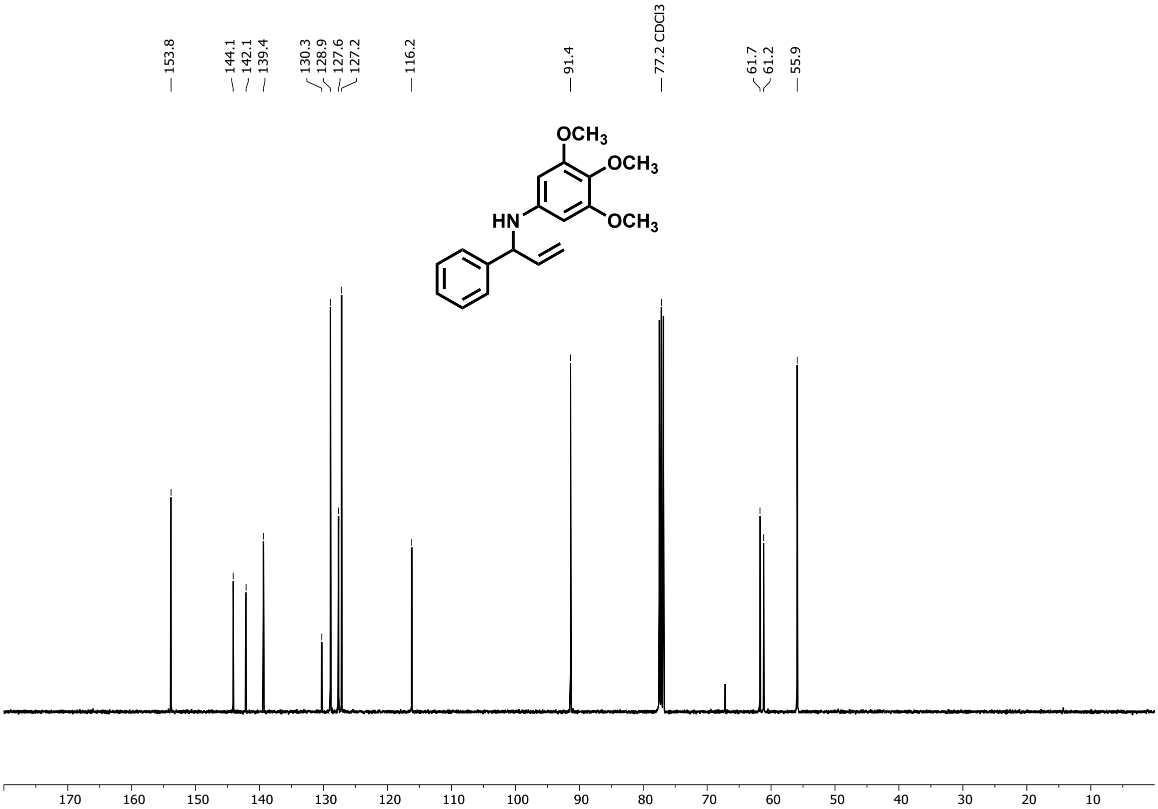

*Spectrum 10 - ^{13}C NMR (101 MHz, CDCl$_3$) of compound **1e***

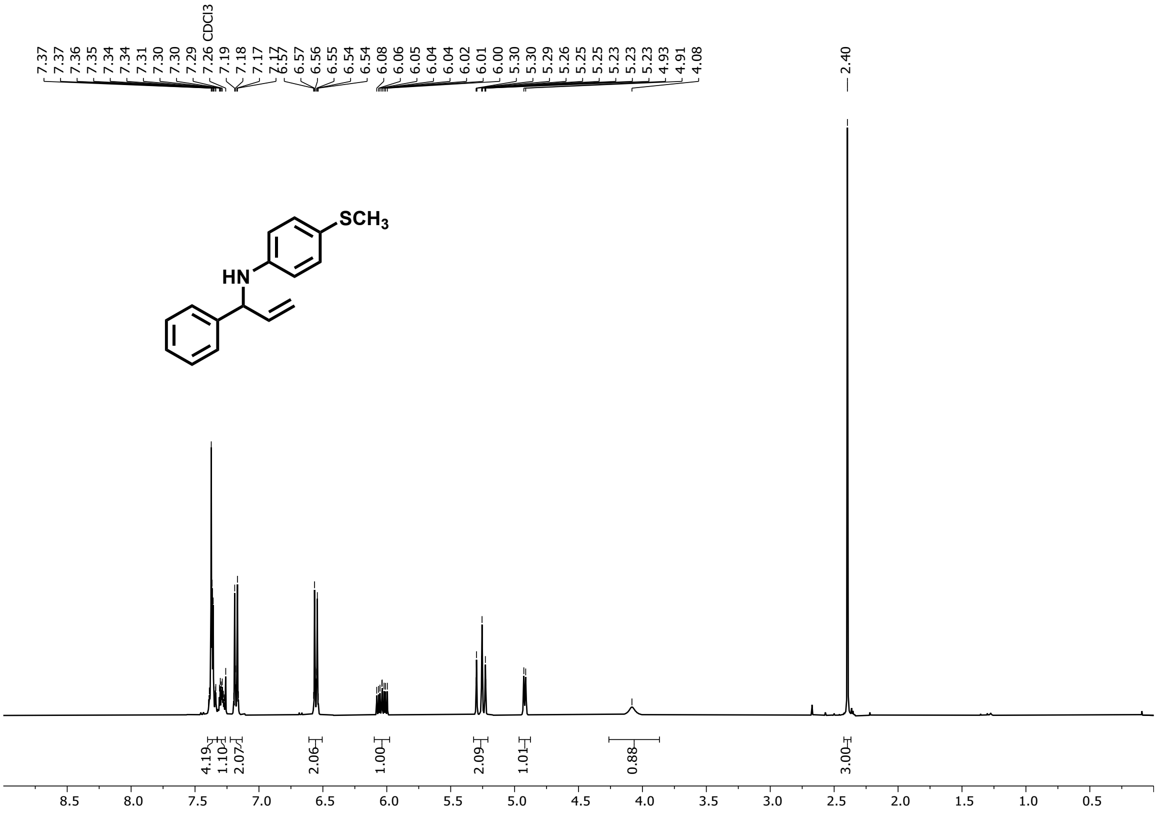

*Spectrum 11 - ^{1}H NMR (400 MHz, $CDCl_3$) of compound **1f***

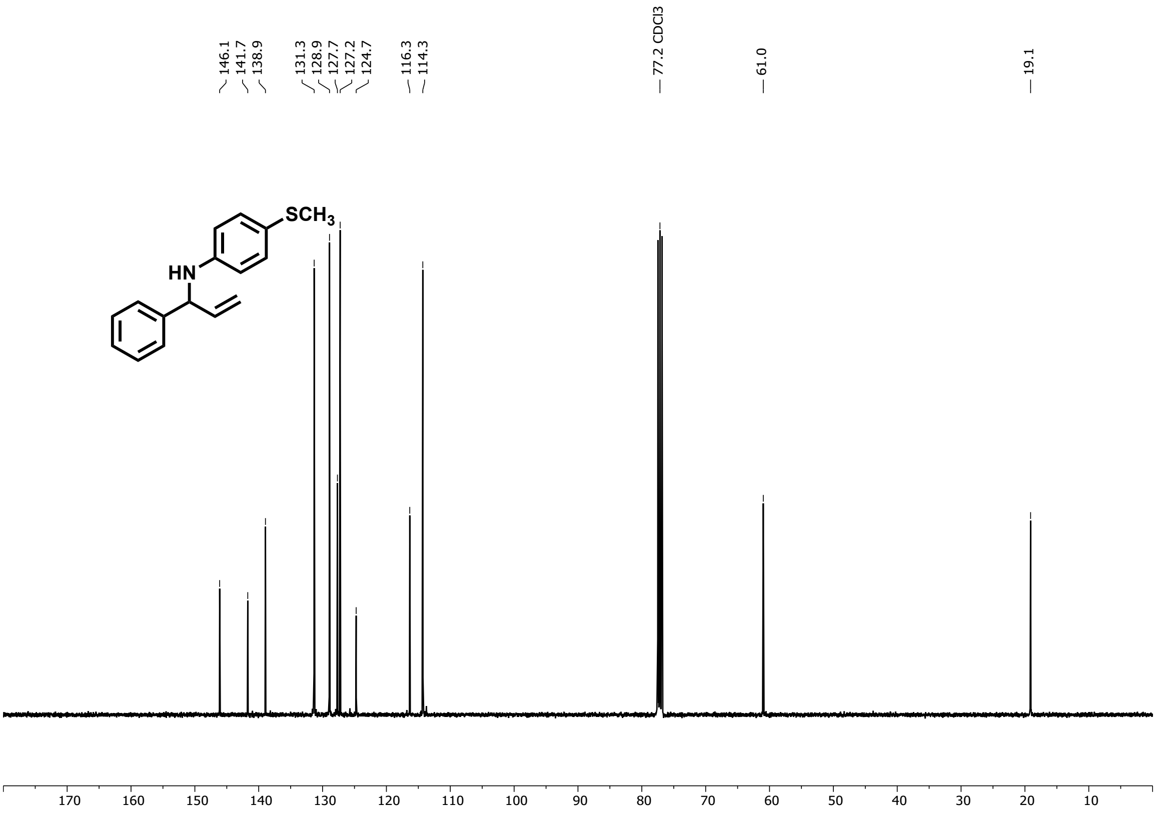

*Spectrum 12 - ^{13}C NMR (101 MHz, $CDCl_3$) of compound **1f***

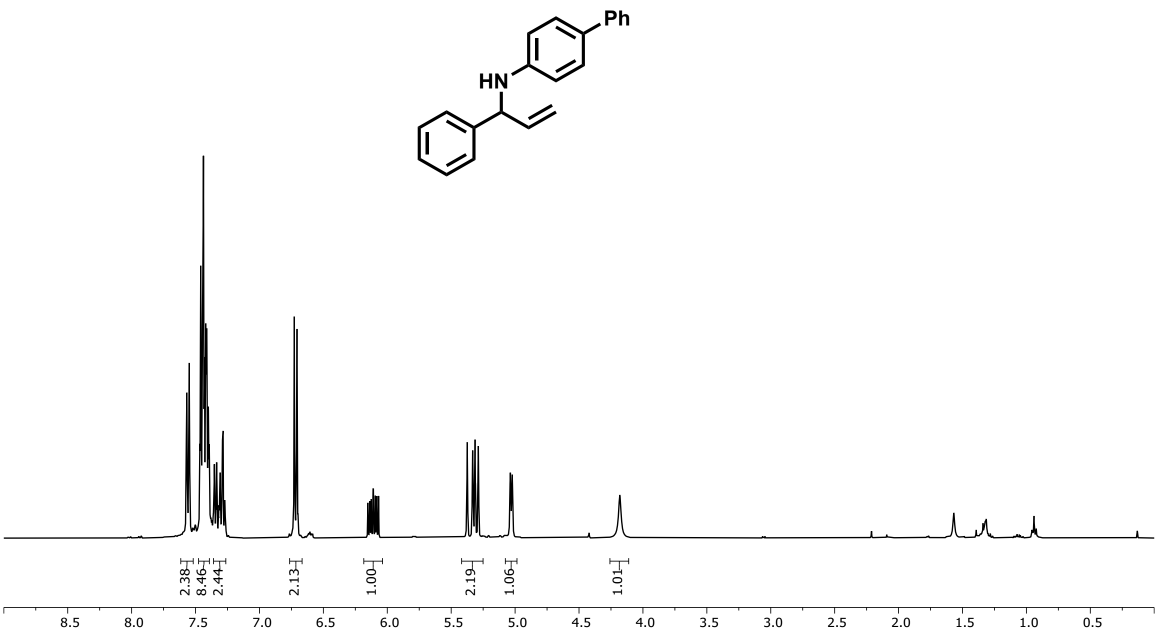

Spectrum 13 - ^{1}H NMR (400 MHz, $CDCl_3$) of compound ***1g***

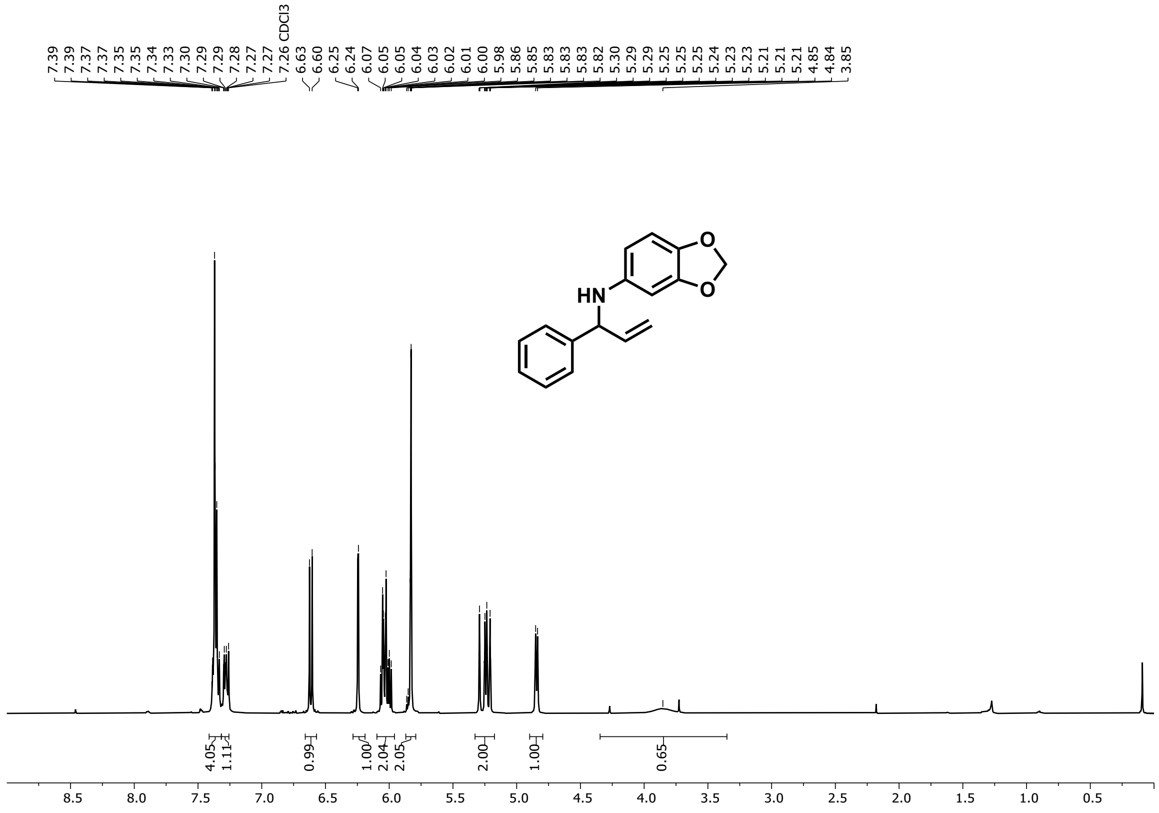

*Spectrum 14 - ^{1}H NMR (400 MHz, $CDCl_3$) of compound **1h***

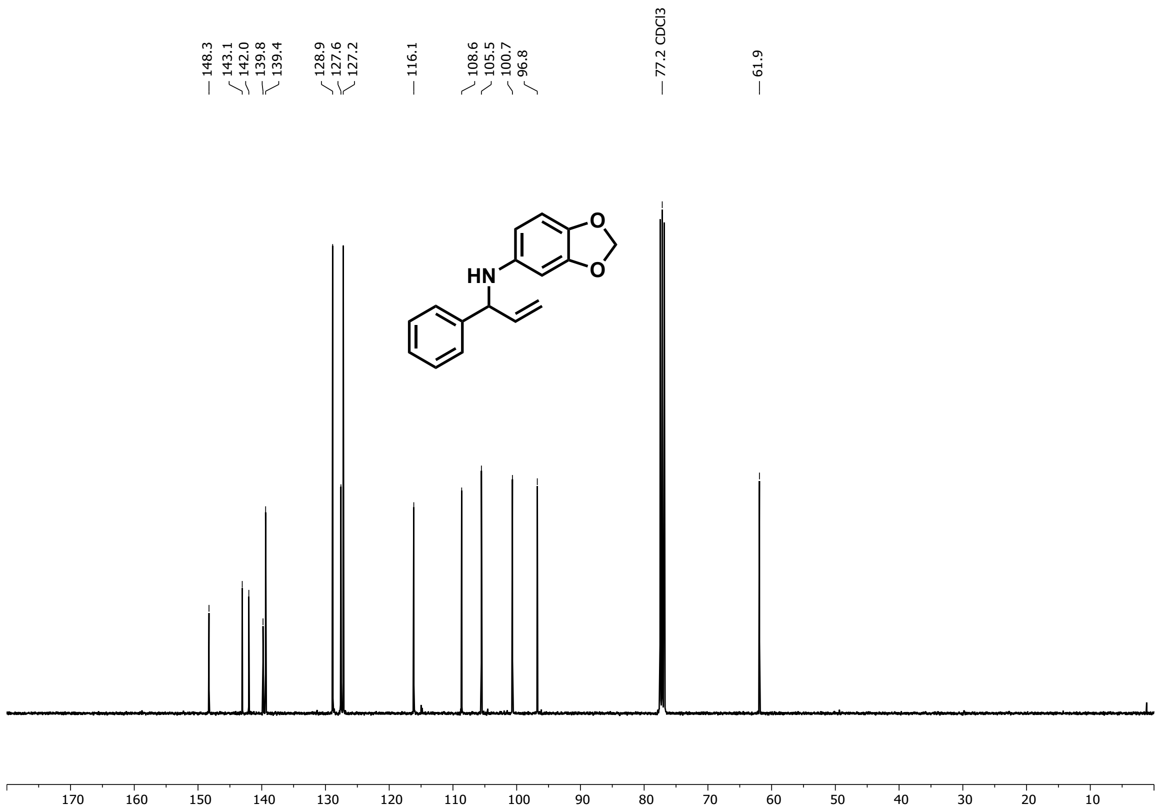

*Spectrum 15 - ^{13}C NMR (101 MHz, $CDCl_3$) of compound **1h***

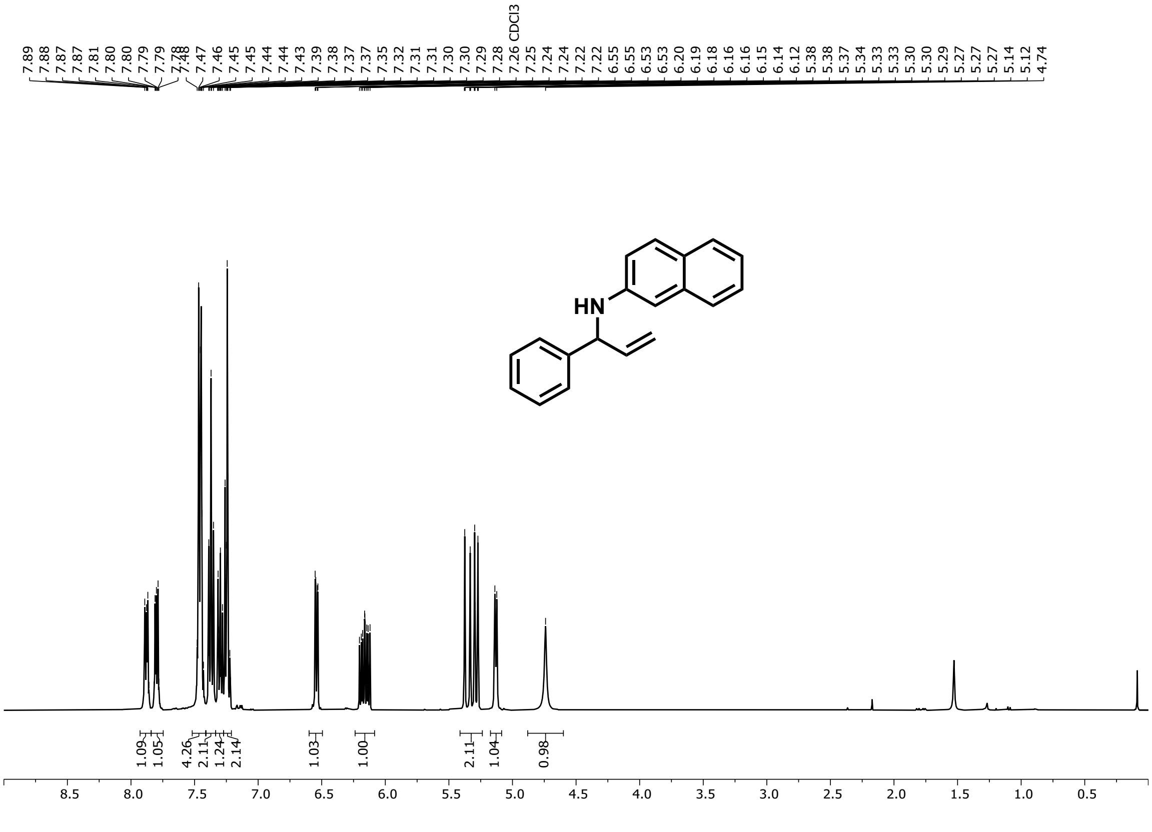

*Spectrum 16 - ¹H NMR (400 MHz, $CDCl_3$) of compound **1i***

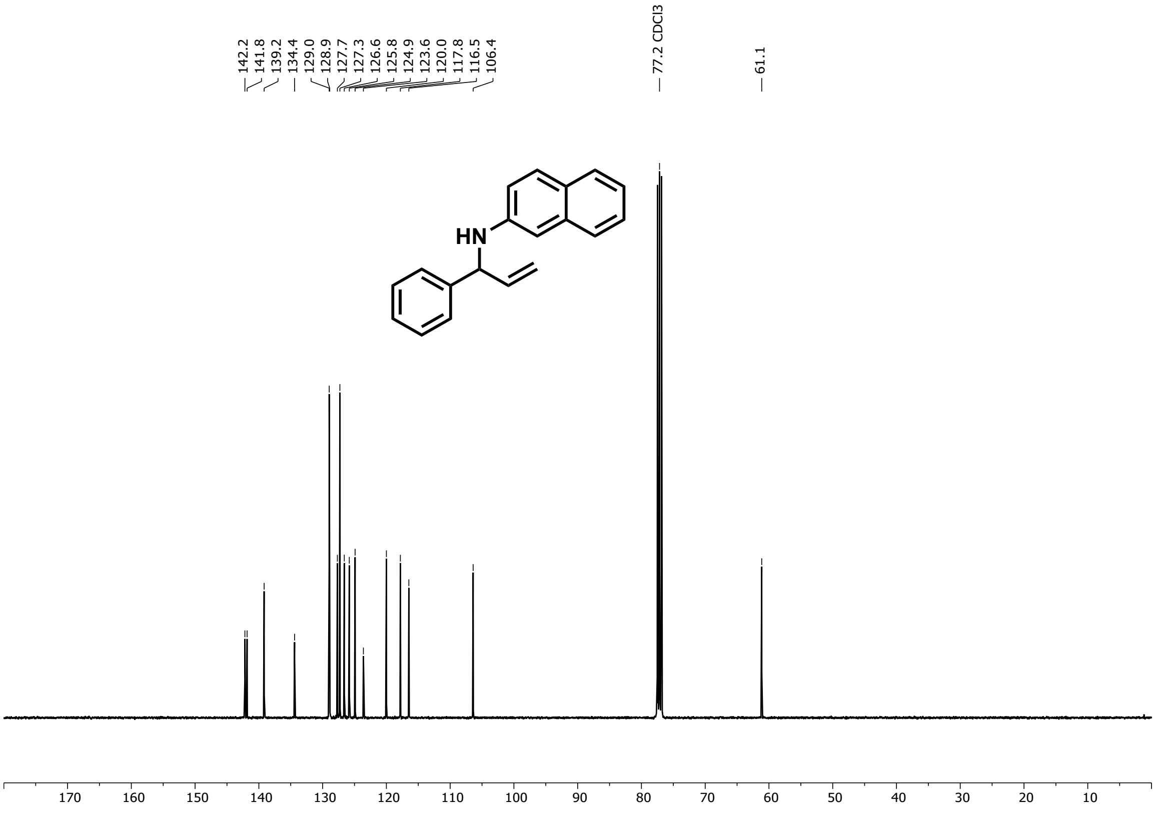

*Spectrum 17 - ¹³C NMR (101 MHz, $CDCl_3$) of compound **1i***

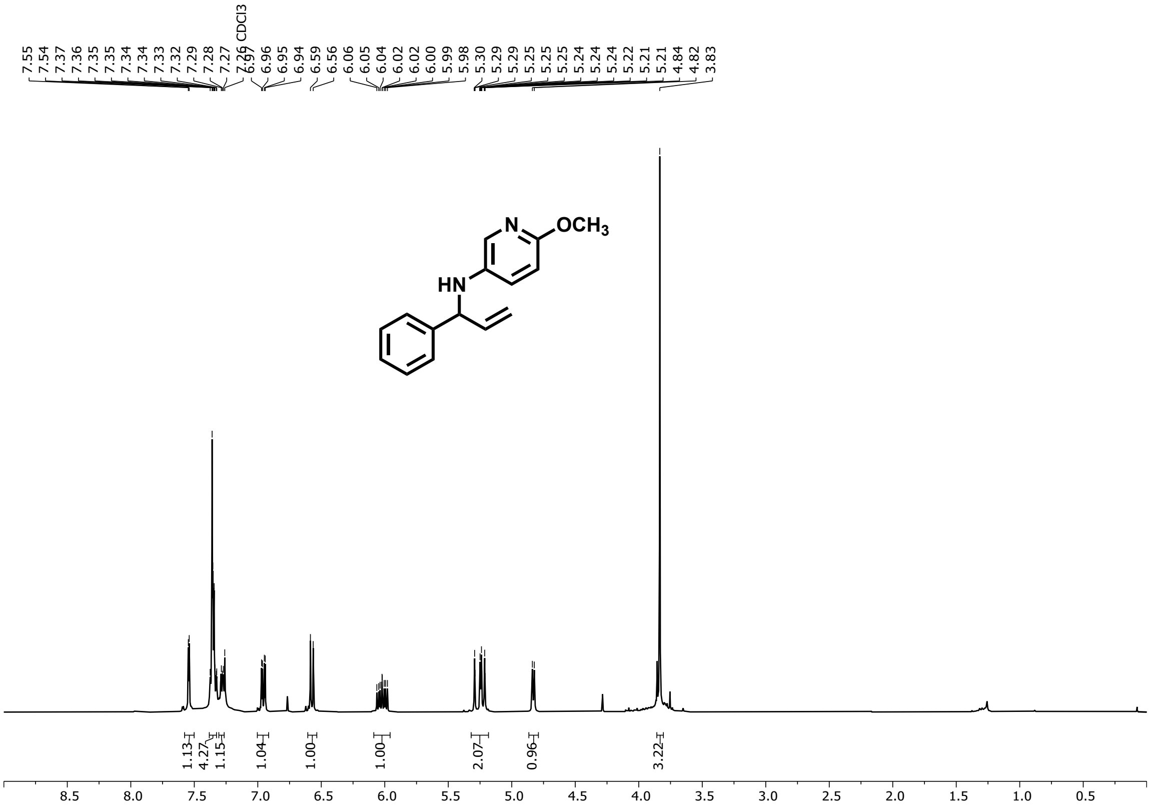

Spectrum 18 - ^{1}H NMR (400 MHz, $CDCl_3$) of compound ***1j***

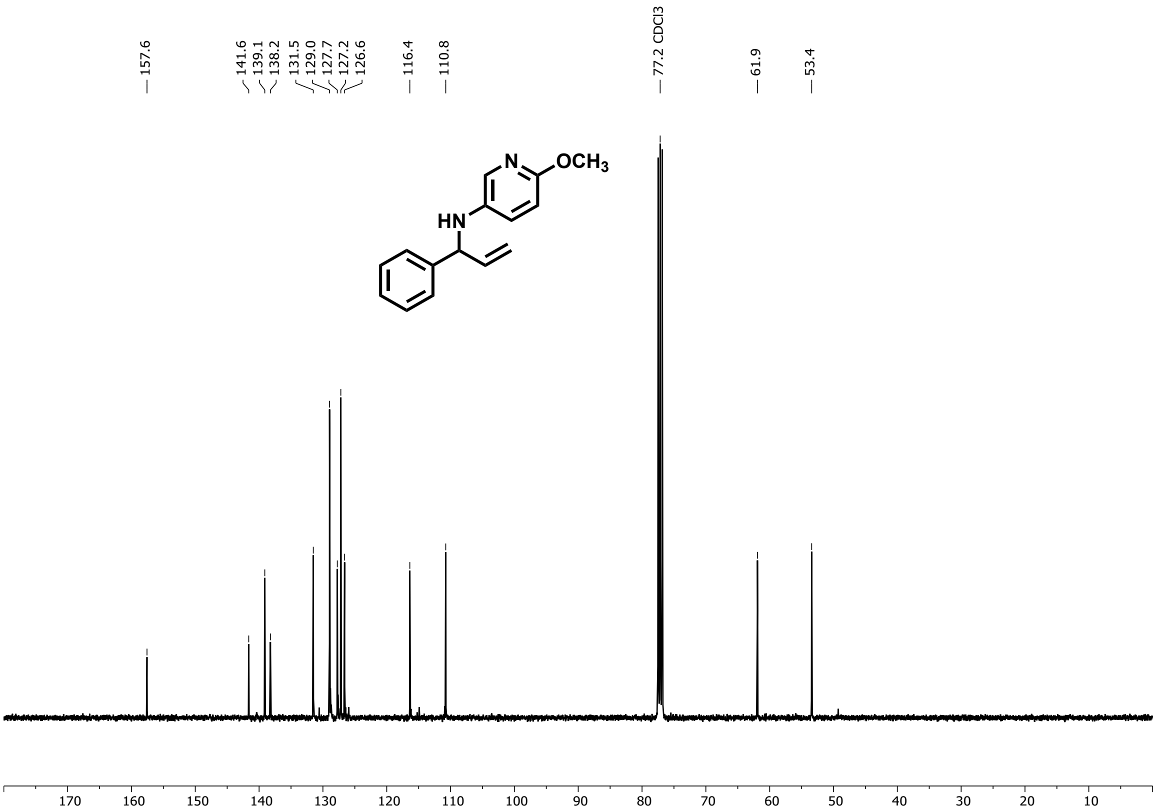

Spectrum 19 - ^{13}C NMR (101 MHz, $CDCl_3$) of compound ***1j***

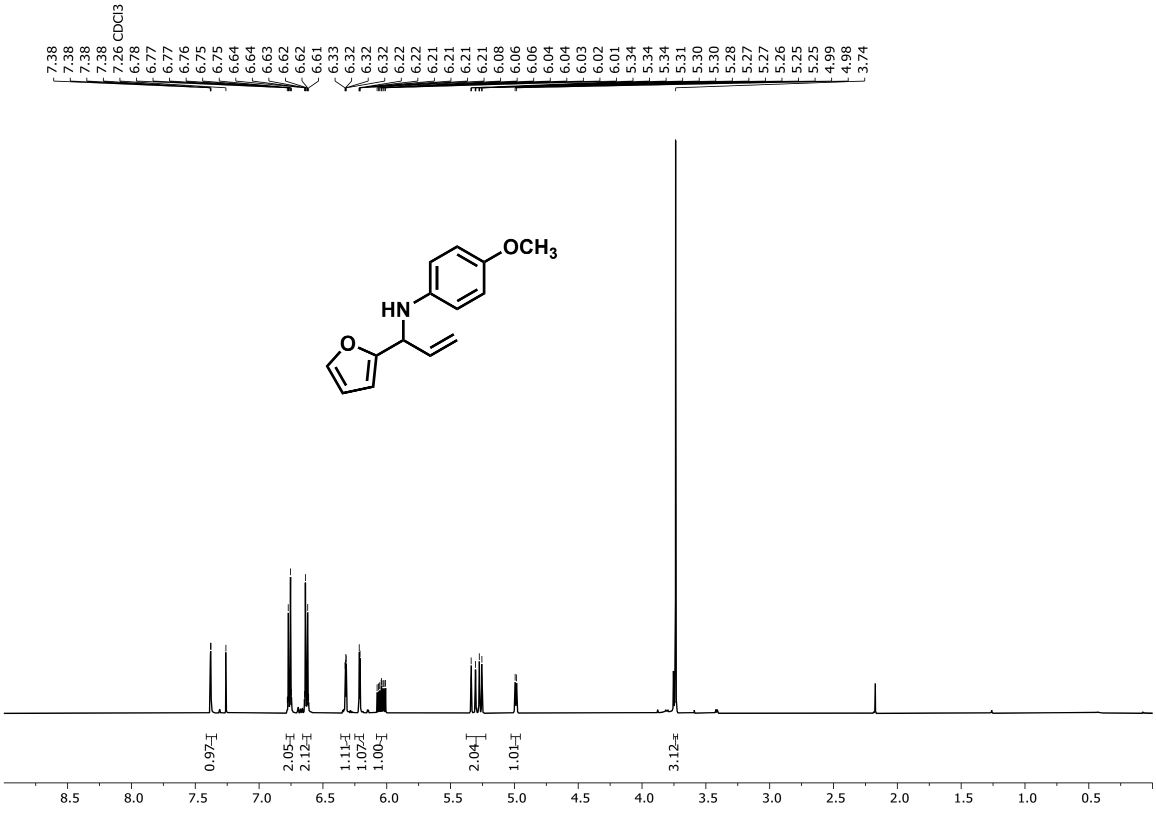

*Spectrum 20 - ^{1}H NMR (500 MHz, $CDCl_3$) of compound **1k***

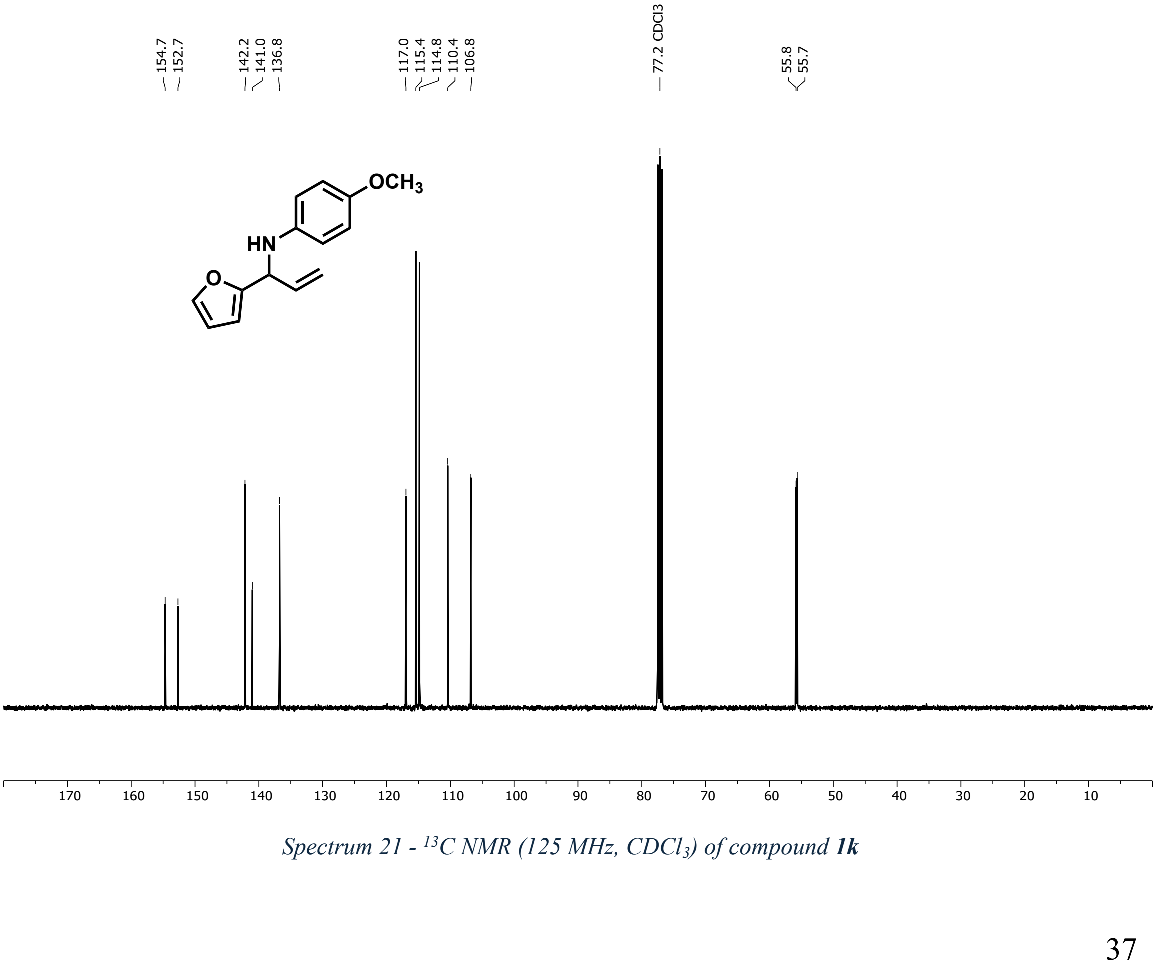

*Spectrum 21 - ^{13}C NMR (125 MHz, $CDCl_3$) of compound **1k***

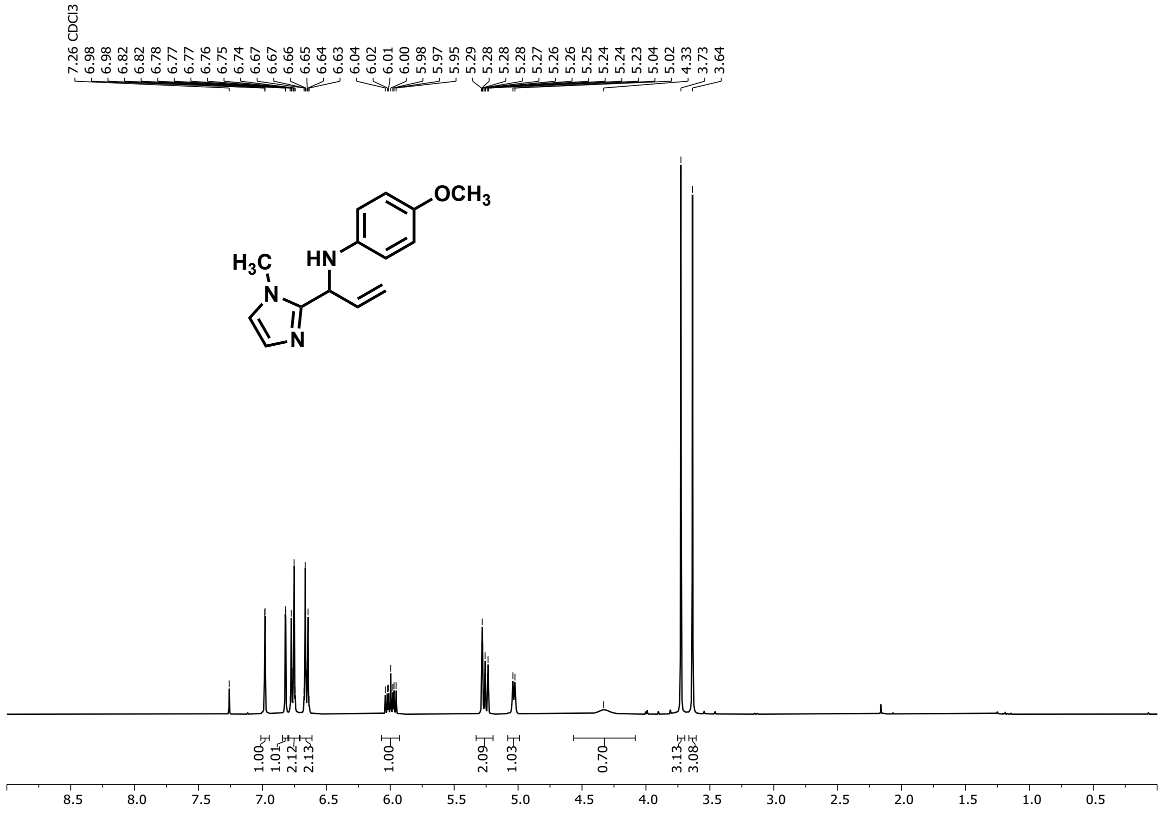

*Spectrum 22 - ^{1}H NMR (400 MHz, $CDCl_3$) of compound **1l***

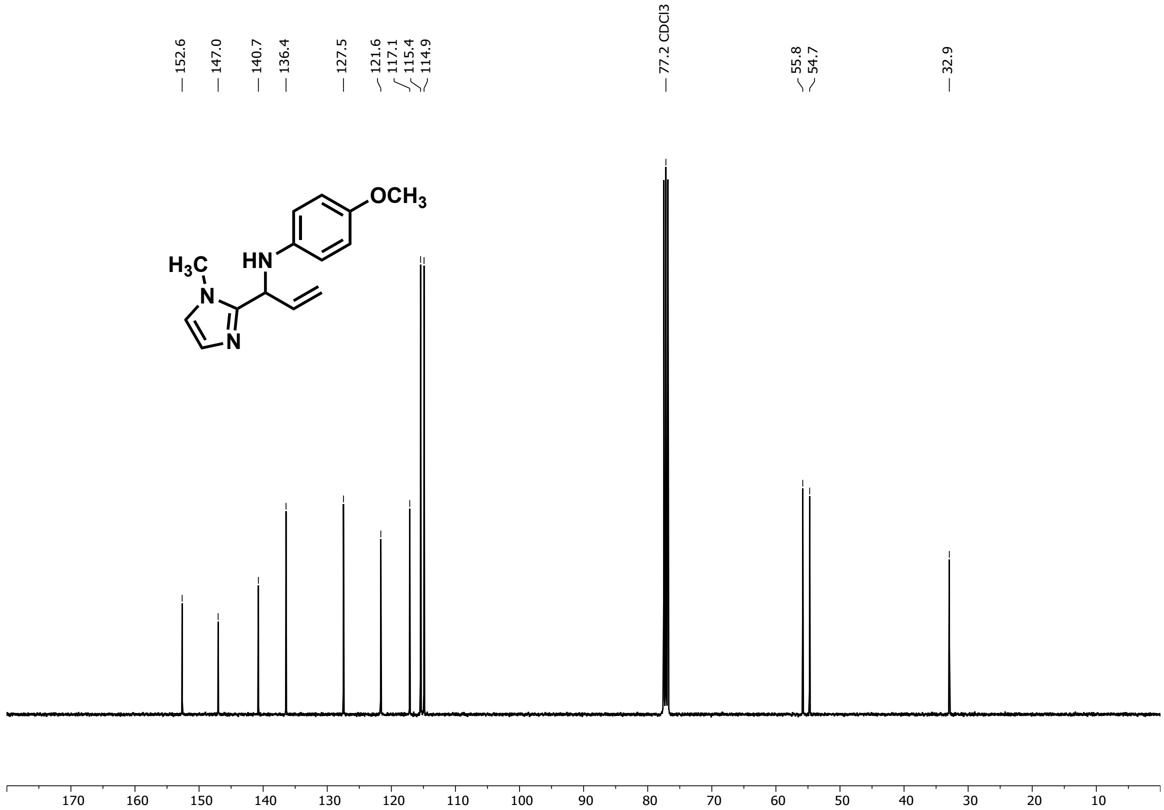

*Spectrum 23 - ^{13}C NMR (101 MHz, $CDCl_3$) of compound **1l***

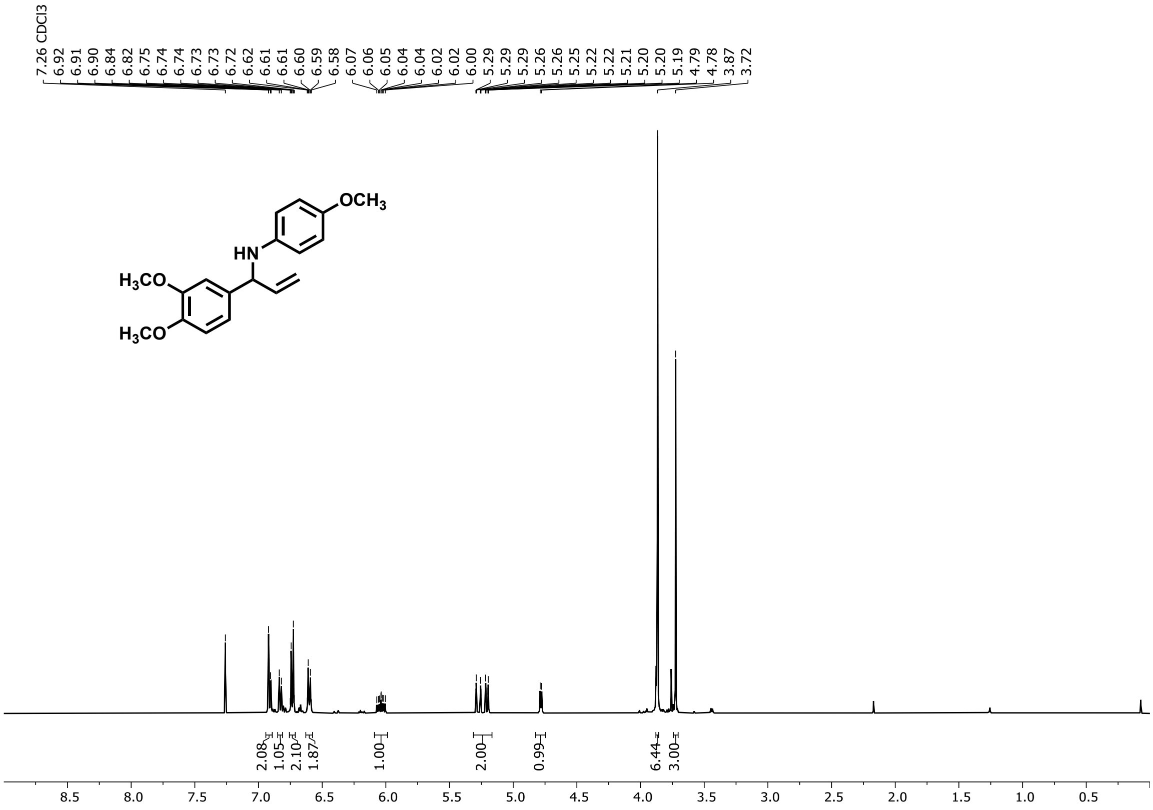

*Spectrum 24 - ^{1}H NMR (500 MHz, $CDCl_3$) of compound **1m***

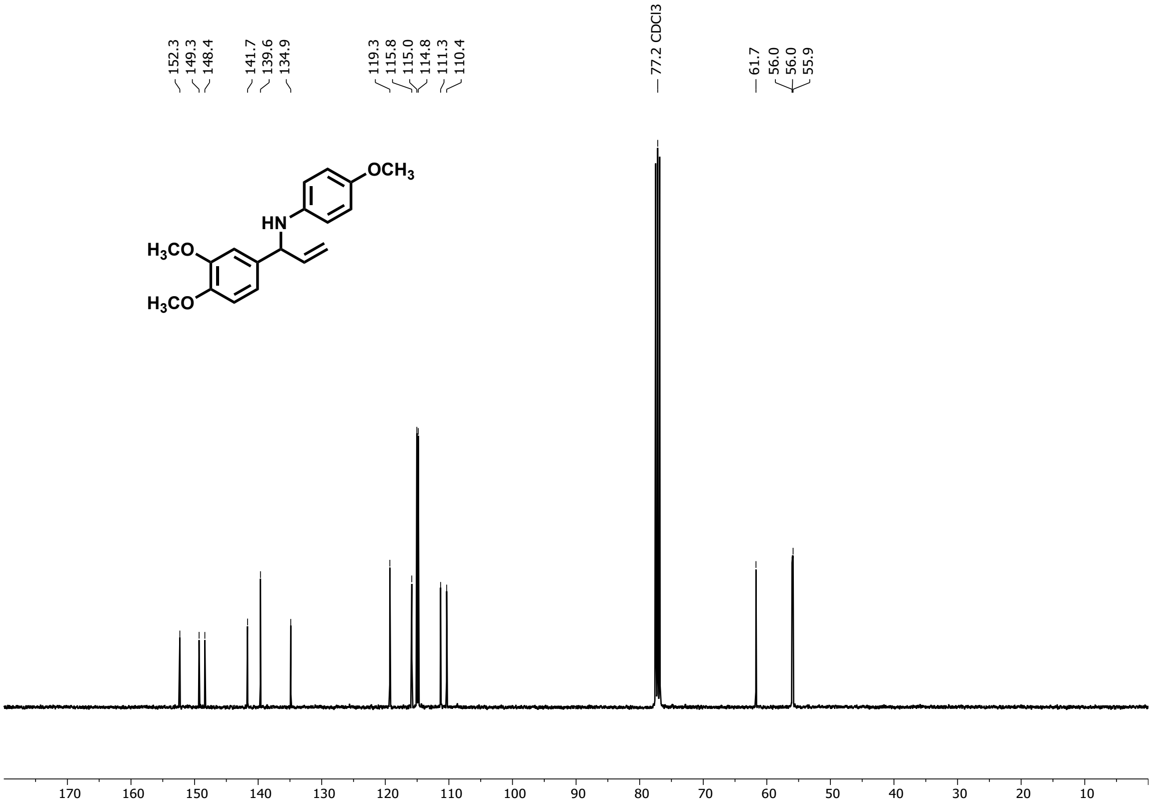

*Spectrum 25 - ^{13}C NMR (101 MHz, $CDCl_3$) of compound **1m***

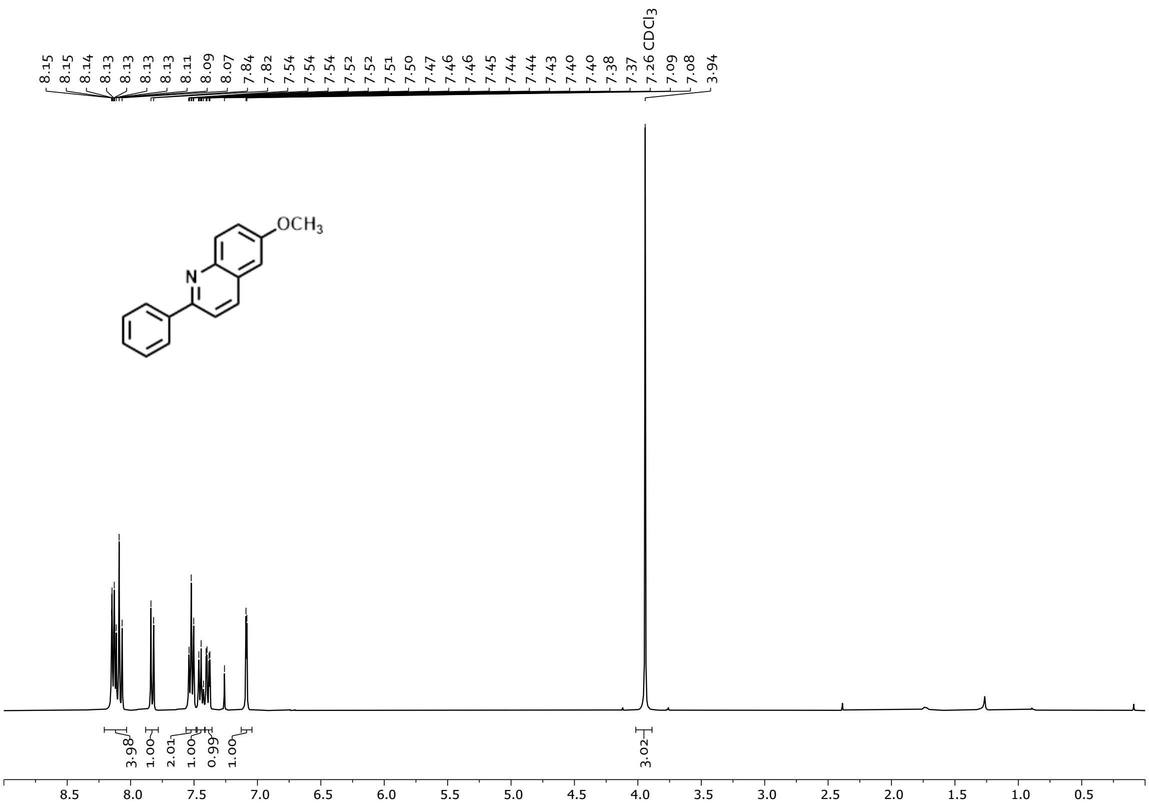

*Spectrum 26 - ^{1}H NMR (400 MHz, $CDCl_3$) of compound **2a***

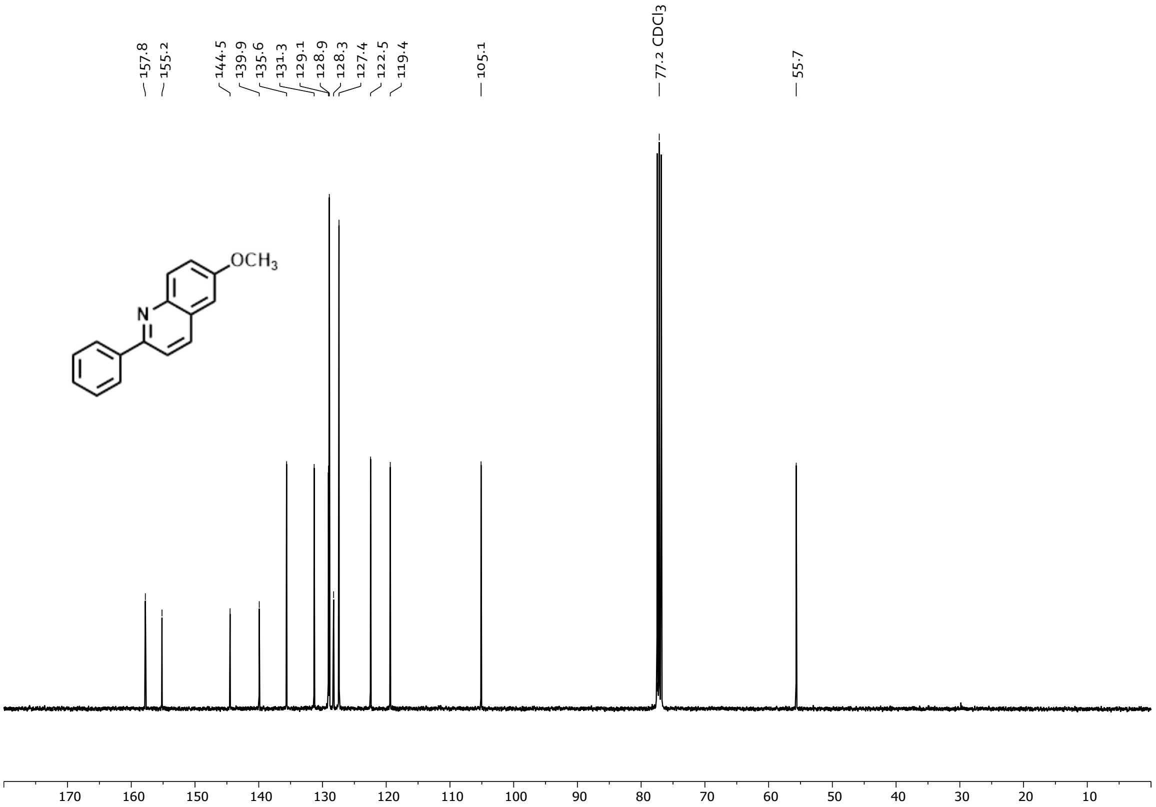

*Spectrum 27 – ^{13}C NMR (101 MHz, $CDCl_3$) of compound **2a***

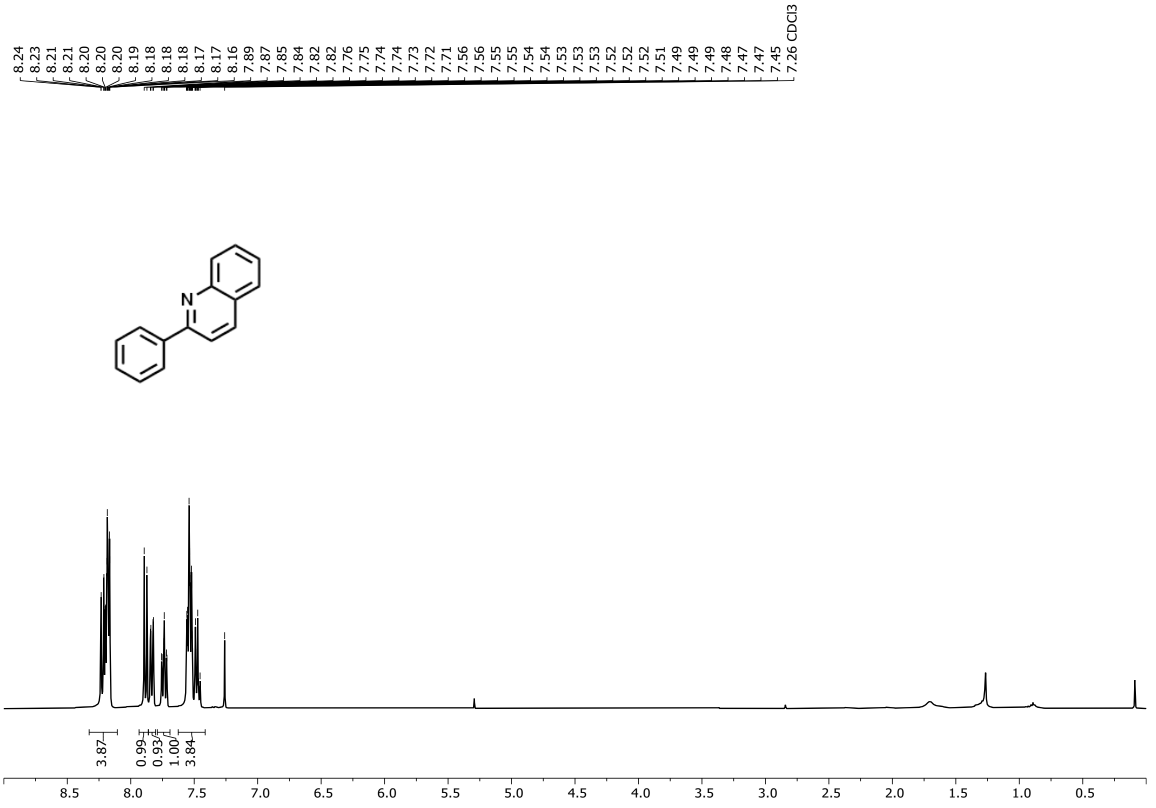

Spectrum 28 - ^{1}H NMR (400 MHz, $CDCl_3$) of compound ***2b***

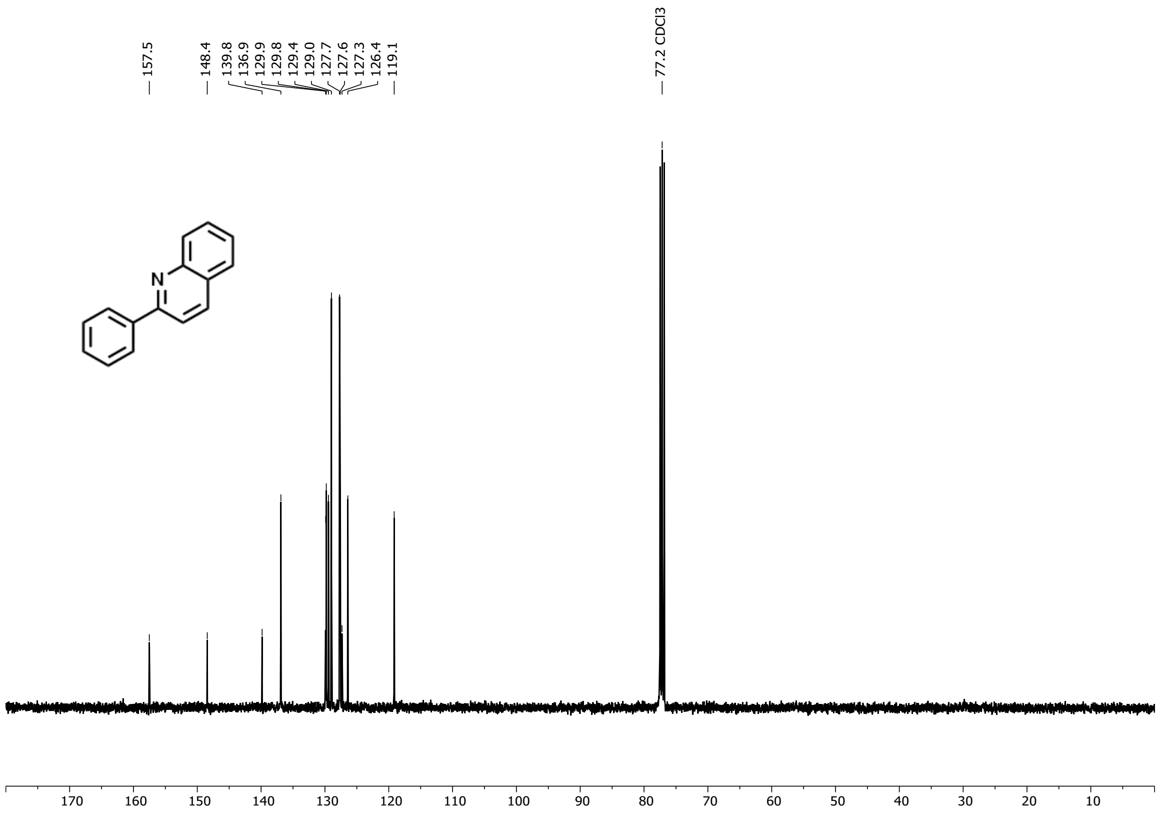

Spectrum 29 – ^{13}C NMR (101 MHz, $CDCl_3$) of compound ***2b***

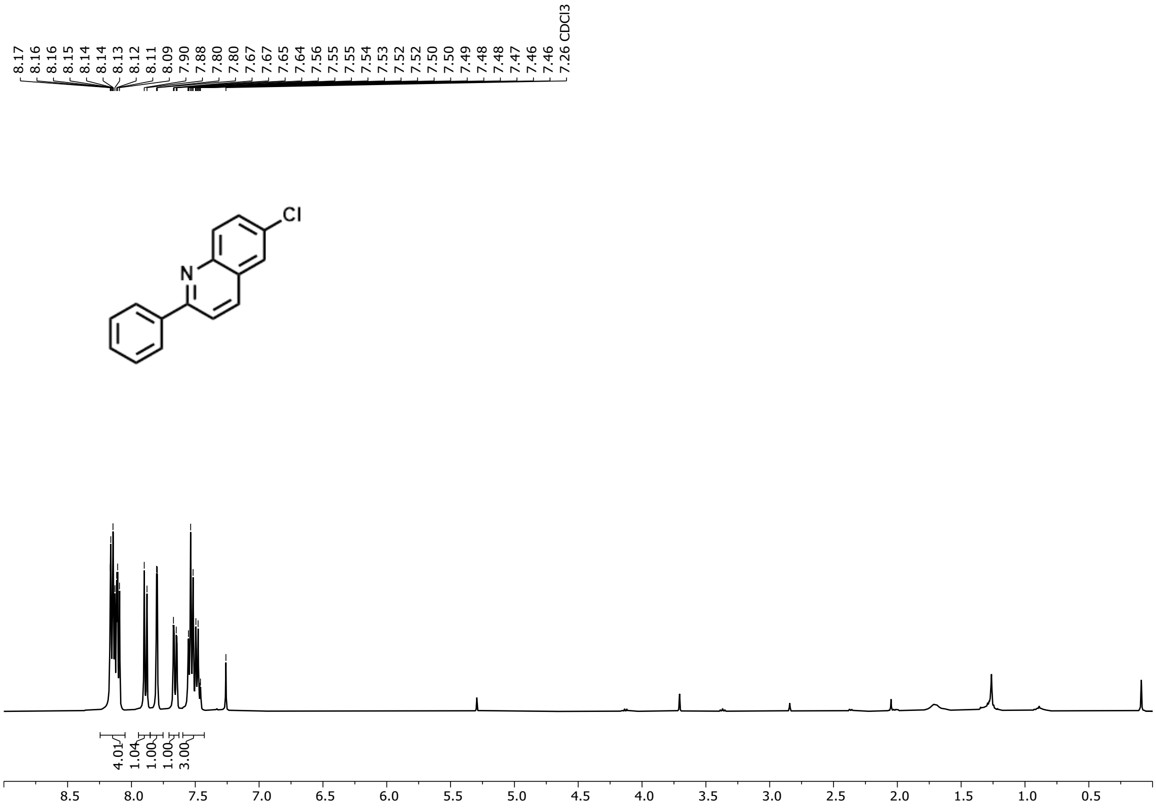

*Spectrum 30 - ^{1}H NMR (400 MHz, CDCl$_3$) of compound **2c***

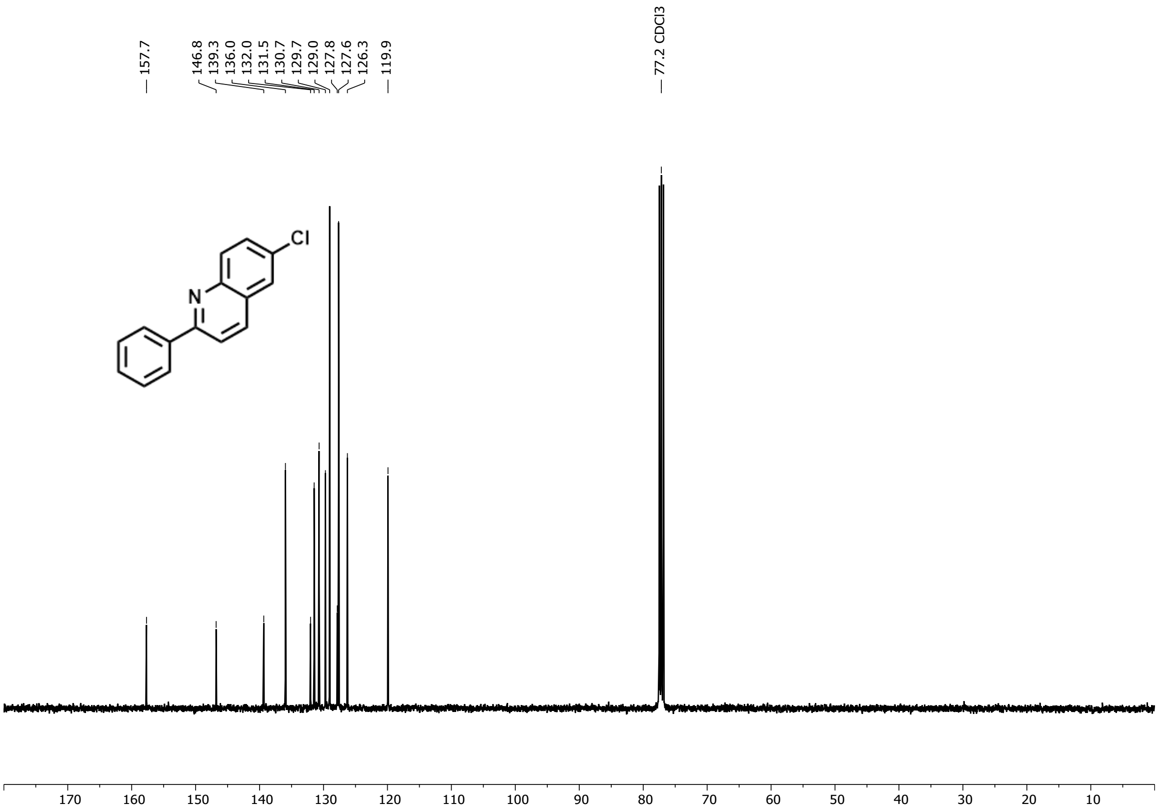

*Spectrum 31 – ^{13}C NMR (101 MHz, CDCl$_3$) of compound **2c***

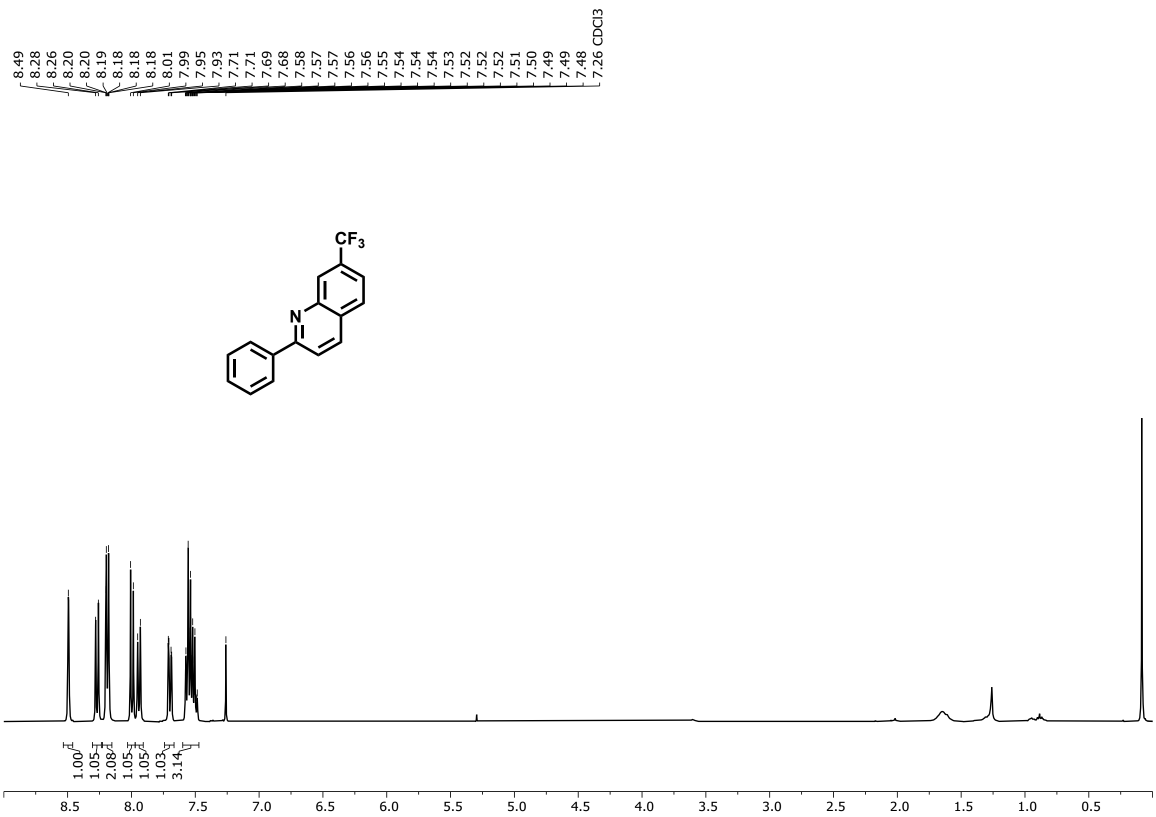

*Spectrum 32 - ^{1}H NMR (400 MHz, $CDCl_3$) of compound **2d***

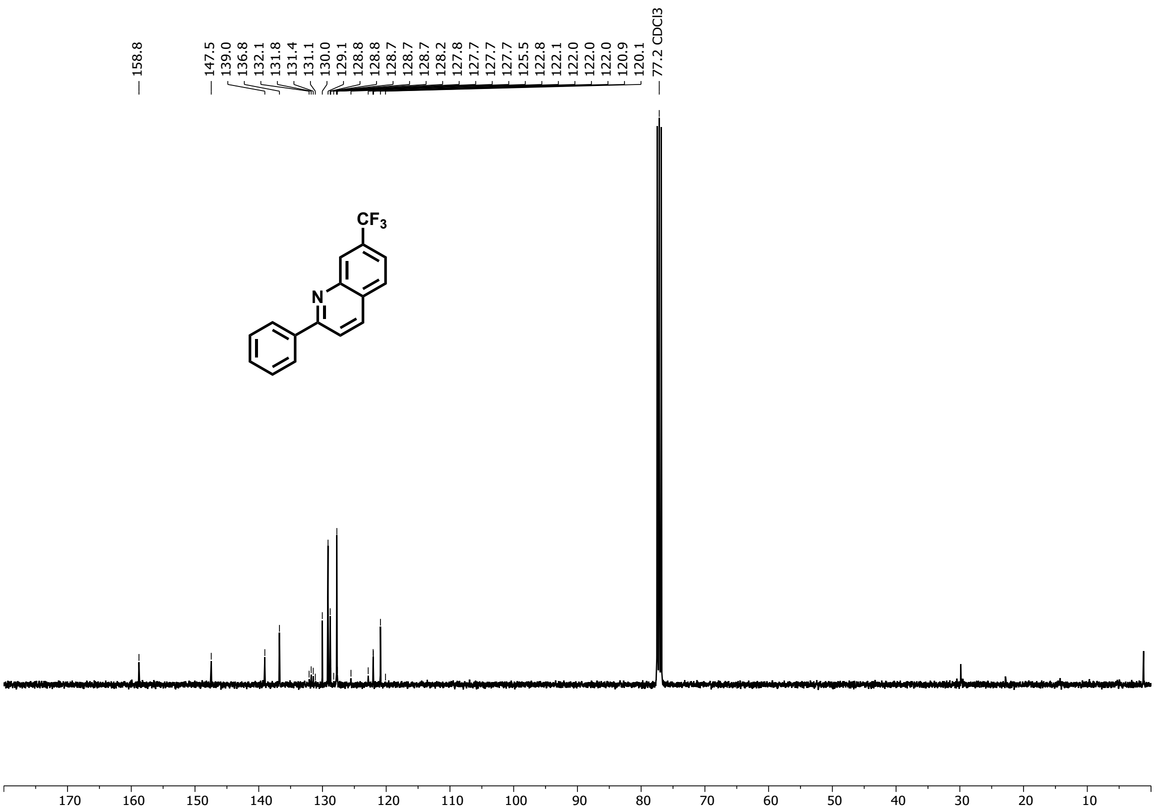

*Spectrum 33 - ^{13}C NMR (101 MHz, $CDCl_3$) of compound **2d***

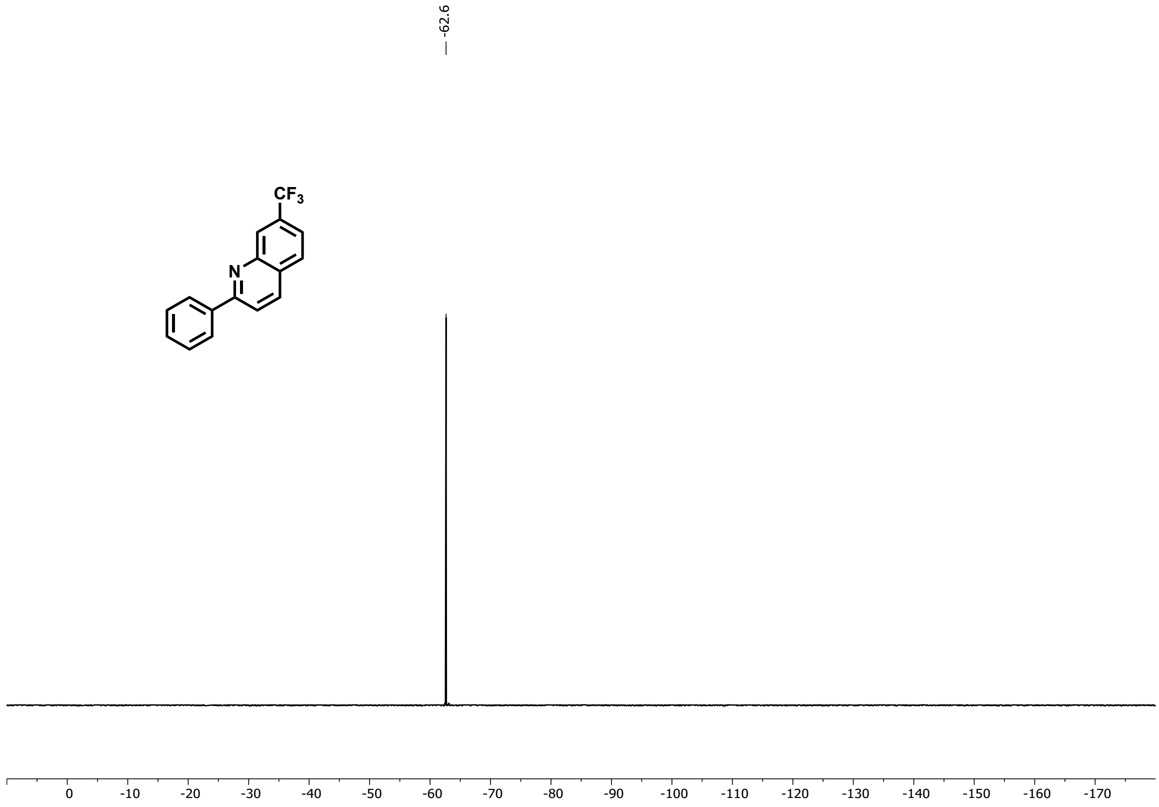

*Spectrum 34 – ^{19}F NMR (376 MHz, $CDCl_3$) of compound **2d***

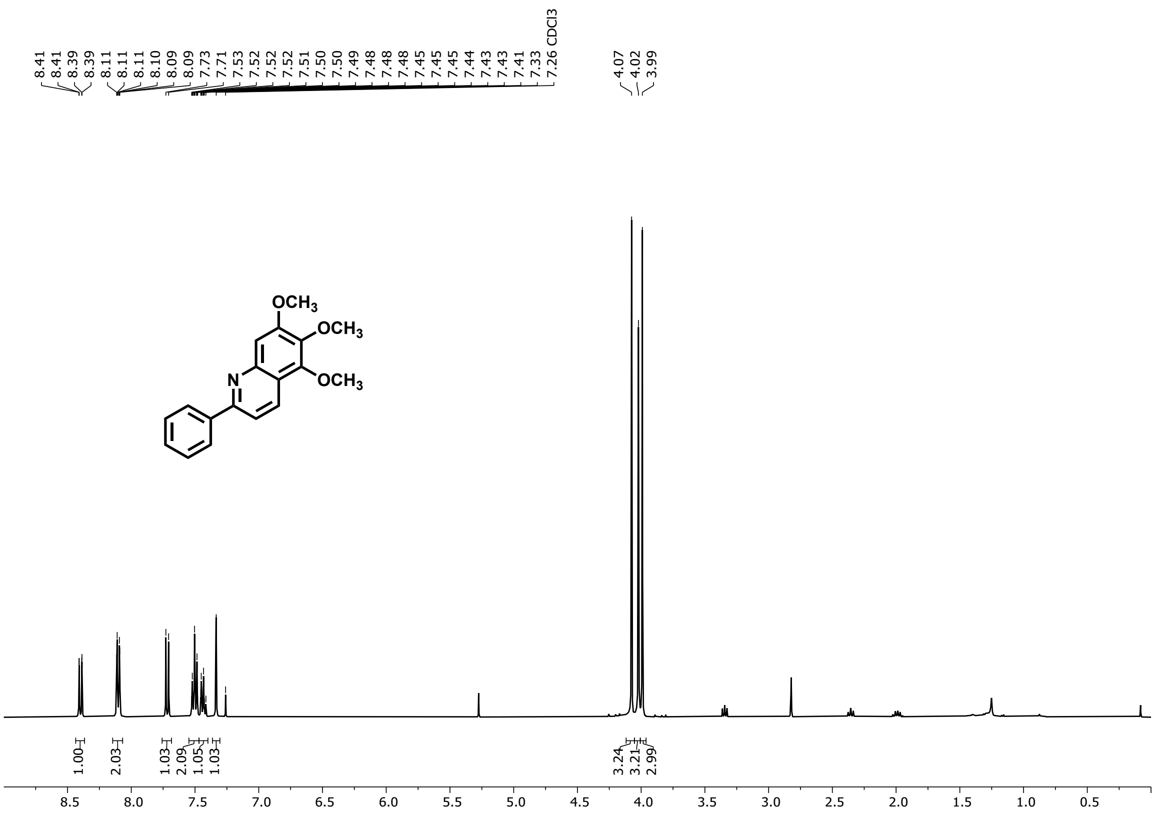

*Spectrum 35 - ^{1}H NMR (400 MHz, CDCl$_3$) of compound **2e***

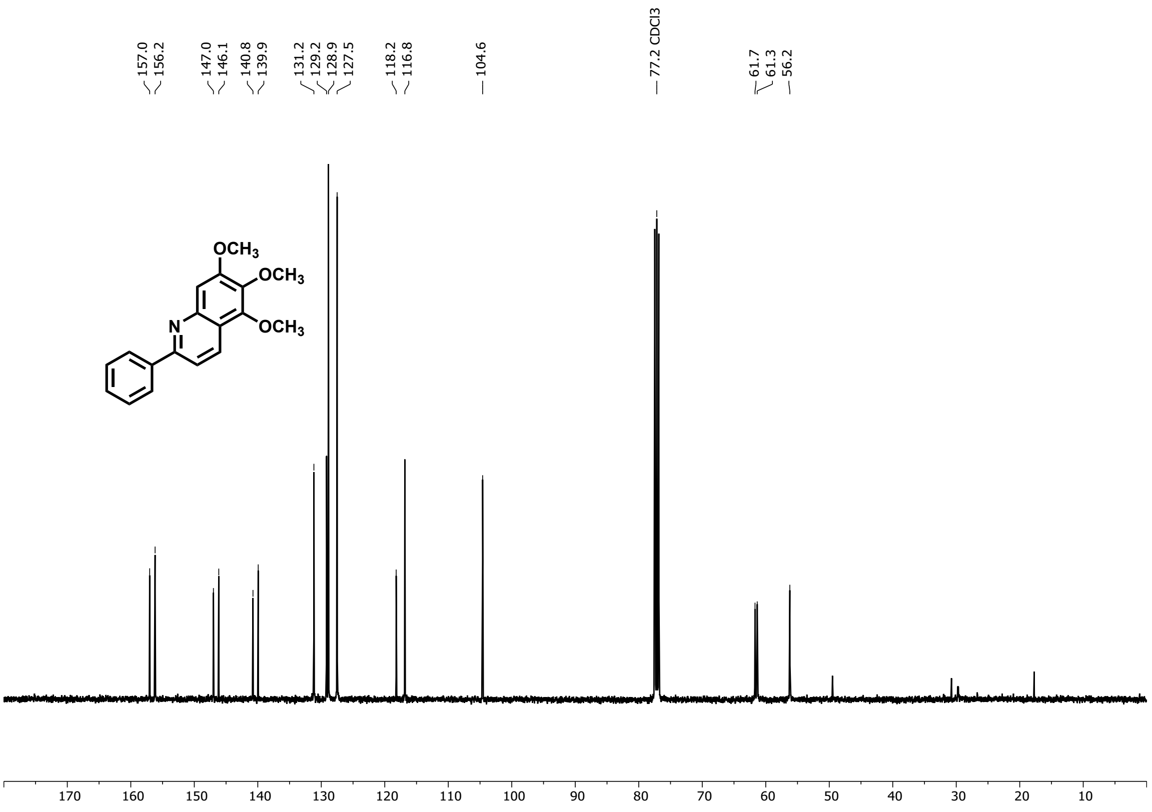

*Spectrum 36 - ^{13}C NMR (101 MHz, CDCl$_3$) of compound **2e***

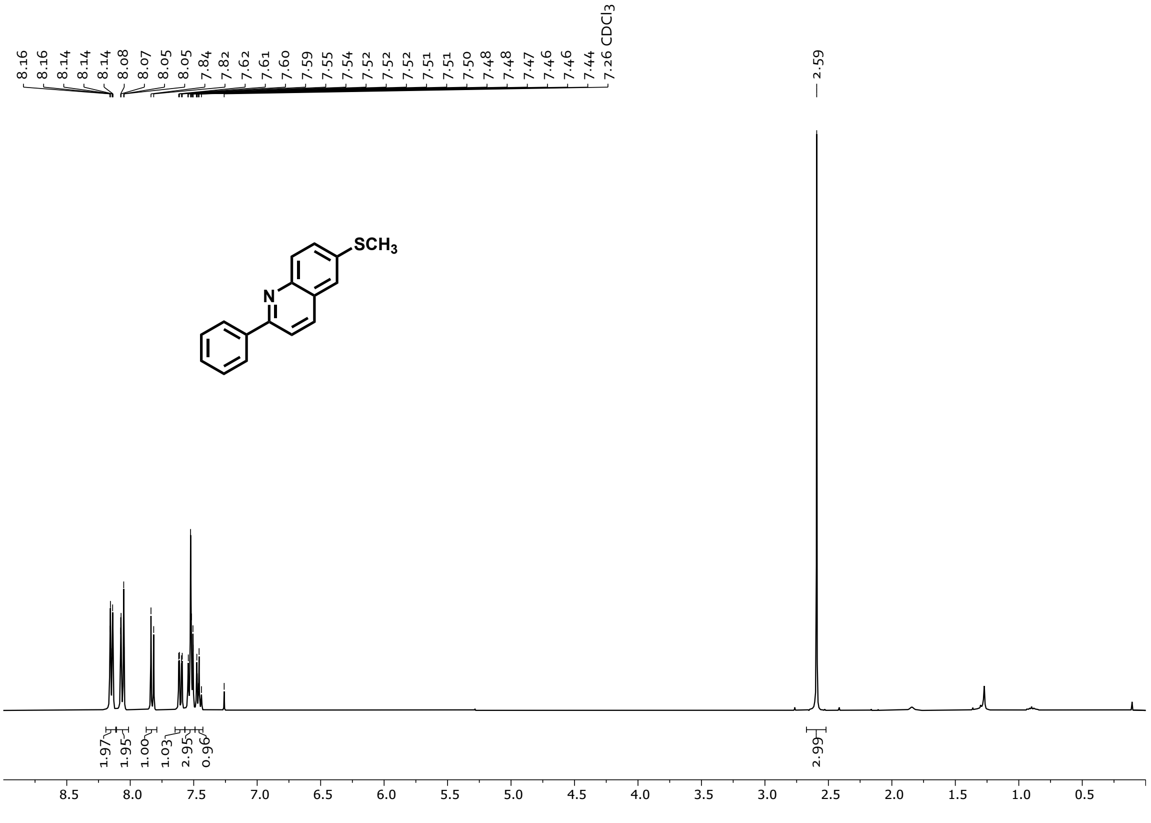

Spectrum 37 - ^{1}H NMR (400 MHz, $CDCl_3$) of compound ***2f***

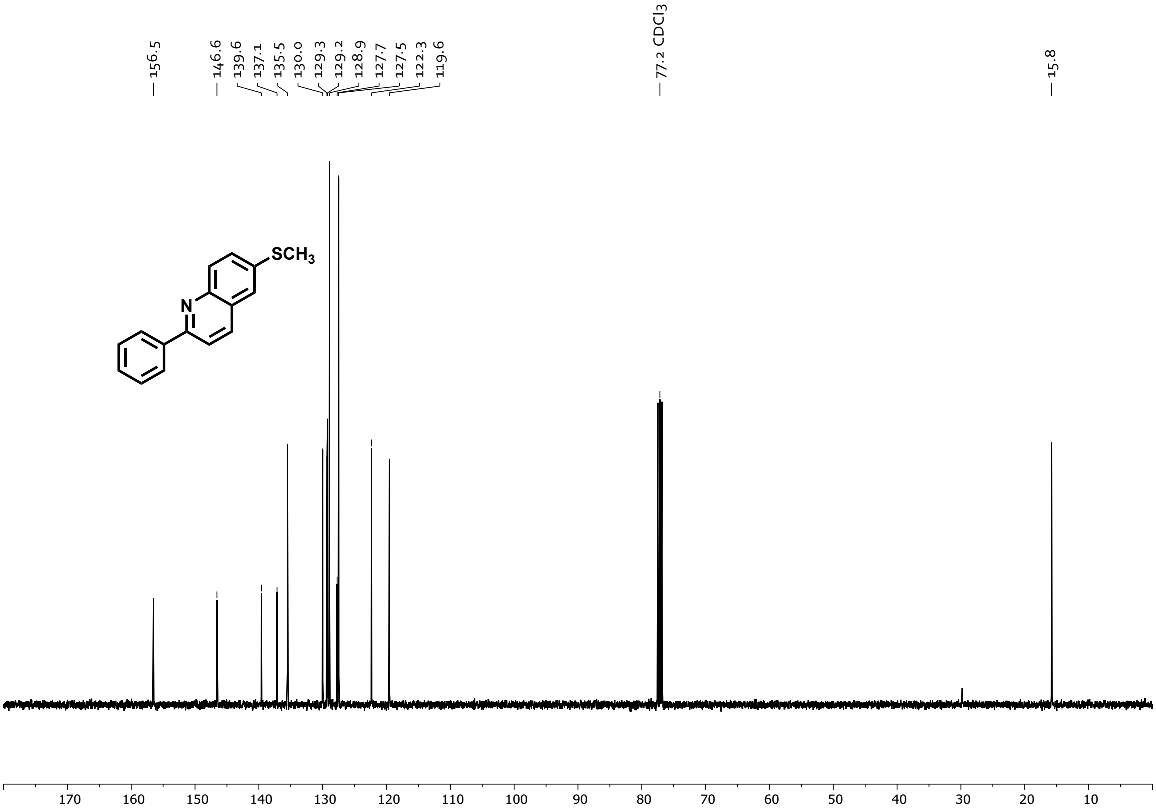

Spectrum 38 - ^{13}C NMR (101 MHz, $CDCl_3$) of compound ***2f***

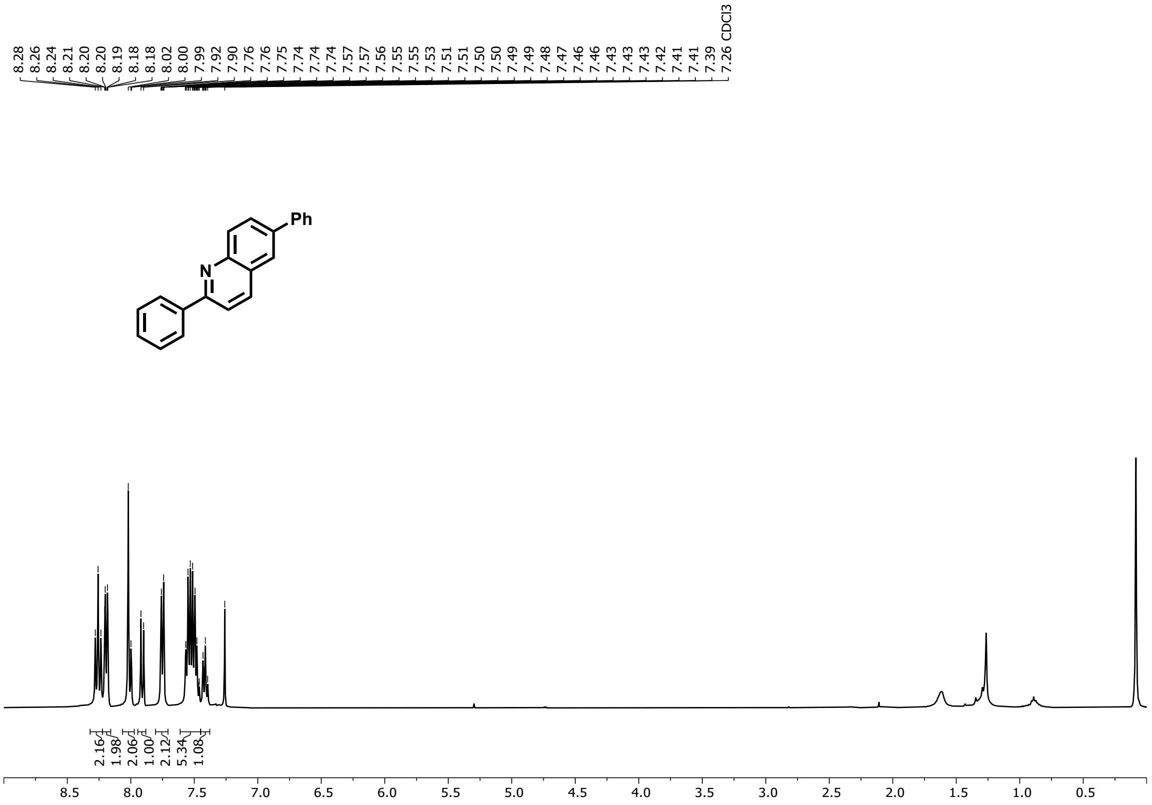

*Spectrum 39 - ^{1}H NMR (400 MHz, $CDCl_3$) of compound **2g***

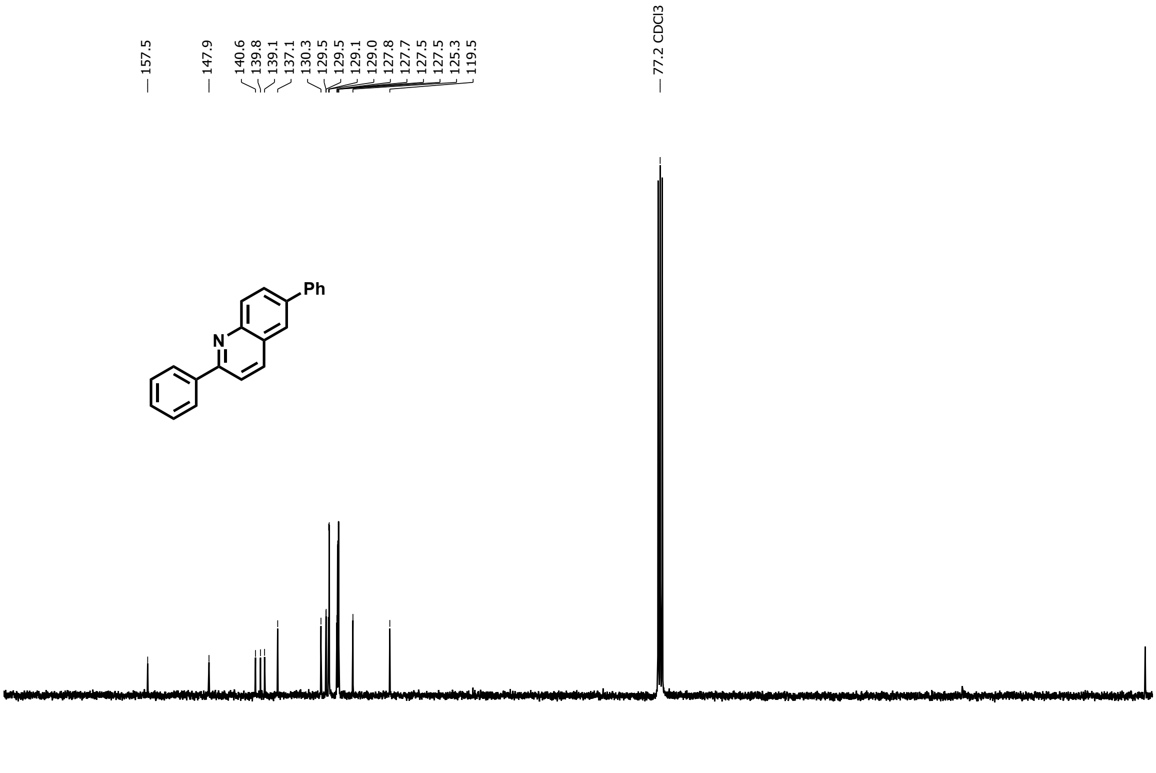

*Spectrum 40 -^{13}C NMR (101 MHz, $CDCl_3$) of compound **2g***

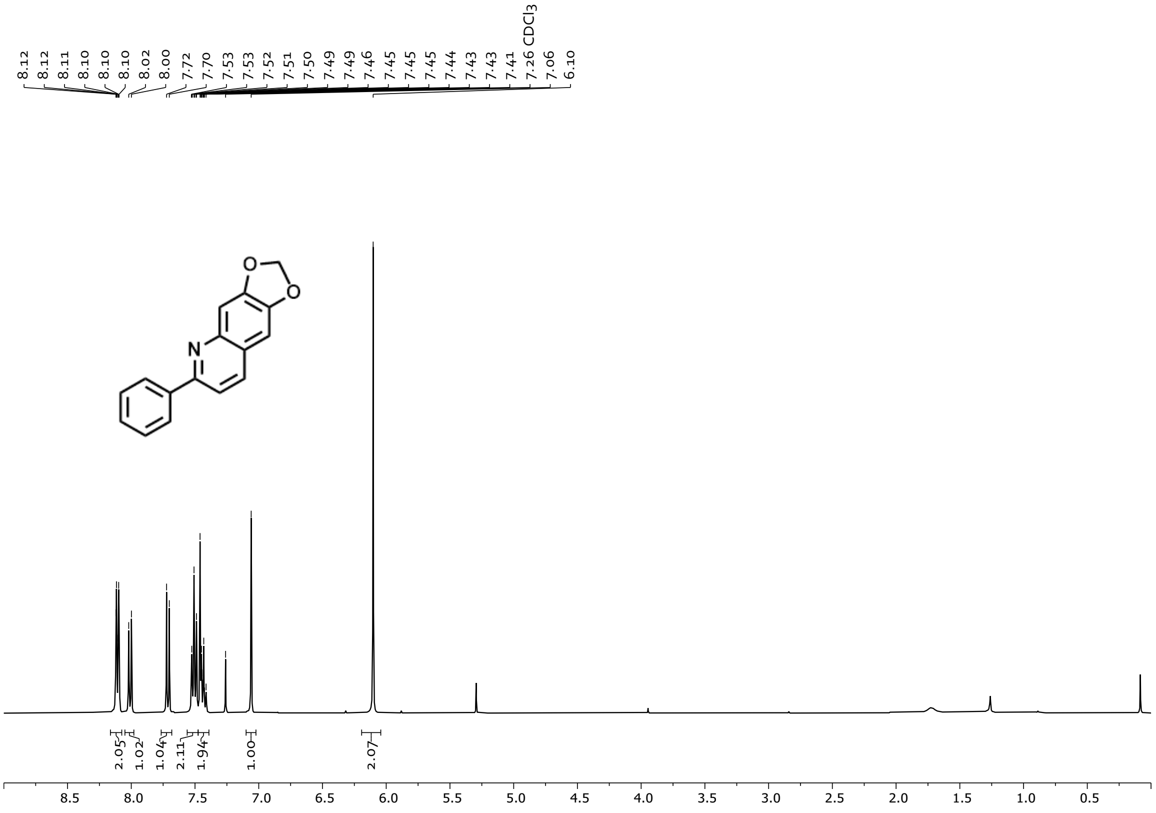

Spectrum 41 -¹H NMR (400 MHz, $CDCl_3$) of compound ***2h***

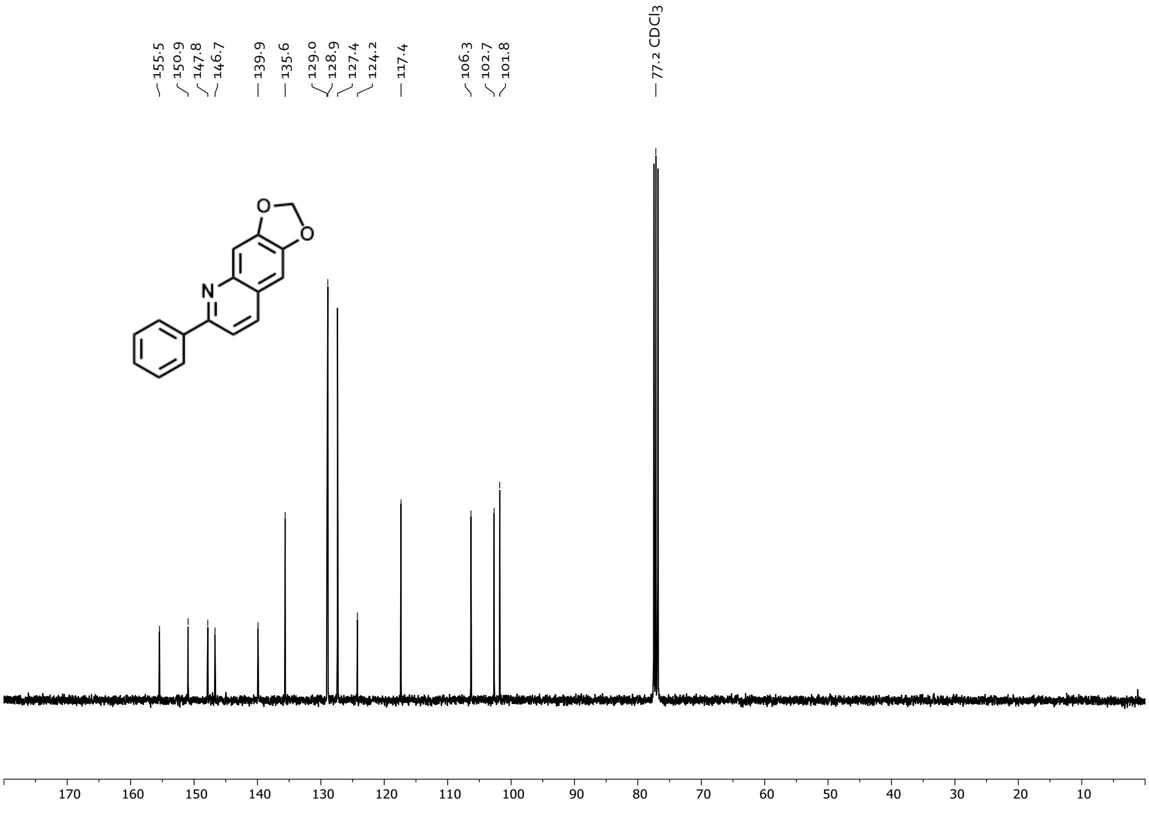

Spectrum 42 – ¹³C NMR (101 MHz, $CDCl_3$) of compound ***2h***

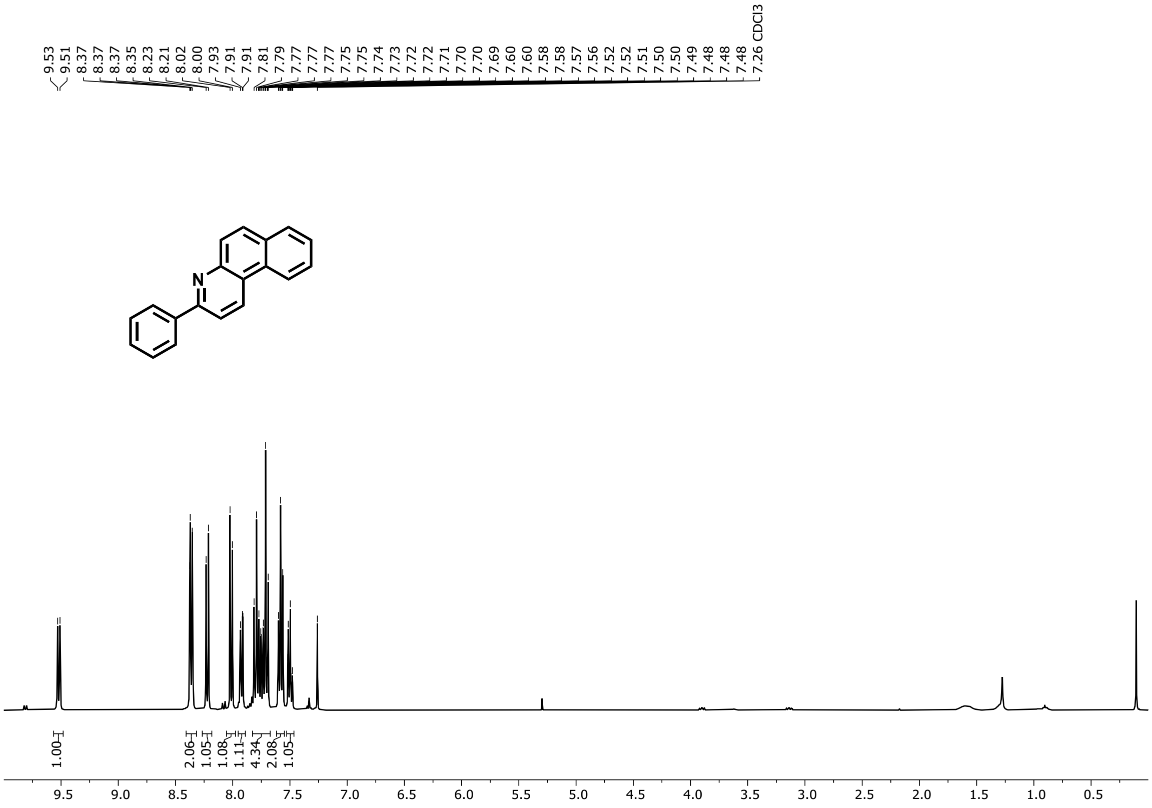

*Spectrum 43 - ^{1}H NMR (400 MHz, $CDCl_3$) of compound **2i***

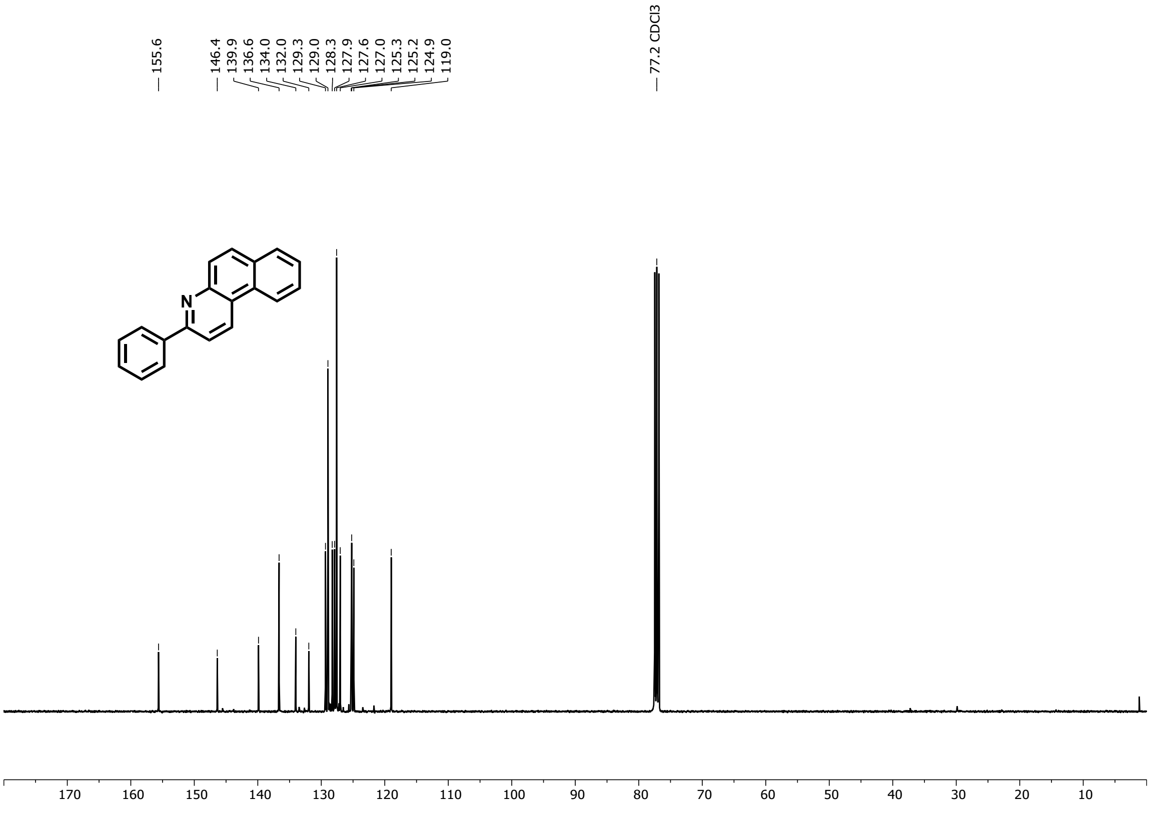

*Spectrum 44 - ^{13}C NMR (101 MHz, $CDCl_3$) of compound **2i***

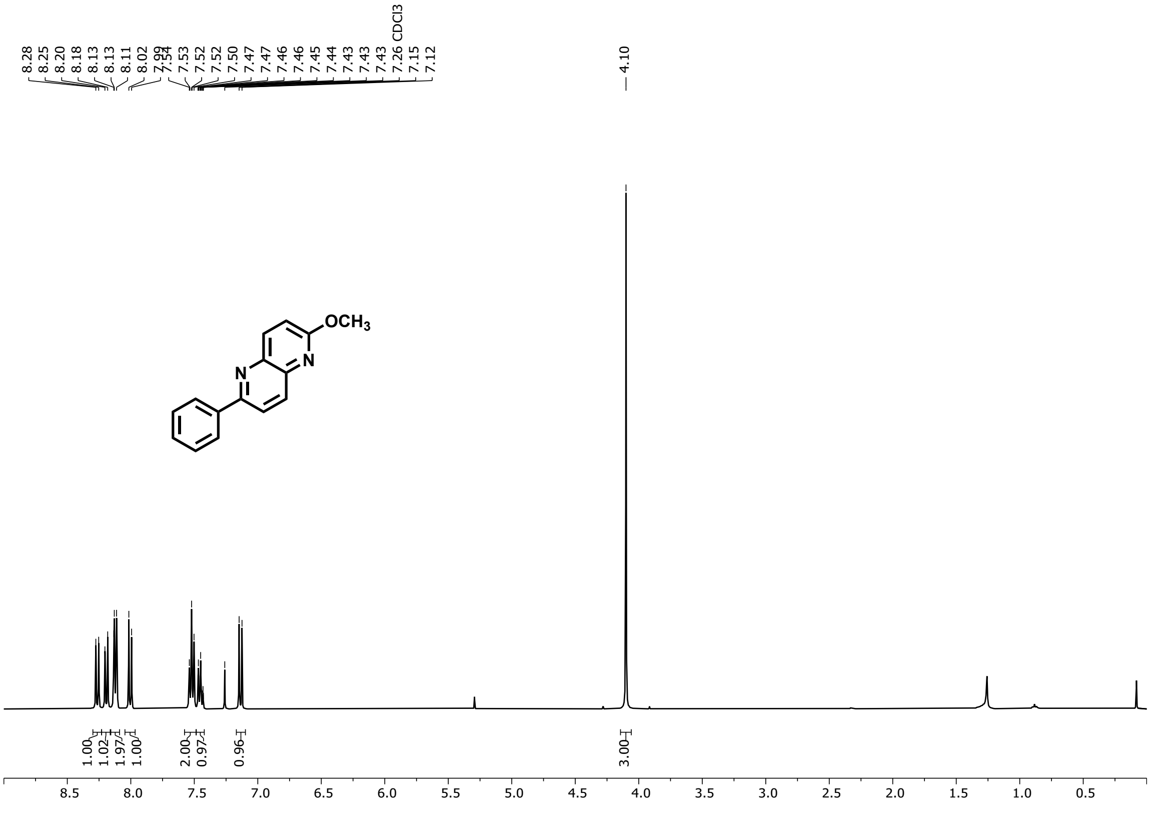

Spectrum 45 - ^{1}H NMR (400 MHz, $CDCl_3$) of compound ***2j***

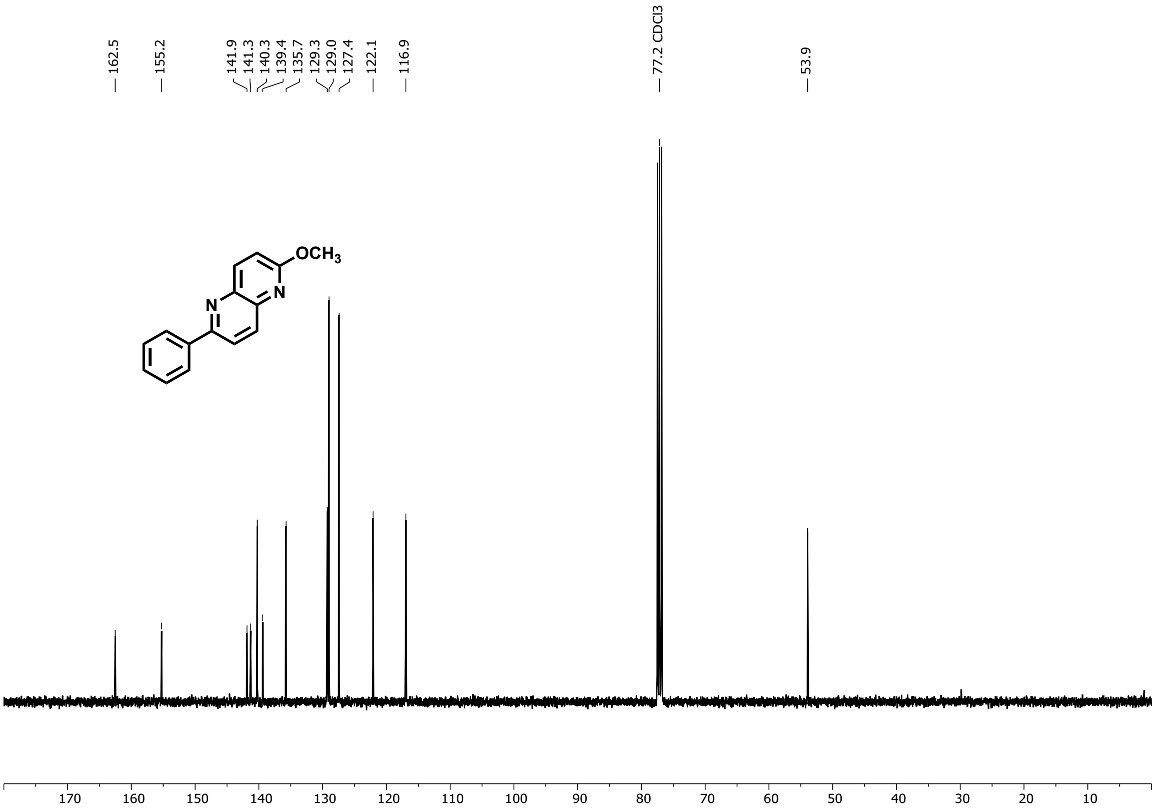

Spectrum 46 - ^{13}C NMR (101 MHz, $CDCl_3$) of compound ***2j***
